# Effects of age, sex, and ENSO phase on foraging and flight performance in Nazca boobies

**DOI:** 10.1002/ece3.7308

**Published:** 2021-03-10

**Authors:** Jennifer L. Howard, Emily M. Tompkins, David J. Anderson

**Affiliations:** ^1^ Department of Biology Wake Forest University Winston‐Salem NC USA

**Keywords:** accelerometer, airspeed, El Niño–Southern Oscillation, flapping, senescence, wingbeat frequency

## Abstract

Age‐related changes in survival and reproduction are common in seabirds; however, the underlying causes remain elusive. A lack of experience for young individuals, and a decline in foraging performance for old birds, could underlie age‐related variation in reproduction because reproductive success is connected closely to provisioning offspring. For seabirds, flapping flight during foraging trips is physiologically costly; inexperience or senescent decline in performance of this demanding activity might cap delivery of food to the nest, providing a proximate explanation for poor breeding success in young and old age, respectively. We evaluated the hypothesis that young and old Nazca boobies (*Sula granti*), a Galápagos seabird, demonstrate deficits in foraging outcomes and flight performance. We tagged incubating male and female adults across the life span with both accelerometer and GPS loggers during the incubation periods of two breeding seasons (years), during the 2015 El Niño and the following weak La Niña. We tested the ability of age, sex, and environment to explain variation in foraging outcomes (e.g., mass gained) and flight variables (e.g., wingbeat frequency). Consistent with senescence, old birds gained less mass while foraging than middle‐aged individuals, a marginal effect, and achieved a slower airspeed late in a foraging trip. Contrary to expectations, young birds showed no deficit in foraging outcomes or flight performance, except for airspeed (contingent on environment). Young birds flew slower than middle‐aged birds in 2015, but faster than middle‐aged birds in 2016. Wingbeat frequency, flap–glide ratio, and body displacement (approximating wingbeat strength) failed to predict airspeed and were unaffected by age. Sex influenced nearly all aspects of performance. Environment affected flight performance and foraging outcomes. Boobies' foraging outcomes were better during the extreme 2015 El Niño than during the 2016 weak La Niña, a surprising result given the negative effects tropical seabirds often experience during extreme El Niños.

## Graphical abstract

For seabirds, flapping flight during foraging trips is physiologically costly; inexperience or senescent decline in performance of this demanding activity might cap delivery of food to the nest, providing a proximate explanation for poor breeding success in young and old age, respectively. In this study, we evaluated age‐related variation in the foraging outcomes and flight performance of Nazca boobies (*Sula granti*), a long‐lived seabird breeding in Galápagos, during the 2015 El Niño and the following weak La Niña.

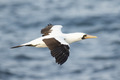

## 1 INTRODUCTION

Age‐related changes in survival and reproduction are nearly ubiquitous in long‐lived vertebrate animals (Clutton‐Brock, 1984; Jones et al., 2008; Nussey et al., 2013). Low, but improving, breeding success in young adults is typically followed by a period of high performance in middle age and then by low, and declining, breeding success and survival probabilities in old individuals (reviewed in Forslund & Pärt, 1995; Lemaître & Gaillard, 2017; Nussey et al., 2013). Deficits in experience for young adults, and in physiological ability for old adults, may each depress resource acquisition (Curio, 1983; Finkel & Holbrook, 2000). Under this hypothesis, age‐related changes in resource acquisition cap food delivery to self and to dependent young, contributing to early‐life improvements and late‐life declines in reproduction and survival, and motivating the study of age‐related changes in foraging success and the underlying array of physiological and behavioral traits that contribute to prey capture in the wild.

Pelagic seabirds offer an excellent model with which to evaluate early‐life improvement and late‐life decline in flight performance and resource acquisition. Many of these long‐lived species exhibit improving breeding success as young adults and declining breeding success in old age (Crespin et al., 2006; Pardo et al., 2013; Tompkins & Anderson, 2019), and foraging outcomes generally dictate reproductive success (via self‐maintenance throughout the breeding season and to support nestlings; e.g., Clifford & Anderson, 2001b; Gall et al., 2006; Regular et al., 2014). Their prey is typically at unpredictable and distant locations, suggesting a young adult's inexperience and an aging seabird's inability to meet the cost of flapping flight (Birt‐Friesen et al., 1989) as logical contributors to performance deficits (Forslund & Pärt, 1995). Despite widespread interest in proximate factors driving age‐related variation in breeding performance, empirical evaluation of underlying mechanisms remains scarce (Lemaître & Gaillard, 2017; but see Elliott et al., 2015).

Here, we examine age‐related variation in foraging success and flapping flight performance during commuting in incubating, known‐age Nazca boobies (*Sula granti*). This species shows pronounced age‐related variation in annual reproductive performance (a peak in middle age; Tompkins & Anderson, 2019; Tompkins et al., 2017). Food shortage accounts for most breeding failure in this species (Anderson et al., 2004; Clifford & Anderson, 2001b; Maness & Anderson, 2013); thus, foraging by this aerial predator connects individual characteristics (i.e., age) and oceanographic conditions with breeding success. The study occurred in two contrasting environments: the extreme El Niño–Southern Oscillation warm event (“El Niño”) in 2015–16 and the weak cool event (“La Niña”) in 2016–2017 (Santoso et al., 2017). El Niño conditions, especially warming of surface waters (up to 3°C in 2015–2016; Santoso et al., 2017), are typically associated with breeding failure and even adult mortality in tropical oceanic seabirds (Anderson, 1989; Boersma, 1998; Schreiber & Schreiber, 1984), but Nazca boobies exhibit a surprising positive effect of El Niño‐like conditions early in the breeding season (Tompkins & Anderson, 2021). We used electronic tags on free‐living boobies during their egg‐incubation period to evaluate the effects of age and environment on flight performance and foraging outcomes early in the breeding season, complementing the previous studies of age and breeding success.

Nazca boobies are pelagic central‐place foragers during their breeding season, flying hundreds of kilometers from the breeding colony in level flapping flight in search of unpredictable fish and squid prey (Zavalaga et al., 2012). They transport captured prey internally back to the nest to sustain themselves during incubation bouts of several days (this study) or to transfer to nest‐bound offspring (Anderson & Ricklefs, 1992). Their costly flap–glide flight mode requires a power output that exceeds five times their basal metabolic rate (Birt‐Friesen et al., 1989; Mullers et al., 2009). We focus on airspeed achieved during “commuting” flap–glide flight, on the forces imparted to the wings to generate thrust and lift, and on the rate of food delivery to sustain an incubation bout, asking if these performance metrics vary with age and environment in parallel with those observed for components of breeding performance. To our knowledge, this study is the first attempt to connect age‐related changes in foraging outcomes to underlying fine‐scale flight performance in any vertebrate.

## 2 MATERIALS AND METHODS

### 2.1 Tagging methods

We studied age effects on flight performance and foraging behavior at our long‐term study colony of Nazca boobies at Punta Cevallos on Isla Española, Galápagos (1°23′S, 89°37′W; Huyvaert and Anderson (2004) give details of the site). Annual banding of young of the year began in 1984, providing known‐age individuals. Breeding at Punta Cevallos is seasonal, with eggs laid from October to January and most nestlings fledging by the following June. Each breeding season is referred to by the first calendar year of its 2‐year sequence (i.e., the 2015–2016 season as “2015”). Egg incubation starts immediately after laying and incubation is continuous until hatching; pair members alternate incubation with time spent foraging. Sex‐specific vocalizations reliably indicate sex (Maness et al., 2007).

During the egg‐incubation period of two breeding seasons (2015 and 2016), we deployed GPS and accelerometer loggers on 241 breeding adults (123 females and 118 males) falling into four age categories corresponding to the demonstrated pattern of reproductive success (Anderson & Apanius, 2003; Tompkins & Anderson, 2019): “Young” (low success, 4–7 years), “Middle Age” (peak success, 12–14), “Old” (low success, 18–20), and “Oldest” (negligible success, 21+; max life span ≥28 years; unpublished data). To reduce temporal environmental noise, loggers were deployed on date‐matched triplets or quadruplets: a given deployment comprised a same‐sex cohort with one member of each age group (occasionally lacking an “oldest” member; sample sizes are broken down by sex and age group in Tables S1 and S2). Our deployment approach boosted the representation of Old and Oldest birds in our sample, relative to the overall breeding population. Sampled age ranges avoided gradual transitions between periods of high success (prime age) and low success (when young and during old age; Tompkins & Anderson, 2019), improving our ability to detect differences between age classes using a flexible multi‐level factor parameterization of age effects.

Axy‐Depth (Technosmart, Rome, Italy) loggers recorded acceleration data (sampling range ± 8 units of gravity; *g*) at 25 Hz on three axes—anterior–posterior (*x*‐axis), lateral (*y*‐axis), and dorsoventral (*z*‐axis)—and collected pressure and temperature at 1 Hz. I‐gotU® GT‐120 and GT‐600 (Mobile Action Technology, Taiwan) GPS loggers recorded location at 3‐min. intervals; they were deployed with the accelerometers, giving a combined logger mass (33 g and 48 g, respectively, 1.5%–3.2% of the tagged bird's mass) close to or below the recommended 3% of a bird's mass (range = 1,450–2,200 g; Kenward, 2000). Zavalaga et al. (2012) attached similar loggers to Nazca boobies with no effect on foraging trip duration. Loggers were removed at the completion of at least one observed “foraging absence,” defined here as a >3‐hour absence from the nest that ends with the bird returning to incubate the clutch. We distinguish a foraging absence (the period between ending one incubation shift and starting the next), from a “foraging trip” (the period between departing and returning to the colony), because a minority of birds completed a foraging trip and departed for another trip without incubating; that is, a foraging absence may comprise more than one foraging trip. Foraging outcomes (described below) were measured over a foraging absence to better reflect the total effort and total gain realized within the constraints of the incubation schedule. Flight component variables (see below) were measured on the scale of a foraging trip. Birds were weighed at logger deployment and retrieval; flattened, stretched wing chord (wrist to wing tip) was measured during retrieval. Data were analyzed for one foraging absence (foraging outcomes) or foraging trip (flight components) per bird, from the absence/trip with the most complete information (mass pre‐ and post‐trip and complete logger data). SI Methods Section 1 provides additional details of tagging.

### 2.2 Foraging outcomes

Aging patterns were examined for three foraging outcome variables: the duration of time a bird was foraging and absent from incubation duties (“Absence Duration”; hours), mass gained during the absent period (“Mass Gain”; grams), and their ratio mass gain rate (“Mass Gain/hr”; grams/hr). During the foraging absence, the total distance traveled and the absence's duration were highly correlated (Kendall *τ_b_* = 0.84, *n* = 194, *p* < .0001); therefore, we focused on Absence Duration only. Mass Gain was calculated by subtracting mass at departure (calculated from mass at logger deployment; SI Methods Section 6) from mass at return. Due to logger failures and some missing mass measurements, final sample sizes were 174 birds for Mass Gain and Mass Gain/hr, and 204 birds for Absence Duration.

### 2.3 Flight components

Data preparation and analysis were performed in R v. 4.0.2 (R Core Team, 2020) using scripts customized from Patterson et al. (2018). Nazca boobies alternate bouts of continuous flapping with bouts of fixed‐wing gliding (Figures S1 and S2). Four flight component response variables were extracted from a given trip's acceleration data (Figure S1): “Wingbeat Frequency” during flapping bouts; the ratio of the duration of a flapping bout and the subsequent gliding bout (“Flap‐Glide Ratio”); overall dynamic body acceleration (ODBA) per second of flapping (“Flapping ODBA”), a proxy for energy expenditure (Elliott, 2016; Elliott et al., 2013); and total vertical body displacement during a given downstroke–upstroke cycle (“Body Displacement”), a measure of wingbeat strength (Collins et al., 2020; Kogure et al., 2016). Body Displacement captures an equal and opposite reaction to the force imparted to the wings, and so reflects force developed in the axial part of the body (Shepard et al., 2008). Wingbeat Frequency, Flap–Glide Ratio, Flapping ODBA, and Body Displacement were mean values calculated from the first and last 30 minutes (“outbound” and “inbound,” respectively) of a foraging trip, when foragers typically use sustained level flapping flight to commute to and from foraging areas in a consistent flap–glide mode (Figure S2). The SI Methods Section 2 provides complete details of these calculations.

“Airspeed” was a fifth response variable, calculated as the mean of Airspeed measurements from each adjacent pair of GPS points within a given inbound or outbound period. Measures of groundspeed, a bird's bearing (heading angle between adjacent pairs of GPS points), wind speed, and wind direction were used to calculate Airspeed using equation 6 from Shamoun‐Baranes et al. (2007; see SI Methods equation 3). Groundspeed (movement relative to Earth's surface) is affected by flight behavior and by the corresponding winds (decreased by headwinds and increased by tailwinds, when all other factors are held constant; Liechti, 2006). Airspeed isolates movement due a bird's effort and airframe by controlling wind speed and direction relative to the bird's trajectory. We focused on Airspeed in this study because it is the direct result of the bird's physiological performance in self‐powered movement, and so is directly relevant to questions regarding senescent decline in generating thrust and lift. Later, we verified that results for "Groundspeed", which integrates Airspeed and “wind support” (Shamoun‐Baranes et al., 2007), aligned with those of Airspeed. Wingbeat Frequency, Flap–Glide Ratio, Body Displacement, and Flapping ODBA were assumed to influence a bird's breeding success through effects on prey delivery mediated by Airspeed. To evaluate this assumption, we used multiple linear regression models predicting Airspeed by Wingbeat Frequency, Flap–Glide Ratio, and Body Displacement to test for associations between the accelerometer‐derived flight components and Airspeed. Due to some logger failures, we obtained usable accelerometer‐derived flight component data from 209 outbound and 194 inbound birds, and Airspeed from 199 outbound and 172 inbound birds (Table S2).

### 2.4 Statistical analyses

We evaluated the effect of age on foraging outcomes (Absence Duration, Mass Gain, Mass Gain/hr), and flight components (Airspeed, Wingbeat Frequency, Flap–Glide Ratio, Body Displacement, Flapping ODBA) by evaluating mean differences in each response variable by “AgeGroup” (age was parameterized as a four‐level factor separating Young, Middle Age, Old, and Oldest birds). We used multiple linear regression models in an information theoretic approach to evaluate the hypothesis that age influences foraging outcomes, Airspeed, and accelerometer‐derived components of level flapping flight. We were especially interested in performance early in a trip (before the majority of the costly physiological effort and before refueling after a multi‐day incubation bout), and performance late in the trip (after the majority of the trip's effort and after taking on the extra mass of prey) when stamina effects would be most apparent. Therefore, flight components were evaluated separately for outbound and inbound periods.

The global model for all response variables included AgeGroup and up to five other predictors. Structural size affects the production of thrust and lift (Heerenbrink et al., 2015), influencing flight. Female Nazca boobies are larger than males (~17% by mass, 3.5% by wing chord; Figure S11) and experience steeper senescence for offspring production (Tompkins & Anderson, 2019), motivating the inclusion of factor “Sex” and its interaction with AgeGroup. Variation in structural size relative to sex‐specific mean values was included *via* the variable “Wing Loading”. Wing Loading was calculated as (9.8 * body mass)/(−0.20) + (0.92 * wing chord) and was centered within Sex; details are in the SI Methods Section 7. A dichotomous factor “Breeding Season” (2015 vs. 2016) was included in the global model to evaluate the effect of the extreme 2015 El Niño. A two‐way interaction between Breeding Season and AgeGroup allowed aging patterns to vary with environmental state, addressing the possibility that birds of different ages vary in their response to environmental quality (Tompkins and Anderson, 2021). Finally, two additional explanatory variables were not of primary interest but were included to control additional variation in foraging success and flight components. Wind support (Tailwind Component, “TWC”) was included as a predictor for flight components because wind affects flight behavior and airspeed (Kogure et al., 2016; Safi et al., 2013), influencing the cost of flight (Ballance, 1995). Negative values of TWC indicate a headwind and positive values a tailwind. Extended Julian Date (“Date”) accounted for seasonal changes across the 3‐month sampling period and was expressed as daily increments across each breeding season. Nazca boobies incubate their eggs for ~43 days (Anderson, 1993) and changes in parental motivation or behavior across this period could conceivably influence flight components and foraging success. However, the omission of a variable measuring placement within the incubation period (for each individual, at its date of departure on a foraging absence) from our analyses did not affect our results (see evaluation in Tables S5‐S9). All continuous variables were standardized (zero mean, unit standard deviation) before inclusion in models. Wing Loading was standardized by Sex and AgeGroup. We checked for collinearity between predictors (Table S4).

The performance of the global model for each response variable was compared to that of simpler candidate models that exclude one or more predictors, using AIC corrected for small sample size (AICc; Burnham & Anderson, 2002). Date appeared in all foraging outcome models, and Date and TWC appeared in all candidate models for flight components; all remaining combinations of nested submodels were included in the model set (26 total models per response variable). The best model explaining variation in the data (the “top model”) had the lowest AICc value. We considered all candidate models falling within 4 ΔAICc units of the top model highly supported (the “top model set”; Burnham et al., 2011), and we further evaluated predictors within the top model set using effect sizes and 95% confidence intervals (CIs) on coefficient estimates (Arnold, 2010). Our model set included interaction terms and explanatory variables that were weakly correlated (Table S4); for this reason, we do not model average regression coefficients (Banner & Higgs, 2017; Cade, 2015). Flap–Glide Ratio, Absence Duration, and Mass Gain/hr were log‐transformed before analysis to satisfy the assumption of normally distributed residuals.

## 3 RESULTS

Our results were complex, with multiple top models falling within 4 AICc increments of the top model. Our model set comprised nested models. Some predictors appeared intermittently within the model set and explained little variation in the response (effects were small in magnitude and uncertainty in the coefficient estimate included zero, based on the 95% CI). These appeared within the top model set because the penalty leveraged against the additional complexity was low (2 AICc units per one additional parameter). We focus on effects that were highly supported (appearing in top models and having 95% CIs distinct from zero), acknowledging uncertainty in the importance and direction of other predictors.

In overview, age effects were highly supported only for Airspeed, with late‐life declines in Airspeed that were limited to the inbound period and stronger in 2016 (an AgeGroup * Breeding Season interaction). In contrast, Breeding Season and Sex explained variation in most foraging outcomes and flight components. The 2015 El Niño was a favorable foraging environment for Nazca boobies: Absence Durations were markedly shorter than during 2016, driving improvements in Mass Gain/hr. Boobies had higher Flap–Glide Ratios, but lower Wingbeat Frequency, Body Displacement, and Flapping ODBA during the 2015 El Niño than in 2016. Turning to Sex, males, the smaller sex, gained less mass during a foraging absence. Males glided less (lower Flap–Glide Ratio), but flapped faster during a flapping bout, causing a higher Flapping ODBA. These results are described in detail below.

### 3.1 Foraging Outcomes

#### 3.1.1 Mass Gain/hr

**FIGURE 1:**
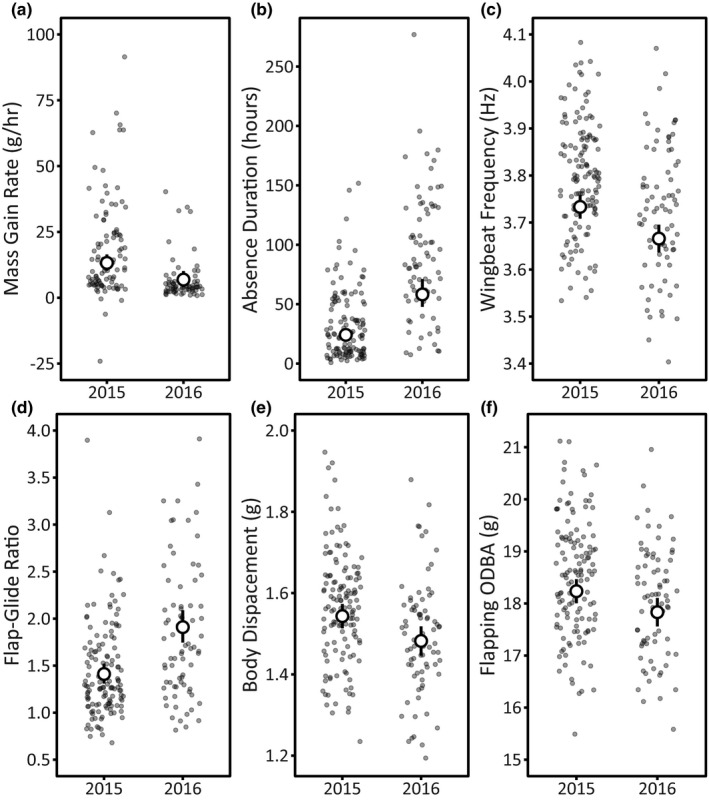
Effect of Breeding Season (2015—during a strong El Niño—vs. 2016) on foraging outcomes and flight components. Panels a‐b show foraging outcomes. Panels c‐f show outbound flight components; inbound Wingbeat Frequency and Flap–Glide Ratio follow the same pattern but are not shown here. We modeled log(Absence Duration), log(Mass Gain/hr), and log(Flap–Glide Ratio), but back‐transformed the predicted values to show effect size. Small points show the raw data (jittered horizontally); large points are model‐predicted means ± 95% CI from the top model explaining variation in each response variable, calculated holding the values of all other predictors at their mean (continuous covariates) or baseline level (factors)

Breeding Season affected Mass Gain/hr, appearing in all models in the top model set (Table 1). Rates of mass gain while foraging were higher during the 2015 El Niño than in 2016, during a weak La Niña (Figure 1). Other variables within the top model set (AgeGroup, Sex, and Wing Loading) had coefficient estimates not distinct from zero (based on 95% CIs; Table 2) and were absent from the top model (Table 1), leaving substantial uncertainty in the importance and direction of these effects on rates of mass gain while foraging. Much variation in Mass Gain/hr remained unexplained (*R* ^2^ = 0.14 for the top model).

**TABLE 1:** Top models explaining variation in foraging outcomes

Response variable	Fixed effects	*k*	ΔAICc	*ω_i_*
Log(Mass Gain/hr)	Season	4	0.00	0.35
	Wing Loading + Season	5	1.17	0.19
	Sex + Season	5	1.68	0.15
	Sex + Wing Loading + Season	6	2.84	0.08
	AgeG + Season	7	3.35	0.06
Mass Gain	Sex + Wing Loading	5	0.00	0.45
	Sex + Wing Loading + Season	6	1.02	0.27
	AgeG + Sex + Wing Loading	8	2.93	0.10
	AgeG + Sex + Wing Loading + Season	9	3.87	0.06
Log(Absence Duration)	Wing Loading + Season	5	0.00	0.63
	Sex + Wing Loading + Season	6	2.02	0.23

Note

Top models fall within ΔAICc of 4; Tables S10‐S12 contain complete model rankings. The number of parameters (*k*), AICc difference from the top model (ΔAICc), and Akaike weights (*ω_i_*) are reported. AgeG = AgeGroup; Season = Breeding Season. Date appeared in all models and is not shown.

**TABLE 2:** Coefficient estimates, and *SE,* from the top model set explaining variation in each foraging outcome

Response variable	Coefficient estimates (*β* (*SE*))							
	Intercept	AgeGroup (Young)	AgeGroup (Old)	AgeGroup (Oldest)	Sex (males)	Breeding Season (2016)	Wing Loading	Date
Log(Mass Gain/hr)	**1.58 (0.02)**	–	–	–	–	**−0.08 (0.03)**	–	**0.04 (0.01)**
	**1.59 (0.02)**	–	–	–	–	**−0.07 (0.03)**	0.003 (0.003)	**0.04 (0.01)**
	**1.57 (0.02)**	–	–	–	0.02 (0.03)	**−0.08 (0.03)**	–	**0.04 (0.01)**
	**1.57 (0.02)**	–	–	–	0.02 (0.03)	**−0.07 (0.03)**	0.003 (0.003)	**0.04 (0.01)**
	**1.62 (0.03)**	−0.06 (0.04)	−0.04 (0.04)	−0.04 (0.04)	–	**−0.09 (0.03)**	–	**0.04 (0.01)**
Mass Gain	**366.58 (14.88)**	–	–	–	**−55.71 (21.35)**	–	**−13.77 (2.00)**	**27.15 (10.99)**
	**356.28 (17.84)**	–	–	–	**−55.49 (21.35)**	24.65 (23.54)	**−13.05 (2.11)**	**29.31 (11.18)**
	**393.46 (23.37)**	−17.90 (29.51)	−38.16 (28.74)	−53.90 (31.68)	**−57.71 (21.41)**	–	**−14.21 (2.03)**	**27.88 (11.08)**
	**380.43 (26.14)**	−11.91 (29.98)	−38.54 (28.72)	−52.28 (31.69)	**−57.22 (21.40)**	26.70 (24.05)	**−13.41 (2.15)**	**30.37 (11.30)**
Log(Absence Duration)	**1.38 (0.03)**	–	–	–	–	**0.38 (0.06)**	**−0.03 (0.01)**	**−0.06 (0.03)**
	**1.39 (0.04)**	–	–	–	−0.02 (0.05)	**0.38 (0.06)**	**−0.03 (0.01)**	**−0.06 (0.03)**

Note

Here, and in subsequent tables, AgeGroup coefficients describe the mean difference in performance for Young, Old, and Oldest age classes relative to Middle Age. Sex coefficient describes the mean difference in performance for males relative to females. Breeding Season coefficient describes the mean difference in performance for birds in 2016 relative to 2015. Models are ordered from smallest to largest ΔAICc. Coefficients in bold have 95% CI that exclude zero.

#### 3.1.2 Mass Gain

**FIGURE 2:**
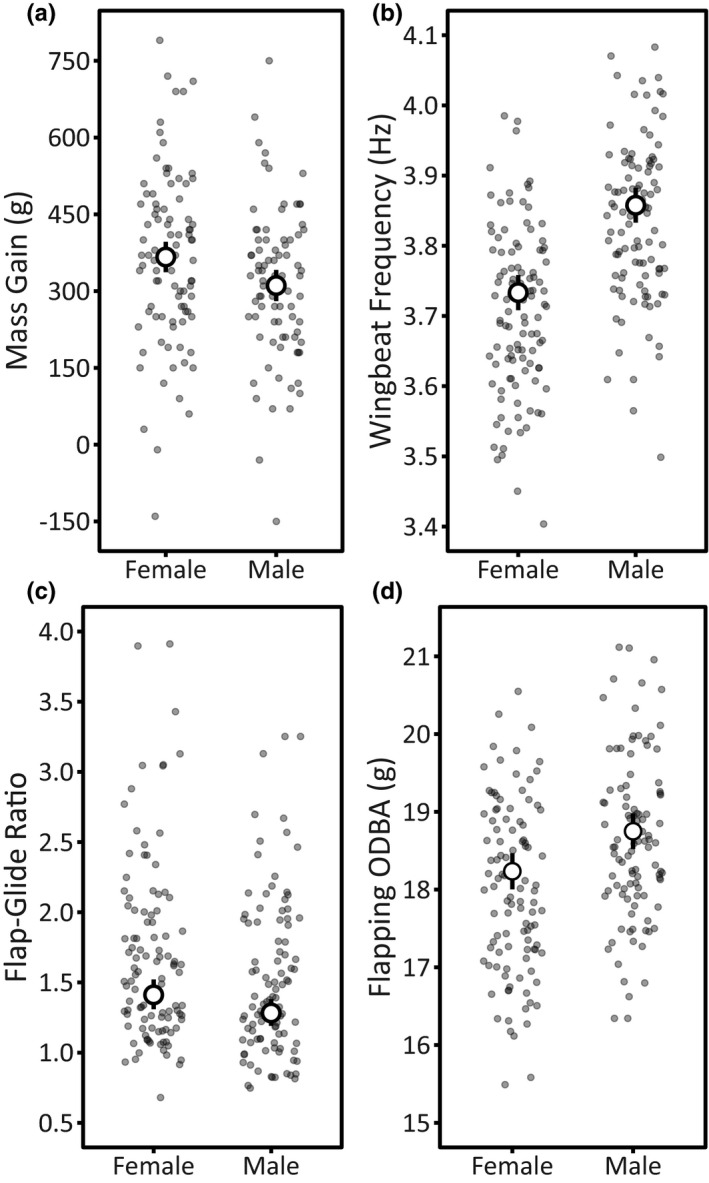
Sex differences in (a) Mass Gain, (b) Wingbeat Frequency (outbound; same pattern for inbound), (c) Flap–Glide Ratio (outbound only), and (d) Flapping ODBA (outbound only) in Nazca boobies. We modeled log(Flap–Glide Ratio), but back‐transformed the predicted values to show effect size. Small points show the raw data (jittered horizontally), and large points are model‐predicted means ± 95% CI from the top model, with 95% CIs on Sex distinct from zero, calculated holding the values of all other predictors at their mean (continuous covariates) or baseline level (factors; Tables 2 and 5). See Table S19 for mean values

Sex and Wing Loading affected Mass Gain, appearing in all models in the top model set (Table 1). Females gained 17.9% more mass over a foraging absence than males did (Figure 2a). For each sex, a higher Wing Loading decreased Mass Gain (Table 2). Model selection results provided weak evidence for late‐life decline in Mass Gain: Middle Age birds had higher Mass Gain than Old (*β* = −38.16 [95% CI: −94.91, 18.58]) and Oldest birds (*β* = −53.90 [95% CI: −116.44, 8.64]), although the 95% CIs narrowly include zero and AgeGroup was absent from the top model (Table 1). Breeding Season was absent from the top model and the 95% CIs included zero (Table 2). The top model explained 26% of the variation in Mass Gain (*R* ^2^ = 0.26).

#### 3.1.3 Absence Duration

Breeding Season and Wing Loading explained variation in Absence Duration, appearing in all models in the top model set (Table 1). Absence Durations of these incubating boobies were shorter (halved) during the 2015 El Niño, congruent with patterns for Mass Gain/hr (Figure 1; Table 2). Absence Duration decreased with increasing Wing Loading (Table 2). Sex appeared in the top model set, but the influence of Sex included zero (Table 2). AgeGroup did not appear in the top model set (Table 1). The top model explained 45% of variation in Absence Duration (*R* ^2^ = 0.45).

### 3.2 Flight components

#### 3.2.1 Airspeed

**FIGURE 3:**
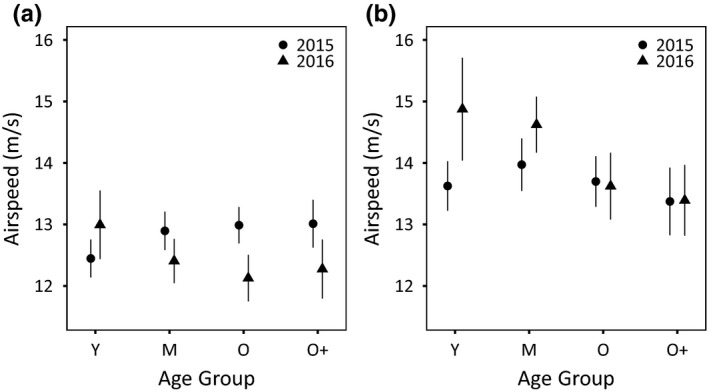
Effects of Breeding Season and AgeGroup on the (a) outbound and (b) inbound Airspeed in Young (Y), Middle Age (M), Old (O), and Oldest (O+) Nazca boobies. Points are model‐predicted means ± 95% CI from the top model, as in Figure 1

On the outbound, Breeding Season explained variation in Airspeed, appearing in all models in the top model set (Table 3). Outbound Airspeeds were faster during the 2015 El Niño. AgeGroup appeared in the top model (conditioned by an interaction with Breeding Season), but not in two other models in the top model set (Table 3). Model selection uncertainty was probably driven by the restriction of age effects to early life (Table 4). Young birds flew slower than Middle Age and older birds in 2015, but flew faster than others in 2016 (95% CIs support this result: Figure 3a; Table 4). Sex appeared within the top model set, but the 95% CI on Sex included zero (Table 4). The top model explained a large portion of the variation in Airspeed (*R* ^2^ = 0.71).

**TABLE 3:** Top models explaining variation in flight components

Response variable	Outbound				Inbound			
	Fixed effects	*k*	ΔAICc	*ω_i_*	Fixed effects	*k*	ΔAICc	*ω_i_*
Airspeed	AgeG * Season	11	0.00	0.34	Sex + Wing Loading + AgeG * Season	13	0.00	0.33
	Wing Loading + AgeG * Season	12	0.60	0.25	Wing Loading + AgeG * Season	12	0.48	0.26
	Sex + AgeG * Season	12	2.26	0.11	AgeG + Sex + Wing Loading + Season	10	1.31	0.17
	Season	5	2.57	0.09	AgeG + Wing Loading + Season	9	2.01	0.12
	Sex + Wing Loading + AgeG * Season	13	2.89	0.08				
	Wing Loading + Season	6	3.89	0.05				
Wingbeat Frequency	Sex + Season	6	0.00	0.60	Sex + Wing Loading + Season	7	0.00	0.54
	Sex + Wing Loading + Season	7	1.92	0.23	Sex + Season	6	0.86	0.35
Log(Flap–Glide Ratio)	Sex + Wing Loading + Season	7	0.00	0.67	Wing Loading + Season	6	0.00	0.42
	Wing Loading + Season	6	2.32	0.21	Sex + Wing Loading + Season	7	1.45	0.20
					Season	5	3.36	0.08
					Wing Loading + AgeG * Season	12	3.50	0.07
Body Displacement	Sex + Wing Loading + Season	7	0.00	0.25	Season	5	0.00	0.19
	Sex + Season	6	0.69	0.18	(Base model)	4	0.41	0.16
	AgeG + Sex + Wing Loading + Season	10	1.09	0.14	Wing Loading + Season	6	0.70	0.14
	Wing Loading + Season	6	1.56	0.11	Wing Loading	5	1.47	0.09
	Season	5	2.10	0.09	Sex + Season	6	2.10	0.07
	AgeG + Wing Loading + Season	9	2.92	0.06	AgeG + Season	8	2.31	0.06
	AgeG + Sex + Season	9	3.27	0.05	Sex	5	2.49	0.06
	Sex + Wing Loading + AgeG * Season	13	3.73	0.04	Sex + Wing Loading + Season	7	2.82	0.05
					AgeG + Wing Loading + Season	9	2.98	0.04
					Sex + Wing Loading	6	3.57	0.03
					AgeG	7	3.70	0.03
Flapping ODBA	Sex + Season	6	0.00	0.49	(Base model)	4	0.00	0.23
	Sex + Wing Loading + Season	7	2.12	0.17	Sex	5	1.32	0.12
	AgeG + Sex + Season	9	2.33	0.15	Wing Loading	5	1.56	0.11
					Season	5	1.58	0.11
					AgeG	7	2.26	0.08
					Sex + Wing Loading	6	2.91	0.05
					Sex + Season	6	2.93	0.05
					Wing Loading + Season	6	3.05	0.05
					AgeG + Sex	8	3.60	0.04
					AgeG + Wing Loading	8	3.84	0.03

Note

Top models fall within ΔAICc of 4; Tables S13‐S17 contain complete model rankings. The number of parameters (*k*), AICc difference from the top model (ΔAICc), and Akaike weights (*ω_i_*) are reported. AgeG = AgeGroup; Season = Breeding Season. Tailwind component (TWC) and Date appeared in all models. Main effects with interactions are simplified (e.g., AgeG + Season + AgeG * Season is written as “AgeG * Season”).

**TABLE 4:** Coefficient estimates, and *SE*, from the top model set explaining variation in each flight component during the outbound period

Response variable	Coefficient estimates (*β* (*SE*))									
	Intercept	AgeG (Young)	AgeG (Old)	AgeG (Oldest)	AgeG * Season (Young, 2016)	AgeG * Season (Old, 2016)	AgeG * Season (Oldest, 2016)	Sex (males)	Season (2016)	Wing Loading
Airspeed	**12.90 (0.16)**	**−0.45 (0.22)**	0.09 (0.22)	0.12 (0.25)	**1.04 (0.40)**	−0.37 (0.34)	−0.25 (0.39)	–	**−0.49 (0.24)**	–
	**12.86 (0.16)**	**−0.45 (0.22)**	0.09 (0.22)	0.17 (0.25)	**1.07 (0.40)**	−0.36 (0.34)	−0.28 (0.39)	–	−0.43 (0.24)	0.016 (0.013)
	**12.90 (0.17)**	**−0.45 (0.22)**	0.09 (0.22)	0.12 (0.25)	**1.04 (0.40)**	−0.37 (0.34)	−0.25 (0.39)	−0.01 (0.13)	**−0.49 (0.24)**	–
	**12.82 (0.08)**	–	–	–	–	–	–	–	**−0.44 (0.14)**	–
	**12.87 (0.17)**	**−0.45 (0.22)**	0.09 (0.22)	0.17 (0.25)	**1.07 (0.40)**	−0.36 (0.34)	−0.28 (0.39)	−0.01 (0.13)	−0.43 (0.24)	0.016 (0.013)
	**12.81 (0.09)**	–	–	–	–	–	–	–	**−0.40 (0.15)**	0.011 (0.013)
WBF	**3.73 (0.01)**	–	–	–	–	–	–	**0.12 (0.02)**	**−0.07 (0.02)**	–
	**3.73 (0.01)**	–	–	–	–	–	–	**0.13 (0.02)**	**−0.07 (0.02)**	0.001 (0.001)
Log(FGR)	**0.15 (0.02)**	–	–	–	–	–	–	**−0.04 (0.02)**	**0.13 (0.02)**	**0.005 (0.002)**
	**0.13 (0.01)**	–	–	–	–	–	–	–	**0.13 (0.02)**	**0.005 (0.002)**
Body Displacement	**1.54 (0.02)**	–	–	–	–	–	–	0.04 (0.02)	**−0.06 (0.02)**	0.003 (0.002)
	**1.55 (0.02)**	–	–	–	–	–	–	0.03 (0.02)	**−0.07 (0.02)**	–
	**1.53 (0.02)**	0.01 (0.03)	−0.01 (0.02)	0.05 (0.03)	–	–	–	0.04 (0.02)	**−0.06 (0.02)**	0.004 (0.002)
	**1.56 (0.01)**	–	–	–	–	–	–	–	**−0.06 (0.02)**	0.003 (0.002)
	**1.56 (0.01)**	–	–	–	–	–	–	–	**−0.07 (0.02)**	–
	**1.55 (0.02)**	0.01 (0.03)	−0.01 (0.02)	0.05 (0.03)	–	–	–	–	**−0.06 (0.02)**	0.004 (0.002)
	**1.54 (0.02)**	0.01 (0.03)	−0.01 (0.02)	0.04 (0.03)	–	–	–	0.04 (0.02)	**−0.07 (0.02)**	–
	**1.53 (0.02)**	0.01 (0.03)	−0.01 (0.02)	**0.09 (0.04)**	0.02 (0.06)	0.02 (0.05)	−0.08 (0.06)	**0.04 (0.02)**	−0.05 (0.03)	0.004 (0.002)
Flapping ODBA	**18.24 (0.12)**	–	–	–	–	–	–	**0.52 (0.14)**	**−0.40 (0.15)**	–
	**18.23 (0.12)**	–	–	–	–	–	–	**0.52 (0.14)**	**−0.40 (0.16)**	0.002 (0.01)
	**18.07 (0.17)**	0.37 (0.20)	0.18 (0.19)	0.03 (0.21)	–	–	–	**0.52 (0.14)**	**−0.36 (0.15)**	–

Note

Models are ordered from smallest to largest ΔAICc. Coefficients in bold have 95% CI that exclude zero. Results for fixed predictors Date and TWC are reported in Table S18. AgeG = AgeGroup; Season = Breeding Season.

Age effects were also apparent on inbound Airspeed (birds returning to the colony), appearing in all top models (with Breeding Season and Wing Loading; Table 3). In contrast with results from the outbound period, inbound Airspeed declined in old age; the Old and Oldest age classes had slower Airspeed than Middle Age birds, particularly in 2016 (AgeGroup interacted with Breeding Season in the top model; Figure 3b; Table 5). Airspeed increased with Wing Loading (Table 5). Sex appeared within the top model set, but the 95% CI on Sex included zero (Table 5). The top model explained 54% of the variation in Airspeed (*R* ^2^ = 0.54).

**TABLE 5:** Coefficient estimates from the top model set explaining variation in each flight component during the inbound period

Response variable	Fixed effects (*β* (*SE*))									
	Intercept	AgeG (Young)	AgeG (Old)	AgeG (Oldest)	AgeG * Season (Young, 2016)	AgeG * Season (Old, 2016)	AgeG * Season (Oldest, 2016)	Sex (males)	Season (2016)	Wing Loading	
Airspeed	**13.93 (0.21)**	−0.35 (0.27)	−0.27 (0.27)	−0.60 (0.33)	0.60 (0.54)	−0.73 (0.43)	−0.63 (0.48)	−0.26 (0.16)	**0.65 (0.29)**	**0.041 (0.012)**	
	**13.83 (0.20)**	−0.34 (0.27)	−0.26 (0.27)	−0.59 (0.33)	0.60 (0.54)	−0.76 (0.43)	−0.62 (0.48)	–	**0.66 (0.29)**	**0.040 (0.012)**	
	**14.01 (0.19)**	−0.32 (0.24)	**−0.56 (0.21)**	**−0.90 (0.24)**	–	–	–	−0.27 (0.16)	**0.40 (0.18)**	**0.041 (0.012)**	
	**12.82 (0.08)**	−0.31 (0.24)	**−0.56 (0.21)**	**−0.88 (0.24)**	–	–	–	–	**0.40 (0.18)**	**0.041 (0.012)**	
Wingbeat Frequency	**3.76 (0.02)**	–	–	–	–	–	–	**0.11 (0.02)**	**−0.07 (0.02)**	0.003 (0.002)	
	**3.76 (0.02)**	–	–	–	–	–	–	**0.11 (0.02)**	**−0.08 (0.02)**	–	
Log(Flap–Glide Ratio)	**0.22 (0.01)**	–	–	–	–	–	–	–	**0.08 (0.02)**	**0.004 (0.002)**	
	**0.21 (0.02)**	–	–	–	–	–	–	0.02 (0.02)	**0.08 (0.02)**	**0.004 (0.002)**	
	**0.22 (0.01)**	–	–	–	–	–	–	–	0.08 (0.02)	–	
	**0.23 (0.02)**	−0.003 (0.04)	−0.006 (0.03)	−0.04 (0.04)	−0.03 (0.07)	−0.06 (0.06)	0.11 (0.06)	–	**0.08 (0.04)**	**0.004 (0.002)**	
Body Displacement	**1.43 (0.01)**	–	–	–	–	–	–	–	−0.03 (0.02)	–	
	**1.42 (0.01)**	–	–	–	–	–	–	–	–	–	
	**1.43 (0.01)**	–	–	–	–	–	–	–	−0.04 (0.02)	−0.002 (0.002)	
	**1.42 (0.01)**	–	–	–	–	–	–	–	–	−0.002 (0.002)	
	**1.43 (0.02)**	–	–	–	–	–	–	0.00 (0.02)	−0.03 (0.02)	–	
	**1.43 (0.02)**	−0.02 (0.03)	0.02 (0.03)	0.04 (0.03)	–	–	–	–	−0.04 (0.02)	–	
	**1.42 (0.01)**	–	–	–	–	–	–	0.00 (0.02)	–	–	
	**1.43 (0.02)**	–	–	–	–	–	–	0.00 (0.02)	−0.04 (0.02)	−0.002 (0.002)	
	**1.43 (0.02)**	−0.02 (0.03)	0.02 (0.03)	0.04 (0.03)				–	−0.04 (0.02)	−0.002 (0.002)	
	**1.42 (0.01)**	–	–	–	–	–	–	0.00 (0.02)	–	−0.002 (0.002)	
	**1.41 (0.02)**	−0.01 (0.03)	0.02 (0.03)	0.04 (0.03)	–	–	–	–	–	–	
Flapping ODBA	**17.65 (0.07)**	–	–	–	–	–	–	–	–	–	
	**17.59 (0.10)**	–	–	–	–	–	–	0.13 (0.15)	–	–	
	**17.65 (0.07)**	–	–	–	–	–	–	–	–	−0.01 (0.01)	
	**17.69 (0.09)**	–	–	–	–	–	–	–	−0.11 (0.15)	–	
	**17.47 (0.13)**	0.18 (0.21)	0.37 (0.19)	0.26 (0.22)	–	–	–	–	–	–	
	**17.59 (0.10)**	–	–	–	–	–	–	0.13 (0.15)	–	−0.01 (0.01)	
	**17.63 (0.12)**	–	–	–	–	–	–	0.13 (0.15)	−0.11 (0.15)	–	
	**17.70 (0.09)**				–	–	–	–	−0.12 (0.15)	−0.01 (0.01)	
	**17.40 (0.15)**	0.18 (0.21)	0.37 (0.19)	0.26 (0.22)	–	–	–	0.13 (0.15)	–	–	
	**17.47 (0.13)**	0.18 (0.21)	0.37 (0.19)	0.25 (0.22)	–	–	–	–	–	−0.01 (0.01)	

Note

Models are ordered from smallest to largest ΔAICc. Coefficients in bold have 95% CI that exclude zero. Results for fixed predictors Date and TWC are reported in Table S18. AgeG = AgeGroup; Season = Breeding Season.

#### 3.2.2 Wingbeat Frequency

On the outbound and inbound periods, Breeding Season and Sex appeared in all top models explaining variation in Wingbeat Frequency (Table 3). Birds flapped faster in 2015 than in 2016 (Figure 1; Tables 4 and 5). Males flapped faster than females (Figure 2b; Tables 4 and 5). Wing Loading also appeared in the outbound and inbound top model sets, but the influence of size was not distinct from zero in either period (Tables 4 and 5). Top models explained similar variation in outbound (*R* ^2^ = 0.30) and inbound (*R* ^2^ = 0.24) Wingbeat Frequency.

#### 3.2.3 Flap–Glide Ratio

On the outbound, Breeding Season and Wing Loading explained variation in the Flap–Glide Ratio, appearing in all models in the top model set (Table 3). During the 2015 El Niño, birds spent a smaller proportion of time flapping relative to gliding compared to 2016 (Figure 1; Table 4). The proportion of time spent flapping during the flap–glide cycle increased with increasing Wing Loading (Table 4). Sex appeared in the top model: males spent less time flapping during flap–glide cycles compared to females (Figure 2c; Table 4). Much variation in outbound Flap–Glide Ratio remained unexplained (*R* ^2^ = 0.19 for the top model). On the inbound, Breeding Season appeared in all models within the top model set, matching the outbound result (Table 3). Wing Loading appeared in most models within the top model set, including the top model. The time spent flapping during the flap–glide cycle increased with increasing Wing Loading (Table 5). Other predictors within the top model set (AgeGroup, AgeGroup * Season, and Sex) were absent from the top model and had 95% CIs on coefficient estimates that included zero (Table 5). Most variation in Flap–Glide Ratio remained unexplained (*R* ^2^ = 0.19 for the top model).

#### 3.2.4 Body Displacement

Substantial uncertainty in model selection existed for Body Displacement on both the outbound and the inbound (Table 3). Breeding Season was the only variable to appear in all candidate models for the outbound period (Table 3), reflecting more applied force during the 2015 El Niño (Figure 1; Table 4). Breeding Season did not affect Body Displacement on the inbound period (Table 5). For both outbound and inbound, other predictors (AgeGroup, Sex, and Wing Loading) in the top model sets had 95% CIs on coefficient estimates including zero, and multiple top models excluded these effects (Tables 4 and 5). On the outbound (*R* ^2^ = 0.15) and inbound (*R* ^2^ = 0.05), the top models explained little variation in this trait.

#### 3.2.5 Flapping ODBA

On the outbound, Breeding Season and Sex explained variation in Flapping ODBA, appearing in all models in the top model set (Table 3). Flapping ODBA—a proxy for energy expenditure while flapping—was lower during the El Niño in 2015 (Figure 1). Males had higher ODBA during flaps than females did (all 95% CIs distinct from zero; Figure 2d; Table 4). AgeGroup and Wing Loading also appeared within the top model set (Table 4), but the 95% CIs of these coefficients spanned zero and several top models excluded these effects, leaving substantial uncertainty in the importance and direction of age and size effects on Flapping ODBA. Most variation in Flapping ODBA remained unexplained on the outbound (*R* ^2^ = 0.19 for the top model). On the inbound, substantial uncertainty in model selection existed, and the base model with Date and TWC (predictors appearing in all models) was the top model (Table 3). The top model explained only 4% of variation in Flapping ODBA on the inbound (*R* ^2^ = 0.04).

### 3.3 Airspeed and accelerometer‐derived flight components

Wingbeat Frequency, Flap–Glide Ratio, Body Displacement, and Flapping ODBA did not explain variation in Airspeed (Table S20). This surprising result motivated us to question our assumption that these accelerometer‐derived flight components capture individual differences in physiological aspects of force generation. We lack direct measurements of muscle performance to compare with these variables, so we calculated their repeatabilities, reasoning that if Wingbeat Frequency, Flap–Glide Ratio, Body Displacement, and Flapping ODBA are controlled partially by each individual's structure and physiology, then these variables should be repeatable (low intra‐individual variance, high interindividual variance). We estimated the individual‐level repeatability of each component using repeated measures in the R package *rptR* (Nakagawa & Schielzeth, 2010; Stoffel et al., 2017). The repeatability index (*R*) ranges from 0 (low repeatability, high within‐individual variance) to 1 (high repeatability, low within‐individual variance). All flapping component variables were repeatable during level flapping flight (Table S21). However, repeatabilities for Flap–Glide Ratio (*R* < 0.17), Wingbeat Frequency (*R* < 0.30), and Flapping ODBA (*R* < 0.30) were relatively low during both inbound and outbound periods. In contrast, repeatabilities for Body Displacement were high (outbound *R* = 0.45 [0.40, 0.49], *p* = .001; inbound *R* = 0.65 [0.60, 0.69], *p* = .001).

## 4 DISCUSSION

This study evaluated age‐related variation in foraging and flight performance in a pelagic seabird during two contrasting oceanographic environments. Surprisingly, we found strong evidence of age effects only on Airspeed, and conditioned by Breeding Season. On the outbound, Young birds flew slower than Middle Age birds in 2015 and faster than all other age classes in 2016. On the inbound, older birds flew slower than Middle Age birds at the end of a foraging trip (consistent with a deficit in stamina). Wingbeat kinematics offered little explanation for the age effects on Airspeed. Male and female Nazca boobies showed clear sex differences in flapping characteristics. Boobies' flight performance and foraging outcomes differed in the contrasting foraging environments of the extreme 2015 El Niño and the weak 2016 La Niña, suggesting that the foraging environment in 2015 was substantially more favorable.

### 4.1 Age‐related variation in foraging outcomes

**FIGURE 4:**
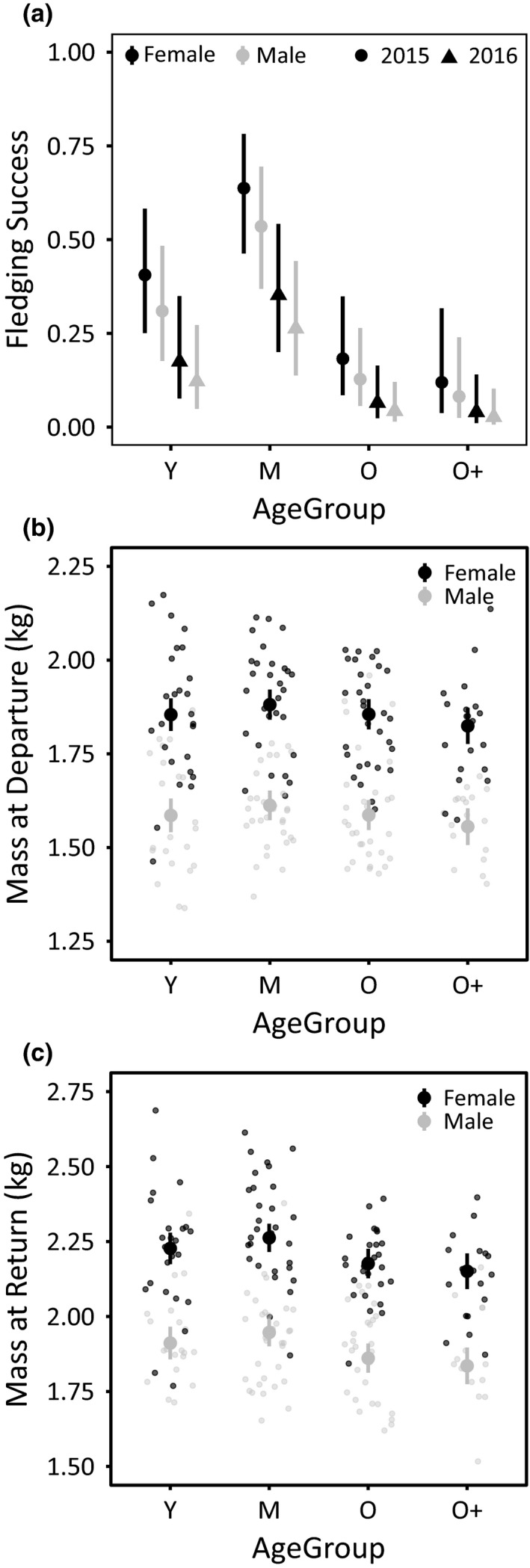
AgeGroup, Sex, and Breeding Season differences in (a) Fledging Success (the probability of raising an independent offspring, given a breeding attempt), and (b) AgeGroup and Sex differences in Mass at departure, and (c) Mass at return of a foraging absence in Nazca boobies. In all panels, large points are model‐predicted means ± 95% CI from generalized linear models with (a) binomial or (b/c) Gaussian errors (SI Results Section 24)

Foraging outcomes generally dictate reproductive success in pelagic seabirds (e.g., Gall et al., 2006; Regular et al., 2014). Noting that middle‐aged Nazca boobies have superior reproductive performance at all stages of the reproductive cycle (Tompkins & Anderson, 2019; Tompkins et al., 2017), we evaluated age‐related variation in foraging flight performance and food delivery to the nest. A similar middle‐age superiority in foraging outcomes would implicate food acquisition, including long‐distance flight, as a contributor to compromised reproduction in young and old breeders. We focused on incubating adults because food acquisition early in the breeding cycle has a large influence on reproductive success in this species *via* nutrition effects on egg laying (Clifford & Anderson, 2001a) and nest abandonment (Tompkins et al., 2017), especially in young and old birds. Age did not affect Mass Gain/hr or Absence Duration, and we found only limited evidence of decreasing Mass Gain in old age. The tagged group of birds (209 individuals) provides evidence of reproductive senescence: annual probabilities of raising an offspring to independence (“Fledging Success”) declined with age (Figure 4a, showing results from a *post hoc* analysis described in the SI Results Section 24), matching the pattern revealed by longitudinal studies following thousands of individuals (Tompkins & Anderson, 2019; Tompkins et al., 2017). Modest declines in foraging outcomes (here limited to Mass Gain) may accumulate throughout the breeding cycle, resulting in stronger senescence for traits, like offspring production, that integrate parental performance over many months. Although senescence in offspring production is common in seabirds (e.g., Crespin et al., 2006; Pardo et al., 2013; Tompkins et al., 2017), evidence for senescence in foraging performance has been mixed. Wandering albatrosses (*Diomedea exulans*) do not show senescence in trip duration (Froy et al., 2015), and thick‐billed murres (*Uria lomvia*) do not show senescence for diving behavior (Elliott et al., 2015). However, other studies have reported longer trip duration (Catry et al., 2006; Frankish et al., 2020) or lengthening trip distance (Lecomte et al., 2010) in old seabirds. It may be challenging to detect the effects of late‐life senescence on foraging parameters measured on the scale of a single foraging absence or foraging trip, particularly because such physiological declines may be modest in long‐lived pelagic seabirds (e.g., Angelier et al., 2007; Elliott et al., 2015; Lecomte et al., 2010). Incorporating traits that measure accumulated deficits in food acquisition over longer time periods (e.g., chick growth rates, changes in parental mass) may be instrumental in interpreting weak evidence for senescence in fine‐scale foraging behaviors, and in understanding the relationships between physiology, foraging, and reproductive outcomes.

Our data provide weak evidence for middle‐age superiority in Mass Gain, with Young, Old, and Oldest birds returning to the nest with smaller Mass Gains (negative β values) than Middle Age birds, but the 95% CI on AgeGroup coefficients included zero (although marginally so for Oldest birds; Old: *β* = −38.16 [95% CI: −94.91, 18.58]; Oldest: *β* = −53.90 [95% CI: −116.44, 8.64]. Controlling other predictors at their mean or baseline values (for factors), Old and Oldest birds gained 9.7% and 13.7% less mass, respectively, during foraging absences. Lower Mass Gains with age during a foraging absence are expected to reduce the body mass of Old and Oldest birds (relative to prime‐age adults) at their return to incubation. We tested this idea *post hoc* using mass measurements at deployment and return from tagged birds (Table S24). Although mass at departure did not vary by AgeGroup (Figure 4b), Old and Oldest birds returned from foraging with a lower body mass than Middle Age birds, consistent with the suggestive result for Mass Gain (Old: *β* = −0.09 [95% CI: −0.14, −0.03]; Oldest: *β* = −0.11 [95% CI: −0.18, −0.04]; Figure 4c). Older Nazca boobies returned from a foraging trip with less food to sustain themselves during the subsequent multi‐day incubation bout, providing insight into the smaller clutches (Tompkins and Anderson in review) and higher failure rate for females of advancing ages during the incubation period (Tompkins et al., 2017).

### 4.2 Age‐related variation in flight performance

Old and Oldest boobies flew more slowly than other age classes late in foraging absences (Figure 3), when stamina deficits may be increasingly important. We used Airspeed as a direct assay of flight performance and as the principal contributor to Groundspeed; indeed, parallel analyses of age effects on Groundspeed also showed late‐life declines during inbound flight (Tables S22 and S23; Figure S12). Faster Airspeed and Groundspeed will shorten commuting and searching time and improve the rate of food delivery to the nest to sustain the subsequent incubation bout. Faster Airspeed and Groundspeed will also shorten the incubation bout imposed on the mate, reducing the risk of clutch abandonment. Thus, one interpretation casts late‐life decline in Airspeed as evidence of constraint placed on the performance of old individuals by physiological senescence.

Foraging Nazca boobies dive for prey on average only 6.7 times per day; thus, much of the foraging effort comprises level flapping commuting flight (Zavalaga et al., 2012). To understand how effort expended in propulsion during this locomotion might influence the observed age effects on Airspeed, we used accelerometers positioned on the midline to estimate the cost of flapping flight (ODBA) and the production of thrust and lift indirectly *via* Wingbeat Frequency, wingbeat strength (Body Displacement), and the allocation of flight time to flapping versus gliding. Unexpectedly, this flight kinematics showed no age‐related variation during outbound or inbound flight (Table 3). Wingbeat Frequency, Flap–Glide Ratio, and Flapping ODBA had low within‐individual repeatabilities (*R* < 0.30; Table S21), so these may be relatively poor proxies for the airframe's performance and the physiological state of an individual. In contrast, Body Displacement was more consistent (*R* = 0.45 outbound and 0.65 inbound; Table S21), yet was also unrelated to age. The absence of age effects in these performance metrics, especially in old birds with negligible breeding success (Figure 4a), implies that the function of axial flight muscles and their connective tissues do not decline in older Nazca boobies. While muscle function does degrade in old age in several wild mammals (Hämäläinen et al., 2015; Hindle et al., 2009; Hindle et al., 2009), equivalent studies in two seabirds gave mixed results. The myonuclear domain, but not muscle diameter, of the pectoralis muscle shrinks with increasing age in thick‐billed murres (Elliott et al., 2015; Jimenez et al., 2019), but black‐legged kittiwakes (*Rissa tridactyla*) showed no change in musculature with age (Brown et al., 2019). Instead of a deficit in power production, stamina deficits of appendicular muscles that control the position and movement of flight surfaces during flapping (Pennycuick et al., 1988) remain as a possible physiological cause of the Airspeed decline in old age.

Although faster airspeed will reduce the time spent traveling and searching for prey, transport costs increase with flight speed and depend on wind (Liechti, 1995; Liechti et al., 1994), raising the possibility that the slower flight speeds of old birds reflect adaptive adjustments to increase flight efficiency, not constraint imposed by physiological decline. Optimal airspeed is predicted to increase in headwinds or cross winds (Hedenström & Alerstam, 1995; Liechti, 2006), tracking environmental effects on the maximum range speed (minimizing mechanical energy expenditure per unit of distance travelled), and aerodynamics may also be affected by turbulence (Reynolds et al., 2014). Theoretic and empirical studies suggest that birds can modulate Airspeed during goal‐directed flight in reaction to wind conditions to minimize transport costs or time (Collins et al., 2020; Pennycuick, 1978; Shamoun‐Baranes et al., 2007). Tailwind component (TWC) was incorporated into our models of Airspeed, but cross wind strength and/or fine‐scale wind features were not controlled and may vary between age classes (as for TWC, Figure S7) if, for example, birds of different ages differ in departure time, heading, or flight height. Thus, although we find clear evidence that Airspeed changes across the life span, being consistently lower in older birds, we cannot distinguish constraints imposed by physiological declines from adaptive shifts to minimize energetic costs in a variable environment.

### 4.3 Sex effects in foraging and flight performance

Female Nazca boobies in this study averaged 17% heavier than males and 3.5% longer in wing chord, although overlap in the size distributions is substantial (Figure S11). This size difference might translate into foraging and flight differences between the sexes, either for adaptive reasons (e.g., Andersson & Norberg, 1981) or as the result of biomechanical consequences (Pennycuick, 1989). Indeed, females gained 17.9% more mass over a foraging absence than males did (Figure 2a), corresponding well with the 17.0% difference in baseline body mass (Figure S11a) and matching foraging outcomes during chick rearing (Anderson & Ricklefs, 1992). Thus, the larger sex, presumably facing a larger daily energy requirement (in absolute terms) during the coming incubation bout, returned with a larger prey load to sustain themselves. Larger mass gains by foraging females were accomplished without extending their time spent foraging, relative to the Absence Durations of males (Table 3), but did not result in higher Mass Gain/hr (Table 3). A high degree of unexplained variation in models of Mass Gain/hr (*R* ^2^ = 0.14) may explain this apparent contradiction.

Males and females, regardless of age, expressed sex‐specific combinations of accelerometer‐derived flight components, resulting in relatively similar Airspeeds. Sex was a top predictor explaining variation in Wingbeat Frequency, Flap–Glide Ratio, and Flapping ODBA. Males flapped faster than females but spent a larger fraction of time gliding than females did. Males had higher Flapping ODBA (a proxy for energy expenditure; reviewed in Elliott, 2016) per second of flapping in a flapping bout than females. This Flapping ODBA result is related to males' faster flapping and greater Body Displacement (though 95% CI on Sex spanned zero), because Flapping ODBA is calculated from the dynamic acceleration of the three axes (SI Methods Section 2). These contrasting combinations of flight traits observed in Nazca boobies match expectations from overall body size effects on intermittent flight: in flap‐bounding woodpeckers and songbirds, wingbeat frequency decreases with mass in tandem with relative flapping (vs. gliding/bounding) increasing with mass (Tobalske, 2001). Our study marks the first detection of these sex‐specific flapping syndromes in seabirds and remains to be tested in other species with size/wing loading differences that also flap–glide.

### 4.4 Environmental effects on foraging outcomes and flight performance

Tropical seabird populations typically suffer negative outcomes under El Niño conditions, including delayed or reduced breeding participation (Anderson, 1989; Cubaynes et al., 2010), poor hatching and/or fledging success (Ancona et al., 2011; Champagnon et al., 2018), and increased adult mortality (Boersma, 1998). Our study included the extreme 2015–2016 El Niño (Santoso et al., 2017) and the following breeding season (during a mild La Niña). Foraging Nazca boobies gained mass faster (Mass Gain/hr) during the 2015 El Niño, driven by much shorter foraging absences in 2015 than during the following year (Figure 1). During El Niño events, sea surface temperature rises and primary productivity falls in the Eastern Tropical Pacific (ETP; Feldman et al., 1984; Pennington et al., 2006). These oceanographic changes typically peak around December–February (Santoso et al., 2017; Wang & Fiedler, 2006), and large positive sea surface temperature anomalies were already apparent in the ETP in November 2015–January 2016, when flight performance and foraging outcomes were collected from incubating Nazca boobies (average Nov–Jan SSTA = 2.64°C in the NINO3 region; SI Results Section 25). Oceanographic conditions during the 2016 breeding season were markedly different: sea surface temperature averaged 2.7°C cooler, and the chlorophyll *a* concentration averaged 0.23 mg/m^3^ higher in 2016 in the Nazca booby foraging area (Zavalaga et al., 2012). Although our foraging data span only 2 years, apparently favorable foraging conditions during the onset of El Niño align with positive effects of El Niño on Nazca booby egg‐laying performance that were evaluated using two decades of observations (Tompkins & Anderson, 2021). Clearly, simple predictions based on patterns of primary productivity (El Niño bad, La Niña good) are inadequate for this top predator.

Transient improvements in diet early in an El Niño may drive improvements in foraging performance (this study) and, hence, clutch size and—for young birds—breeding date and breeding participation (Tompkins & Anderson, 2021). However, diet characteristics during the 1986–87 El Niño indicated a degraded, not improved, prey base (Anderson, 1989). Thus, available evidence from prey species, while incomplete, does not explain positive effects of El Niño on foraging outcomes and egg‐laying traits, although it may contribute to negative effects of El Niño on offspring survival to independence and juvenile survival, traits expressed late in the breeding cycle (Anderson, 1989; Champagnon et al., 2018; Tompkins & Anderson, 2021). Instead, the prey base in its own increasingly challenging environment early in an El Niño could, paradoxically, become more available to these aerial predators. Nazca boobies plunge dive for small pelagic fishes close to the sea surface (Anderson & Ricklefs, 1992), accessing prey that have been pushed upward by subsurface predators like yellowfin tuna (*Thunnus albacares*) or dolphins (particularly *Stenella spp*.; Au & Pitman, 1986). We follow Tompkins (2018) in speculating that opportunities for this “facilitated foraging", and therefore prey availability, increase during the onset of El Niño for incubating Nazca boobies. During El Niño, the Equatorial Front weakens or disappears and the Equatorial Cold Tongue warms (Spear et al., 2001; Wang & Fiedler, 2006), weakening or eliminating differences between the tropical waters where yellowfin tuna are most abundant (Hu et al., 2018) and tuna–dolphin–seabird feeding assemblages occur most frequently (Au & Pitman, 1986; Ballance et al., 2006) and the equatorial waters where Nazca boobies forage. Tuna may follow this oceanographic homogenization into the Nazca booby foraging radius, and/or changes to the water column may better suit the pursuit of schooling fishes by subsurface predators and the formation of tuna–dolphin–seabird foraging groups (Spear et al., 2001). Under this scenario, improved prey availability during incubation degrades by the end of chick rearing (*via* prey being eaten and/or negatively affected by El Niño‐forced reductions in primary productivity), explaining the typically poor eventual breeding success during El Niños (Champagnon et al., 2018).

## CONFLICT OF INTEREST

The authors declare no conflict of interest.

## AUTHOR CONTRIBUTIONS

**Jennifer L. Howard:** Conceptualization (equal); data curation (lead); formal analysis (lead); funding acquisition (supporting); investigation (equal); methodology (equal); resources (equal); software (equal); supervision (supporting); validation (lead); visualization (lead); writing‐original draft (lead); writing‐review & editing (lead). **Emily M. Tompkins:** Conceptualization (supporting); formal analysis (equal); supervision (equal); validation (equal); writing‐original draft (supporting); writing‐review & editing (equal). **David J. Anderson:** Conceptualization (equal); funding acquisition (lead); investigation (equal); methodology (equal); project administration (equal); supervision (equal); writing‐review & editing (equal).

## Supporting information

Supplementary Material

## Data Availability

Data files are available from the WakeSpace database: https://wakespace.lib.wfu.edu/handle/10339/97990.
