# Supplementary material for: Effects of age, sex, and ENSO phase on foraging and flight performance in Nazca boobies
Source: Ecol Evol. 2021 Mar 10;11(9):4084–100. doi: 10.1002/ece3.7308 (PMC8093656; doi:10.1002/ece3.7308)
Supplement: Supplementary file 1 — Supplementary Material [file ECE3-11-4084-s001.docx]

[SUPPLEMENTARY METHODS 4](#_Toc61595596)

[1. Study Site and Tagging Methods 4](#_Toc61595597)

[Table S1. 5](#_Toc61595598)

[2. Calculation of Accelerometer and GPS-Derived Flight Components 5](#_Toc61595599)

[Figure S1. 7](#_Toc61595600)

[Figure S2 9](#_Toc61595601)

[Figure S3. 10](#_Toc61595602)

[Table S2. 11](#_Toc61595603)

[Table S3. 12](#_Toc61595604)

[3. Wingbeat Frequency Validation 13](#_Toc61595605)

[4. 2016 Accelerometer Calibration 14](#_Toc61595606)

[Figure S4. 15](#_Toc61595607)

[Figure S5. 17](#_Toc61595608)

[5. Wind Variation and Calculating Tailwind Component 18](#_Toc61595609)

[Figure S6. 19](#_Toc61595610)

[Figure S7. 20](#_Toc61595611)

[Figure S8 21](#_Toc61595612)

[6. Correction Made to Initial Body Mass (MassPre) 22](#_Toc61595613)

[Figure S9. 23](#_Toc61595614)

[7. Estimating Wing Loading 23](#_Toc61595615)

[Figure S10. 25](#_Toc61595616)

[8. Patterns of Covariation among Predictor Variables 26](#_Toc61595617)

[Table S4. 26](#_Toc61595618)

[8.1 Age and Environment 27](#_Toc61595619)

[8.2 Age and Wing Loading 27](#_Toc61595620)

[8.3 Body Size and Sex 27](#_Toc61595621)

[Figure S11. 28](#_Toc61595622)

[SUPPLEMENTARY RESULTS 29](#_Toc61595623)

[9. Effects of Incubation Days on Response Variables 29](#_Toc61595624)

[Table S5 31](#_Toc61595625)

[Table S6 32](#_Toc61595626)

[Table S7 33](#_Toc61595627)

[Table S8 34](#_Toc61595628)

[Table S9 35](#_Toc61595629)

[10. Complete Model Results: Mass Gain Rate 36](#_Toc61595630)

[Table S10 36](#_Toc61595631)

[11. Complete Model Results: Mass Gain 37](#_Toc61595632)

[Table S11 37](#_Toc61595633)

[12. Complete Model Results: Absence Duration 38](#_Toc61595634)

[Table S12 38](#_Toc61595635)

[13. Complete Model Results: Airspeed 39](#_Toc61595636)

[Table S13 39](#_Toc61595637)

[14. Complete Model Results: Wingbeat Frequency 41](#_Toc61595638)

[Table S14 41](#_Toc61595639)

[15. Complete Model Results: Flap-Glide Ratio 43](#_Toc61595640)

[Table S15. 43](#_Toc61595641)

[16. Complete Model Results: Body Displacement 45](#_Toc61595642)

[Table S16 45](#_Toc61595643)

[17. Complete Model Results: Flapping ODBA 47](#_Toc61595644)

[Table S17. 47](#_Toc61595645)

[18. Date and TWC Coefficient Estimates 49](#_Toc61595646)

[Table S18 49](#_Toc61595647)

[19. Summarized Flight Performance by Sex 50](#_Toc61595648)

[Table S19. 50](#_Toc61595649)

[20. Airspeed and Accelerometer-derived Flight Components 51](#_Toc61595650)

[Table S20. 52](#_Toc61595651)

[Table S21. 53](#_Toc61595652)

[21. Airspeed and Groundspeed 54](#_Toc61595653)

[22. Complete Model Results: Groundspeed 55](#_Toc61595654)

[Table S22. 55](#_Toc61595655)

[23. Coefficient Estimates: Groundspeed 57](#_Toc61595656)

[Table S23. 57](#_Toc61595657)

[Figure S12. 58](#_Toc61595658)

[24. Effects of AgeGroup and Sex on Body Mass and Fledging Success 59](#_Toc61595659)

[Table S24 60](#_Toc61595660)

[25. Environmental Variation in the Nazca Booby Foraging Area 61](#_Toc61595661)

[REFERENCES 61](#_Toc61595662)

# SUPPLEMENTARY METHODS

## 1. Study Site and Tagging Methods

Tagged birds were drawn from the pool of available incubating adults in October- December of the 2015 and 2016 breeding seasons. The indifference of the birds to our presence facilitates capture (always by hand) and data collection. Loggers were deployed on the second or third day of an incubation bout for a focal individual (most incubation bouts lasted 2-5 days during the study), and at nests established ≥7 days earlier. Daily nest monitoring in 2015 and 2016 provided egg laying dates and incubation schedules. To reduce temporal environmental noise, loggers were deployed on date-matched triplets or quadruplets: a given deployment comprised a same-sex cohort with one member of each age group (occasionally lacking an “Oldest” member).

Two models of GPS loggers (Mobile Action Technology, Taiwan) were used during the study, deployed evenly across the sexes and age classes (Table S1). The smaller GT-120 loggers were attached under the tail to minimize drag during plunge dives; the larger GT-600 loggers were attached above the rectrices to avoid these loggers hitting rocks when the birds were on land. During aerial plunge dives, boobies press their feet together against the ventral bases of the rectrices, protecting ventrally-mounted loggers (Anderson & Ricklefs, 1987). GPS loggers were enclosed in two latex condoms to protect them from water. Birds were captured at the nest to attach the loggers with Tesa® waterproof tape (product 4651; Tesa, Charlotte, NC, USA). We attached the accelerometers to feathers on the lower back, 5 cm above the uropygial gland and along the spine, for consistency of pitch (body angle) across individuals during level flapping flight. The capture and logger attachment process lasted < 8 minutes and birds resumed normal activity almost immediately. Loggers were typically retrieved within 3 hours of the foraging absence’s completion. Five individuals were tagged during both breeding seasons; for those individuals, data from only one season were used in analyses. All research adhered to the permit and regulations of the Wake Forest University Institutional Animal Care and Use Committee (protocol A14-147).

Table S1. Distribution of GPS loggers (Mobile Action Technology, Taiwan) deployed on Nazca boobies in 2015 and 2016 by logger model, Sex, and AgeGroup. See Table S2 for the sample size of birds from which we obtained useable data.

| **Breeding Season** | **GPS Logger** | **Sex** | **AgeGroup** | | | |
| --- | --- | --- | --- | --- | --- | --- |
|  |  |  | **Young**  **(4-7 yrs)** | **Middle Age (11-14 yrs)** | **Old (18-20 yrs)** | **Oldest**  **(21-24 yrs)** |
| **2015** | **GT-120** | Male | 7 | 7 | 7 | 6 |
|  |  | Female | 3 | 3 | 3 | 3 |
|  | **GT-600** | Male | 15 | 13 | 15 | 8 |
|  |  | Female | 13 | 15 | 15 | 7 |
| **2016** | **GT-120** | Male | - | - | - | - |
|  |  | Female | - | - | - | 1 |
|  | **GT-600** | Male | 9 | 15 | 12 | 9 |
|  |  | Female | 10 | 17 | 15 | 13 |
| **All** | **All** | Male | 29 | 35 | 34 | 20 |
|  |  | Female | 28 | 35 | 33 | 27 |
|  |  | **Total** | **57** | **70** | **67** | **47** |

## 2. Calculation of Accelerometer and GPS-Derived Flight Components

We examined age effects on four variables calculated from accelerometer data: Wingbeat Frequency, Flap-Glide Ratio, dorso-ventral Body Displacement, and Flapping Overall Dynamic Body Acceleration (ODBA). Acceleration data were recorded on three axes: anterior-posterior (x-axis), lateral (y-axis), and dorso-ventral (z-axis). We focused on acceleration measurements from the dorso-ventral axis (the z-axis), which captures flapping. A running mean was applied over a two second window across the raw acceleration data to calculate static acceleration, the component associated with gravity (Shepard et al., 2008). Dynamic acceleration, the animal’s contribution, was calculated by subtracting the static acceleration from the raw acceleration values. Flapping was identified by regular oscillations in amplitude in the time series of dynamic Z (Ropert-Coudert et al., 2009; Fig. S1).

To calculate accelerometer-derived response variables, individual flaps and bouts of these flaps must be distinguished from other activities (principally gliding in this case). Individual flaps were isolated by locating local maxima/minima (“peaks”) in amplitude within the time series of dynamic Z using the “findpeaks” function in R package *pracma* (v. 2.1.5, Borchers, 2018; Fig. S1, Step 1). When all local maxima/minima were located, peaks in dynamic Z (“flaps”) had to be discriminated from those associated with non-flapping activity. This discrimination process was done in four steps (Fig. S1).


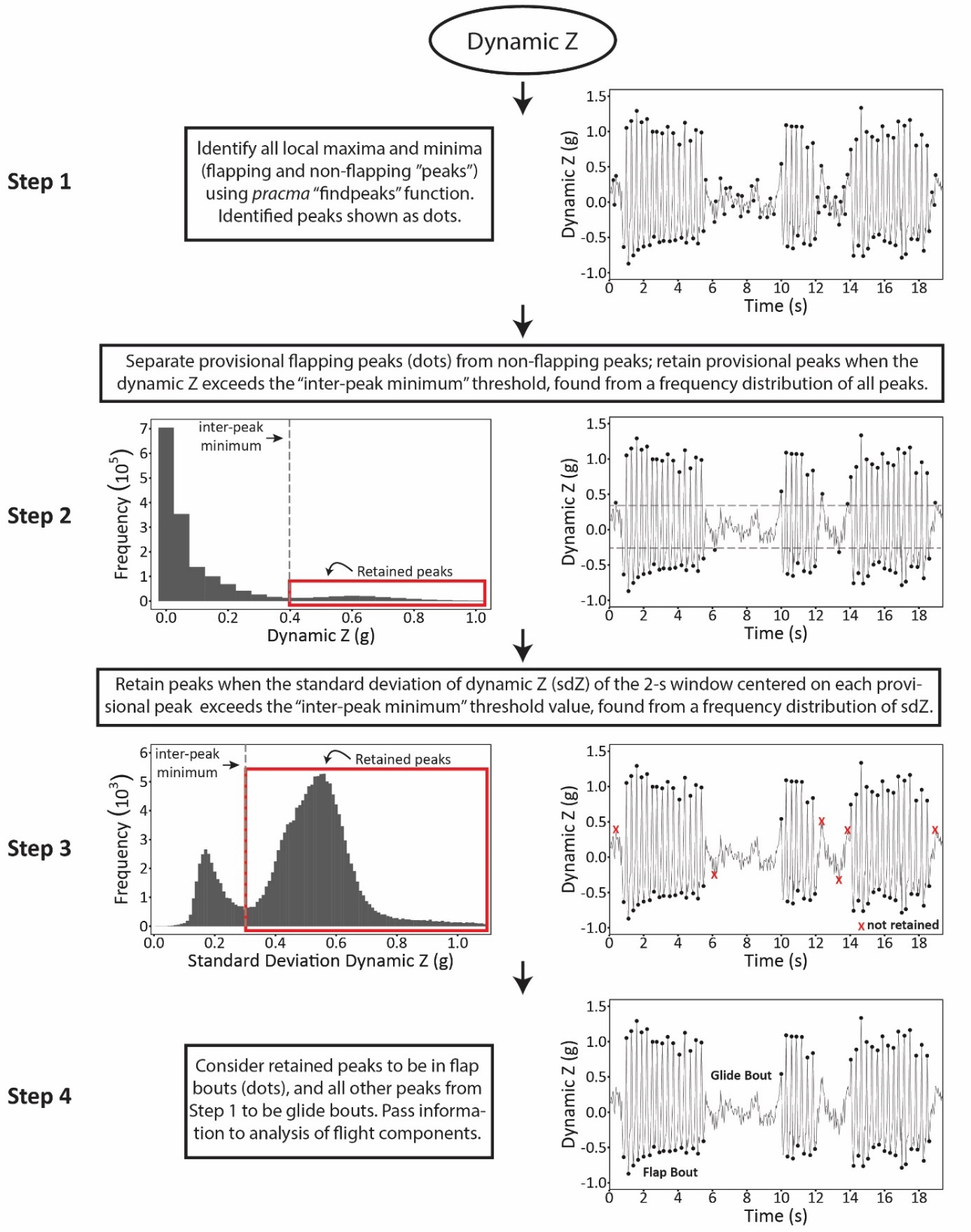


Figure S1. Flow chart illustrating how flapping bouts and gliding bouts were identified within the dynamic Z time series data for 20 seconds of typical travel by a representative bird. Inter-peak minimum thresholds were identified separately for local maxima/minima for each bird.

Values of dynamic Z at the local peaks corresponding to true flaps (black dots in Fig. S1) exceed those at local peaks corresponding to non-flapping activity. Because Nazca boobies use a flap-glide flight mode, the frequency distribution of local peak dynamic Z for a given individual/deployment was bimodal, with maxima at 0g and 1g (Fig. S1, Step 2). Local peak dynamic Z values around 0g represent data collected when the bird was not moving (e.g., sitting on the nest), while values around 1g are flaps. The value of local peak dynamic Z occurring at the inter-peak minimum frequency (the trough between the two maxima of the bimodal frequency distribution) was used as a threshold to identify local peaks in dynamic Z corresponding with flapping (peak dynamic Z > the threshold for local maxima). Threshold values used to isolate flapping peaks from non-flapping peaks in dynamic Z were individual-specific and were calculated separately for local maxima versus local minima.

The subset of local peaks in dynamic Z selected using threshold values of local peak dynamic Z (described above) still included peaks labeled as false-positive flaps (Fig. S1, Step 2). Thus, we used the standard deviation of dynamic Z (sdZ) calculated from a moving 2-second window (Collins et al., 2015) to further discriminate flaps from false positives (Fig. S1, Step 3). A relatively large sdZ corresponds to the large amplitude changes in dynamic Z during flaps; a relatively small sdZ corresponds to the smaller variation in dynamic Z observed during gliding or other non-flapping activity. As above, the interpeak minimum frequency of sdZ was used as a threshold to discriminate flaps from false positives (Fig. S1, Step 3); peaks in dynamic Z with a sdZ lower than the threshold were not labelled as “flaps” in subsequent calculations of our flight performance response variables (Fig. S1, Step 4). This discrimination process was done separately for local maxima (displacement up during a bird’s downstroke) versus local minima (displacement down during a bird’s upstroke) in each time series (Fig. S2).

**
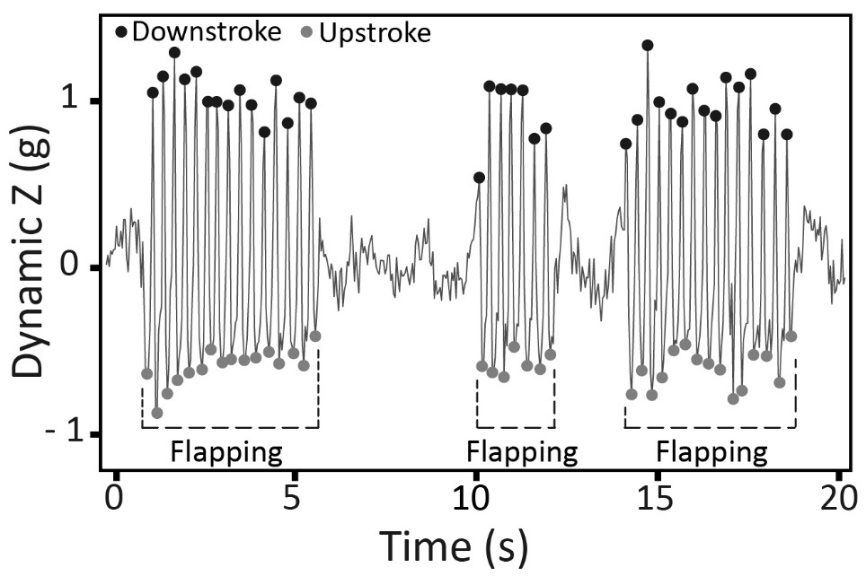
**

Figure S2**.** Flapping and gliding bouts detected in 20 seconds of a bird’s dorso-ventral acceleration (Dynamic Z). Maxima and minima during flapping bouts correspond to maximum forces developed during downstroke (black) and upstroke (gray), respectively. Dashed lines indicate flapping bouts within this 20 second period; flapping bouts are separated by gliding.

Wingbeat Frequency was related to the number of flaps in a bout (Fig. S3), so we minimized bias while retaining sample size by removing flapping bouts with fewer than three individual flaps. To isolate level flapping flight, other flight modes (take-off, landing, or diving) during outbound and inbound periods were removed by filtering periods of extreme values in dynamic Z (>2.0 g) or changes in body pitch >25°. Pitch, a bird’s departure from a horizontal body posture, was calculated from static acceleration of the three axes (S_x_, S_y_, S_z_) from equation 3 of Chimienti et al. (2016):

pitch = $\tan^{-1} \left( \frac{S_{x}}{\sqrt{S_{y}^{2}+S_{z}^{2}}} \right)* \frac{180}{\pi}$ (1)

**
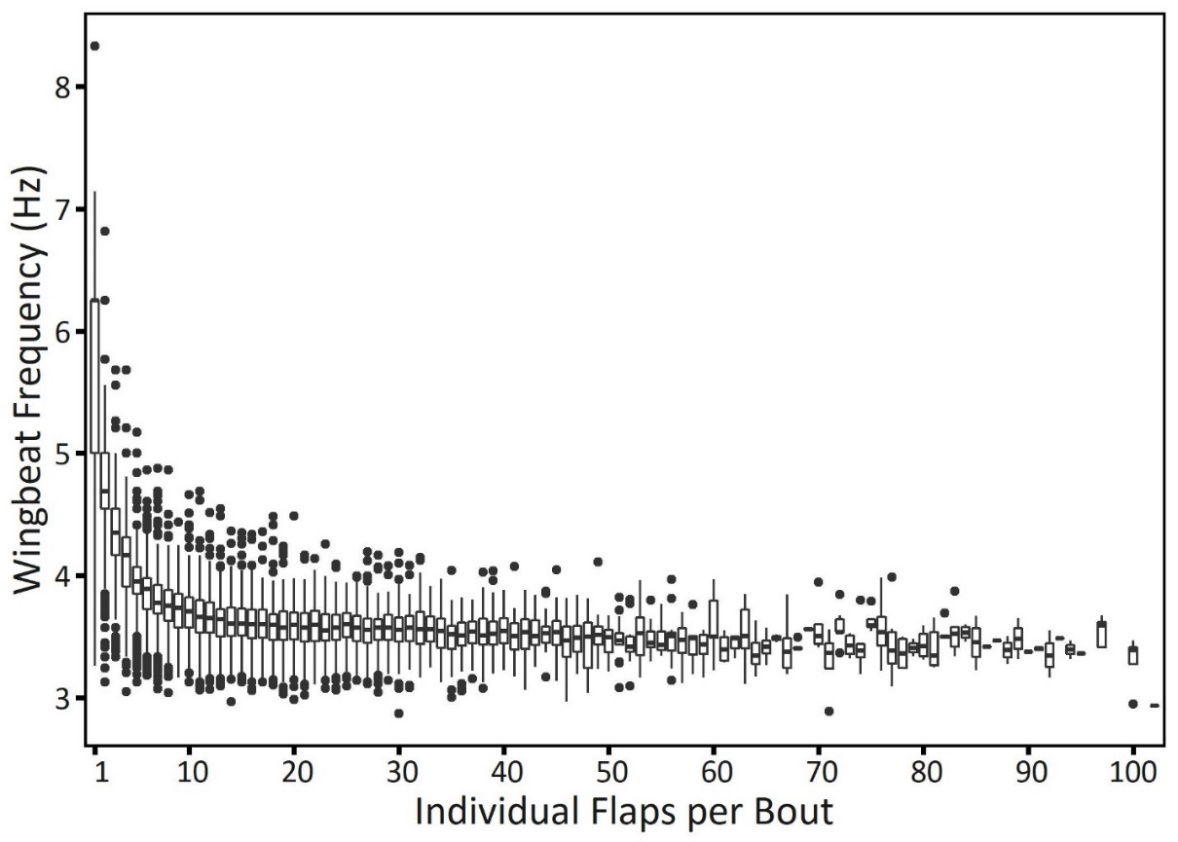
**

Figure S3. Wingbeat Frequency varied by the number of individual flaps in each flapping bout. Data come from all birds included in outbound models (n = 209). The lower and upper edges of each box correspond to the first and third quartiles; whiskers extend from the edge to the largest value no further than 1.5x the interquartile range. Individual points are outliers.

From the values of dynamic Z at the local maxima/minima corresponding to flaps we calculated Body Displacement (the change in dynamic Z from one maximum to the following minimum), Flap-Glide Ratio, Wingbeat Frequency, and Flapping ODBA (ODBA per second of flapping, used as a proxy for energy expenditure during a flap bout). ODBA was calculated from dynamic acceleration of the three axes (D_x_, D_y_, D_z_) following Patterson, Gilchrist, Chivers, Hatch, & Elliott (2018):

ODBA = $\left| D_{x} \right|+\left| D_{y} \right|+\left| D_{z} \right|$ (2)

A typical 30-minute time series involved >200 flapping bouts; a mean value for each flight component per 30-minute period was used as a response variable in statistical models to match the scale of the response variables with that of the covariates (c.f., we had only one mass for each outbound period and one mass for each inbound). While recognizing that age-related physiological deficits may constrain the upper limit of a bird’s performance more than the mean (Elliott et al., 2014), we chose to model age effects on average accelerometer-derived flight components because average changes in performance with age should correspond best to downstream fitness consequences. Because some accelerometers failed or were lost during trips, we obtained usable flight component data from 209 outbound individuals (Table S2; 2015: n = 132; 2016: n = 77) and 194 inbound individuals (2015: n = 125; 2016: n = 69). Pairwise comparisons between accelerometer-derived flight components are reported in Table S3. Pearson’s correlation tests were used to measure their association with each other.

Table S2. Distribution of Nazca boobies by Sex and AgeGroup that provided data during the outbound period. The inbound period had a smaller sample size (n = 194).

|  | Young | Middle Age | Old | Oldest | Total |
| --- | --- | --- | --- | --- | --- |
| Males | 22 | 32 | 31 | 18 | 103 |
| Females | 26 | 30 | 30 | 20 | 106 |
| Total | **48** | **62** | **61** | **38** | **209** |

Table S3. Pearson’s correlation coefficients comparing pairs of accelerometer-derived flight components, separated by flapping period. Statistically significant results (p < 0.05) are in bold. Data from males and females are treated separately.

| **Flapping Period** | **Component 1** | **Component 2** | **Males** | **Females** |
| --- | --- | --- | --- | --- |
| Outbound | Wingbeat Frequency | Body Displacement | r = 0.15, df = 101, p = 0.14 | **r = 0.26, df = 104, p < 0.01** |
|  | Wingbeat Frequency | Flapping ODBA | **r = 0.22, df = 101, p = 0.02** | **r = 0.32, df = 104, p < 0.01** |
|  | Wingbeat Frequency | Flap-Glide Ratio | **r = -0.33, df = 101, p < 0.01** | **r = -0.36, df = 104, p < 0.01** |
|  | Body Displacement | Flapping ODBA | **r = 0.56, df = 101, p < 0.01** | **r = 0.64, df = 104, p < 0.01** |
|  | Body Displacement | Flap-Glide Ratio | **r = -0.28, df = 101, p < 0.01** | **r = -0.23, df = 104, p = 0.02** |
|  | Flapping ODBA | Flap-Glide Ratio | **r = -0.26, df = 101, p < 0.01** | **r = -0.30, df = 104, p < 0.01** |
| Inbound | Wingbeat Frequency | Body Displacement | r = -0.11, df = 96, p = 0.30 | r = -0.19, df = 94, p = 0.06 |
|  | Wingbeat Frequency | Flapping ODBA | r = -0.10, df = 96, p = 0.33 | r = -0.05, df = 94, p = 0.61 |
|  | Wingbeat Frequency | Flap-Glide Ratio | r = -0.05, df = 96, p = 0.66 | r = -0.04, df = 94, p = 0.67 |
|  | Body Displacement | Flapping ODBA | **r = 0.56, df = 96, p < 0.01** | **r = 0.59, df = 94, p < 0.01** |
|  | Body Displacement | Flap-Glide Ratio | **r = -0.44, df = 96, p < 0.01** | **r = -0.32, df = 94, p < 0.01** |
|  | Flapping ODBA | Flap-Glide Ratio | **r = -0.35, df = 96, p < 0.01** | **r = -0.25, df = 94, p = 0.02** |

Groundspeed was calculated from each pair of adjacent GPS positions across 3 minute intervals, for outbound and inbound periods (each 30 minutes in duration, during commuting flight), and provided the speed relative to the ground (Kogure, Sato, Watanuki, Wanless, & Daunt, 2016). Measures of Groundspeed (G), a bird’s bearing (departure angle from colony [ɵ], from the GPS logger), wind speed (W), and wind direction (ɷ) were used to calculate Airspeed following equation 6 from Shamoun-Baranes, van Loon, Liechti, & Bouten (2007):

Airspeed = $\sqrt{({(\left( G* \sinɵ \right)-(W* \sinɷ))}^{2}+{(\left( G* \cosɵ \right)-(W* \cosɷ))}^{2})}$ (3)

Mean values of Groundspeed and Airspeed for each individual’s outbound and inbound period were used as the response variables in our analyses.

The Env-DATA annotation system in Movebank (www.movebank.org) was used to annotate each GPS position with a 10-m-above-ground wind vector from the European Centre for Medium-Range Weather Forecasts (ECMWF) at 3-hour temporal and 0.75-degree spatial resolution (Dodge et al., 2013). We removed values of Airspeed and Groundspeed if the bird was not in level flapping flight (verified visually in the accelerometer and GPS time series for the respective outbound and inbound 30-minute periods). As described above, most removals involved abrupt large changes in a bird’s pitch or dynamic Z (indicating landing on the water or taking off).

## 3. Wingbeat Frequency Validation

We validated our accelerometer-derived measurement of Wingbeat Frequency using video recordings of Nazca boobies approaching the colony from the sea (mean Wingbeat Frequency ± SD: 3.60 ± 0.25 Hz, n = 100, unknown sex and age), which aligned with our model-predicted values from accelerometers in this study (inbound Wingbeat Frequency: males = 3.83 Hz [95% CI: 3.80, 3.86]; females = 3.73 Hz [95% CI: 3.71, 3.76]).

## 4. 2016 Accelerometer Calibration

Preliminary examination of the response variables revealed an unexpected bimodal distribution for Flapping ODBA and Body Displacement (Fig. S4), suggesting that certain accelerometers reported Z acceleration incorrectly. Technosmart (Rome, Italy), the accelerometer manufacturer, and JLH isolated the problem to loggers produced in 2016 (data from loggers produced in 2015 were not affected; Fig. S4). Technosmart (Valeria Jennings, personal communication) indicated that the smaller Z acceleration values recorded in the 2016 loggers resembled a previously discovered issue (with a different batch of loggers): certain faulty accelerometers reported 1 unit of Z acceleration as 0.6 units of Z acceleration. Technosmart declined to examine the affected loggers, but proposed the following equation to convert the 2016 logger raw Z acceleration to the correct values:

corrected raw Z acceleration = stretched Z + offset (4)

in which stretched Z = raw Z acceleration/0.60 and offset = mean raw Z acceleration - mean stretched Z.

**
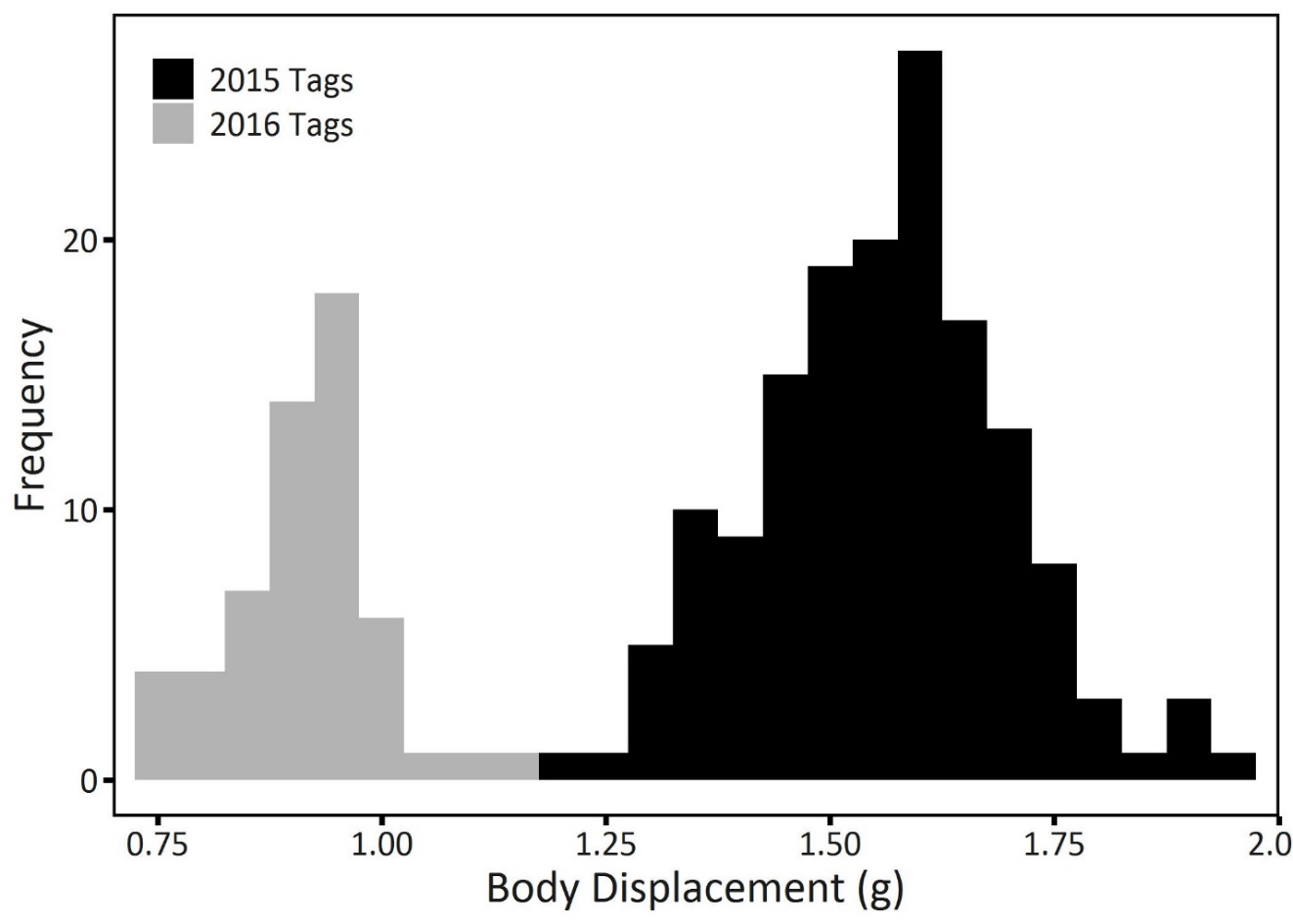
**

Figure S4. Non-overlapping frequency distributions of Body Displacement values (g) before correcting the Z acceleration of loggers manufactured in 2016.

We designed an analysis to confirm this conversion between raw Z acceleration recorded by loggers of 2015 vs. 2016 manufacture years (2015 vs. 2016 “logger groups”). We used the raw Z acceleration data from 2015 and 2016 logger groups attached to Nazca boobies and we assumed that, over the population of boobies contributing data to each logger group (2015 or 2016), the distribution of raw Z acceleration values during level flapping flight should be identical, if the 2016 loggers had not malfunctioned. We sampled 100 raw Z acceleration values from one flapping bout for each individual and pooled these values by logger group (2015 raw Z vs. 2016 raw Z). Then we described the relationship between the quantiles of the 2015 logger raw Z acceleration values and the quantiles of the 2016 logger raw Z acceleration values (0.05 - 0.95 quantiles) using a simple linear regression model.

We assumed that the distribution of raw Z acceleration values for the population of flapping birds with 2015 loggers should be identical to that of flapping birds equipped with 2016 loggers if the 2016 loggers had recorded Z acceleration correctly. Under this assumption, the linear regression model provides an equation to translate the values from 2016 loggers into 2015 logger raw Z acceleration values. Supporting this assumption, we have no reason to think that Body Displacement during a flap (which controls the range of Z acceleration) would be different, on average, between birds tagged with an accelerometer from one logger group over the other: the age structure and the sex ratio of sampled individuals was similar between logger groups, and the wind direction/speed was similar in the two seasons (Fig. S6, S8). Data were used from the outbound period only to take advantage of the larger sample size (209 birds) within the logger groups.

The quantiles from logger group-specific distributions of raw Z acceleration are shown in Fig. S5. Because this was a logger-related phenomenon, we analyzed data from the two sexes together and data from both seasons together (for 2015 loggers, which were deployed in the 2015 and 2016 breeding seasons). The linear regression of 2015 logger group quantiles on 2016 logger group quantiles revealed that a 1-unit change in Z from the 2015 loggers corresponds to a 0.62 (SE = 0.009) unit change in Z from the 2016 loggers. Thus, our analysis suggested that the issue affecting 2016 loggers is similar to that experienced by Technosmart with a previous group of loggers. The final equation used to correct 2016 logger raw acceleration (based on our linear regression) was: corrected raw Z acceleration = (original raw Z acceleration/0.62) - (0.35/0.62).


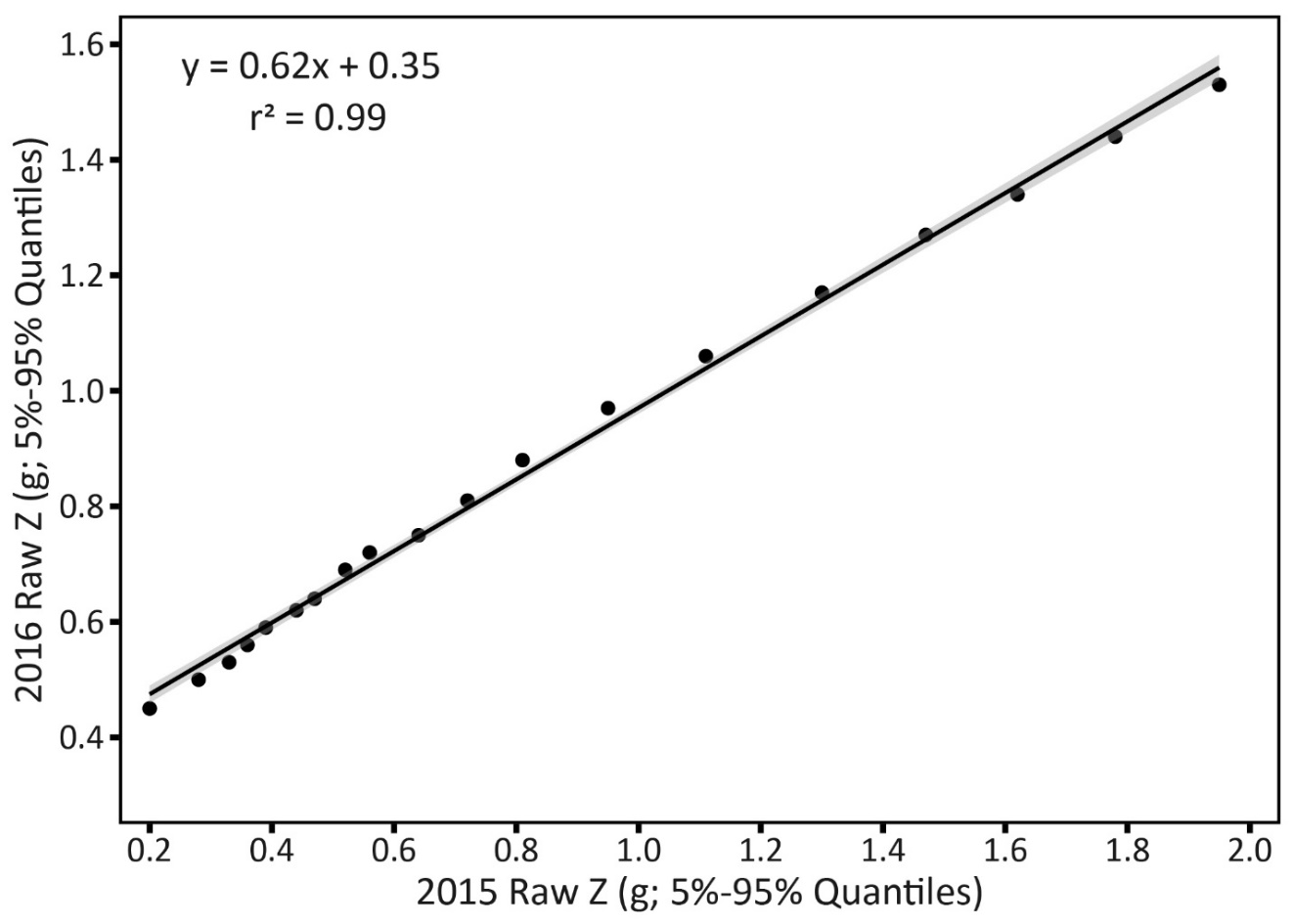


Figure S5. Linear regression comparing the quantiles of the distribution of raw Z acceleration during flapping bouts for loggers manufactured in 2015 vs. 2016. The black line (± 95% CI) is the prediction from a linear regression of 2015 raw Z quantiles on 2016 raw Z quantiles.

## 5. Wind Variation and Calculating Tailwind Component

Tailwind Component (“TWC”) captured wind speed relative to the bird’s direction of travel (negative values indicate a headwind and positive ones a tailwind). TWC was included as a predictor of flight component response variables because wind can affect flight behavior and airspeed (Kogure et al., 2016; Safi et al., 2013), influencing the cost of flight (Ballance, 1995). Wind came predominantly from the south-southeast (Fig. S6, 166 ± 21 degrees) during the two breeding seasons, with a minor difference between seasons (2015: 159 ± 16 degrees; 2016: 174 ± 23 degrees). TWC was a continuous variable, calculated as the product of the cosine of the absolute difference in the angle between the bird’s flight path and wind direction and wind speed following equation 2 from Kogure et al. (2016), and matching “wind support” from Safi et al. (2013). TWC is a measure of the support (+) or drag (-) that a bird receives from the wind during flight. TWC was missing for 5% (n = 10) of outbound trips and 11% of inbound trips (n = 21) due to GPS failure. To avoid discarding the flight parameters for trips with missing values, we replaced missing TWC with a mean TWC. TWC was correlated with departure hour during the outbound (Kendall τ_b_ = 0.22, p < 0.01), so the departure hour-specific mean TWC replaced missing values during the outbound. TWC during the inbound varied by AgeGroup (Fig. S7, Kruskal-Wallis, H = 11.66, df = 3, p < 0.01), so the AgeGroup-specific mean TWC replaced missing values during the inbound, allowing us to use the full sample size of accelerometer-derived flight parameters. As a *post hoc* analysis to assess a potential source of seasonal effects, we calculated cross wind, the speed of the wind vector perpendicular to the travel direction of the bird, following Safi et al. (2013). Cross wind varied more between birds’ outbound and inbound period, overall being more negative (blowing from the bird’s right) on the outbound and more positive on the inbound (blowing from the bird’s left; Fig. S8).


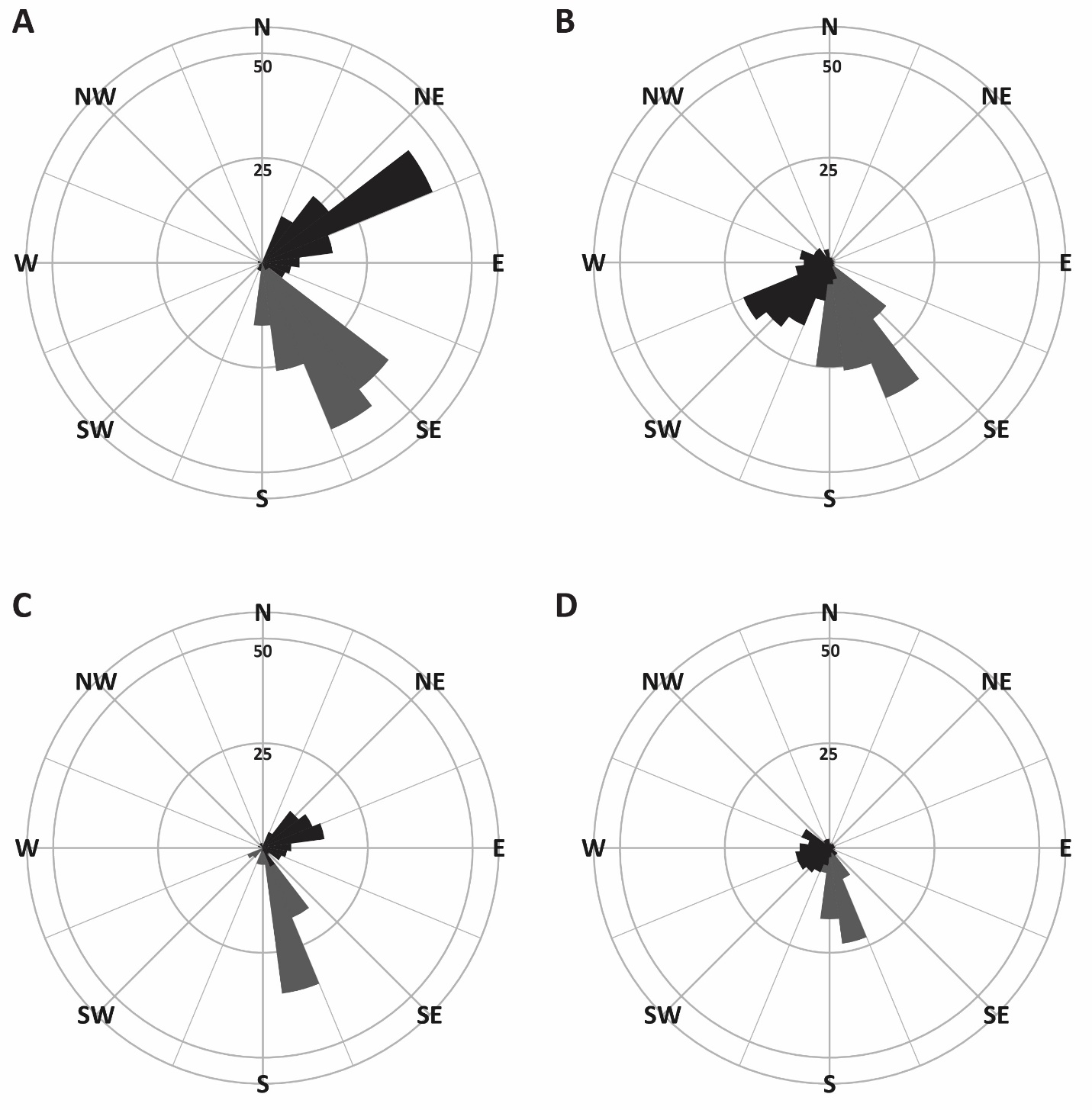


Figure S6. Flight vectors (black) and wind vectors (gray) of Nazca boobies during the A) outbound in 2015, B) inbound in 2015, C) outbound in 2016, and D) inbound in 2016. Frequencies are denoted by concentric circles (25, 50). Birds usually departed the colony toward the ENE (black in A and C) and returned toward the SSW from the ENE (black in B and D). Samples in 2016 (C and D) were smaller than in 2015.


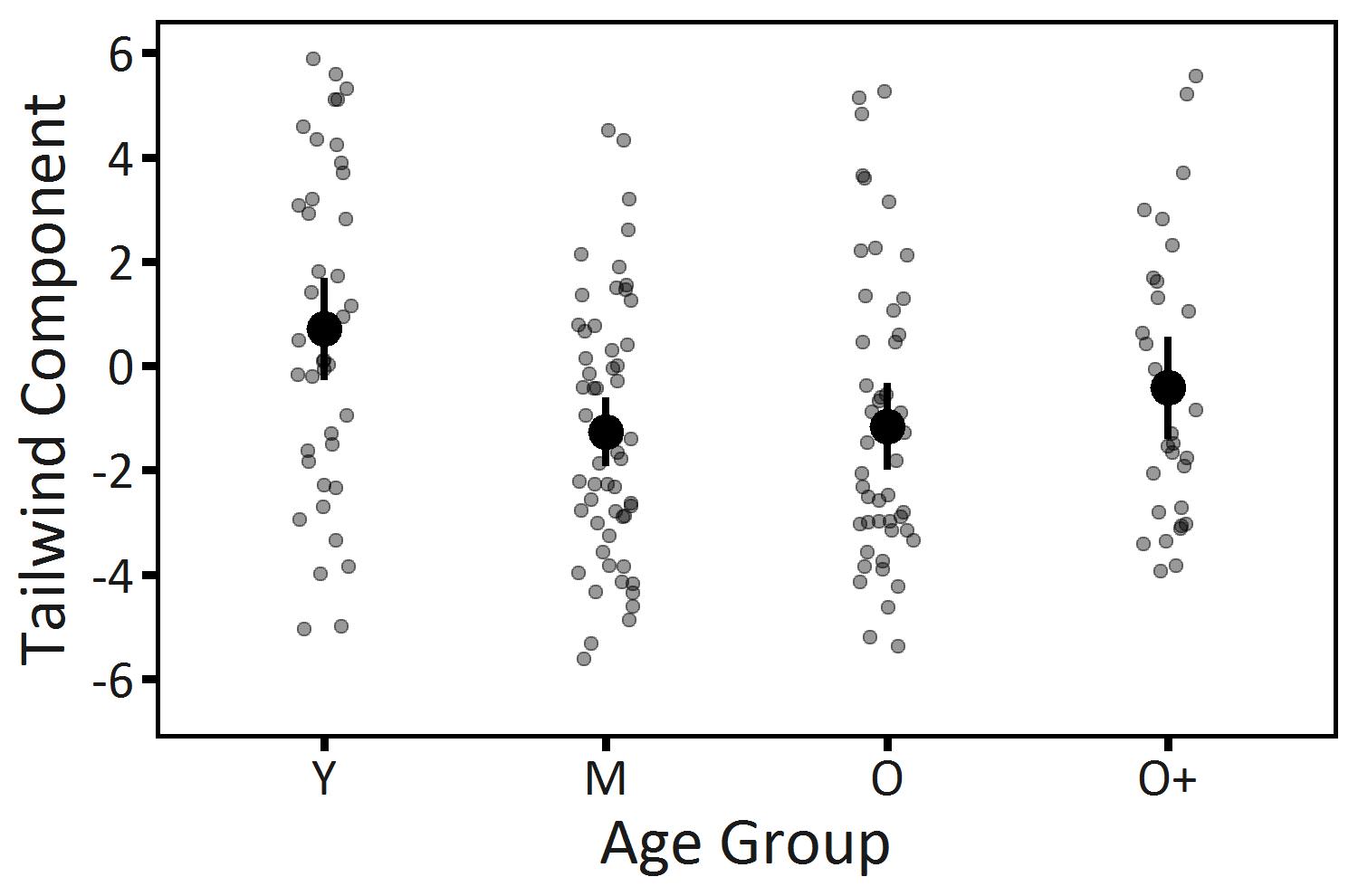


Figure S7. Tailwind Component (TWC) by AgeGroup during the inbound (Kruskal-Wallis, H = 11.66, df = 3, p < 0.01). Young (Y) birds had a more positive TWC during the inbound portion (final 30 min.) of the foraging trip, indicating a stronger tailwind during that interval compared to those of Middle Age (M), Old (O), and Oldest (O+) birds (Tukey HSD: p < 0.01). Black symbols are means predicted from a linear model ± 95% CI. Gray points are raw values (jittered horizontally) for all individuals with complete GPS tracks (n = 172).


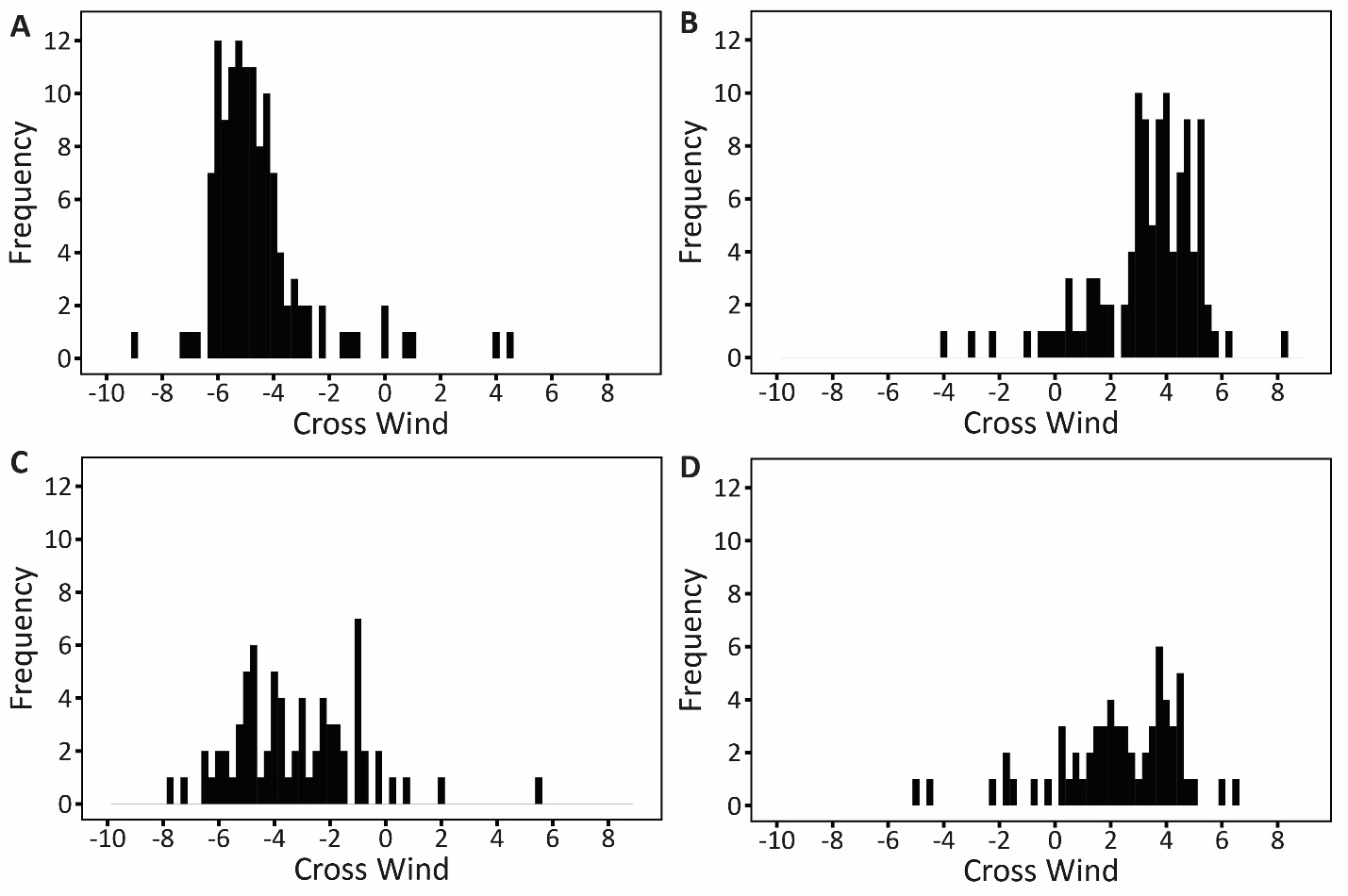


Figure S8. Frequency distribution of cross wind experienced by commuting Nazca boobies during A) outbound flight in 2015, B) inbound flight in 2015, C) outbound flight in 2016, and D) inbound flight in 2016. More negative values indicate blowing from the bird’s right and more positive values indicate blowing from the bird’s left.

## 6. Correction Made to Initial Body Mass (MassPre)

Outbound analyses exploring age effects on flight performance used body mass at the start of a foraging trip (MassPre) as an additional predictor of performance. Mass measurements from tagged individuals were collected when loggers were deployed and then corrected for the expected mass loss between deployment and departure (events which may be separated by days). The body mass correction for MassPre used the empirical daily rate of mass change during incubation from a small number of birds (n = 14, collected 2014-2017) for which two mass measures were taken during a single incubation bout (essentially fasting; Cherel et al., 1988; Prince et al., 1981). Body mass decreased rapidly immediately after returning from a foraging trip (data not shown), and more slowly after two days on the nest (Fig. S9). This pattern matches body mass loss during fasting in other bird species (Cherel et al., 1988). We used only birds that were measured when loggers were deployed (day two or three of incubation, after the period of rapid mass loss at the beginning of an incubation bout), and again several days later after continuous incubation. The daily rate of mass loss for tagged birds was similar among incubators (Fig. S9) and unrelated to the number of days between mass measurements, implying that mass declines occur at an approximately constant rate from day 2 onward (daily body mass loss = 0.006*days + 0.035, r^2^ = 0.065, p = 0.38). However, daily body mass loss increased with initial body mass (daily body mass loss = 0.068*initial body mass - 0.064, r^2^ = 0.54, p = 0.003): larger birds had a higher daily body mass loss. Therefore, rates of daily body mass loss were scaled to body size (“specific daily body mass loss”) when correcting MassPre. The mean specific daily body mass loss (0.031 kg/kg/day [95% CI: 0.027, 0.035]) was used to correct body mass at departure for all birds used in this study. Using upper and lower limits of the 95% CI resulted in a 0.02 kg difference in corrected mass, falling within the measurement error of the 2.5 kg scale, giving us confidence in this method of mass correction.

**
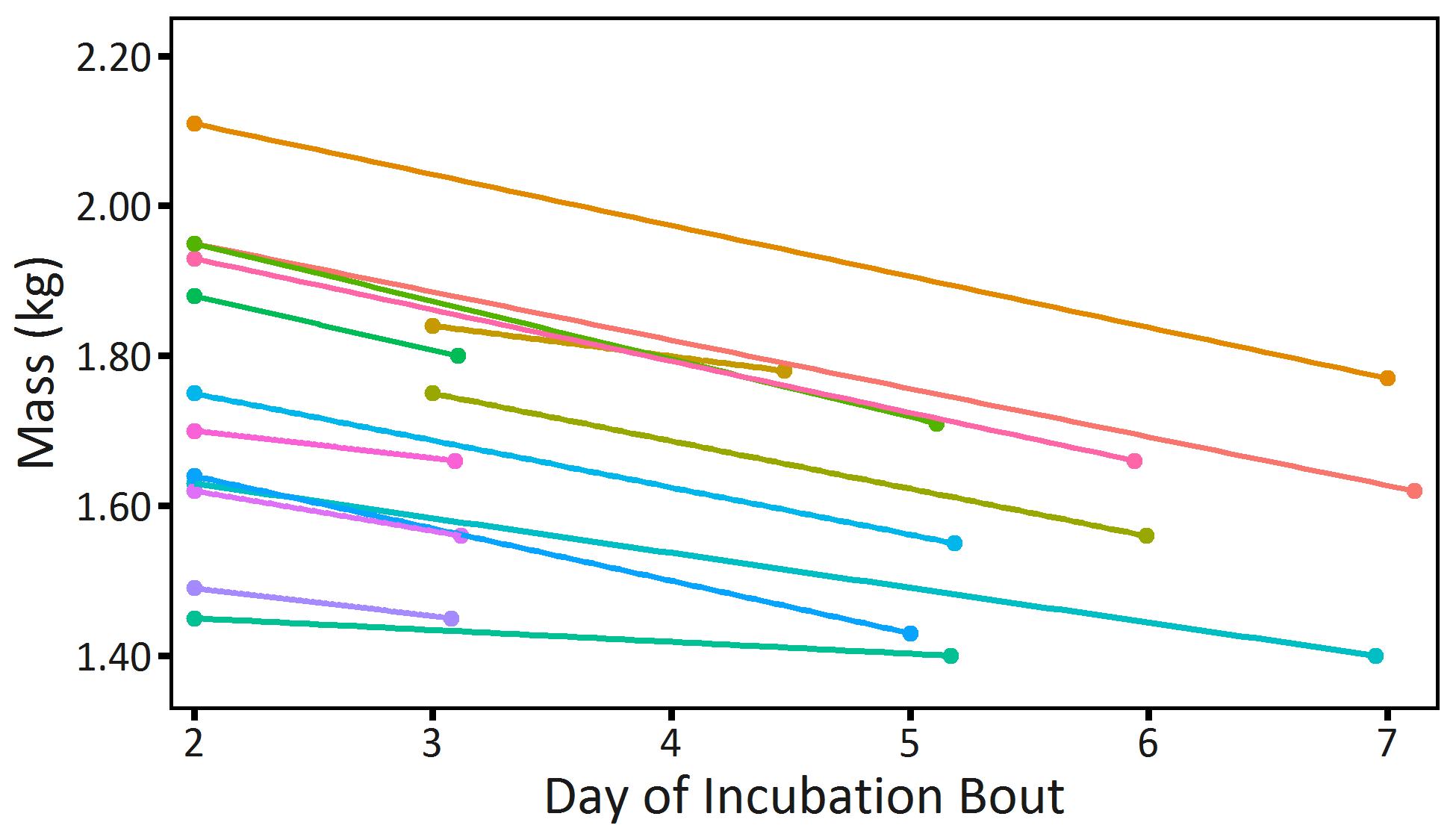
**

Figure S9. Declines in adult body mass during an incubation bout. Birds were measured once at logger deployment (day two or three of incubation) and again several days later after continuous incubation. Line colors distinguish the 14 individuals.

## 7. Estimating Wing Loading

Wing loading is a better estimate of relative wing size than either Wing Chord (a measure of wing length) or body mass separately (Van Oordt, Torres-Mura, & Hertel, 2018) and affects the constraints on achieving thrust and lift (Heerenbrink, Johansson, & Hedenstrom, 2015), explaining its relevance to understanding observed patterns of variation in flapping components (primarily by sex). Wing chord length (flattened wrist-to-tip distance) was collected from all 209 birds in this study, but measurements needed to calculate wing loading (wing span and wing area) were not. To evaluate wing loading by sex, in 2018, we measured wing morphology from 50 breeding Nazca boobies (25 males and 25 females) during the incubation period at Punta Cevallos. Body mass (kg) and wing chord (m) were measured with the bird in hand on the second or third day of incubation to minimize variation due to gut contents. A digital photograph of the outstretched right wing was taken from a position above, and perpendicular to, the wing surface using a Canon EOS 80D DSLR with a 24.2-megapixel sensor. Additional measurements were collected from the photograph following the methods outlined by Pennycuick (1989). Wing span (in m; two times the distance from the bird’s midline to the wing tip) and wing area (in m^2^; twice the area of the outstretched wing from the bird’s midline to the wing tip) were calculated in ImageJ v1.8.0, following Van Oordt et al. (2018). For these 50 birds, we calculated wing loading (N/m^2^) as mass multiplied by 9.8 m/s^2^ and divided by total wing area. Females had significantly higher wing loading than males (Fig. S10; mean_female_ =70.87; mean_male_ = 64.82; two sample t-test, t = 3.75, df = 42.57, p < 0.01). We did not measure wing area for the 209 birds tagged with accelerometers, so we used this sample of 50 birds to determine the relationship between wing area and wing chord to use Wing Loading as a predictor in our models. The Wing Loading predictor variable used in this study was derived from the relationship provided by these 50 birds and was calculated as the linear regression of wing chord on wing area: (corrected body mass at departure * 9.8) / (-0.20 + 0.92*wing chord). Wing chord explained 49% of the variation in wing area (r^2^ = 0.49).

**
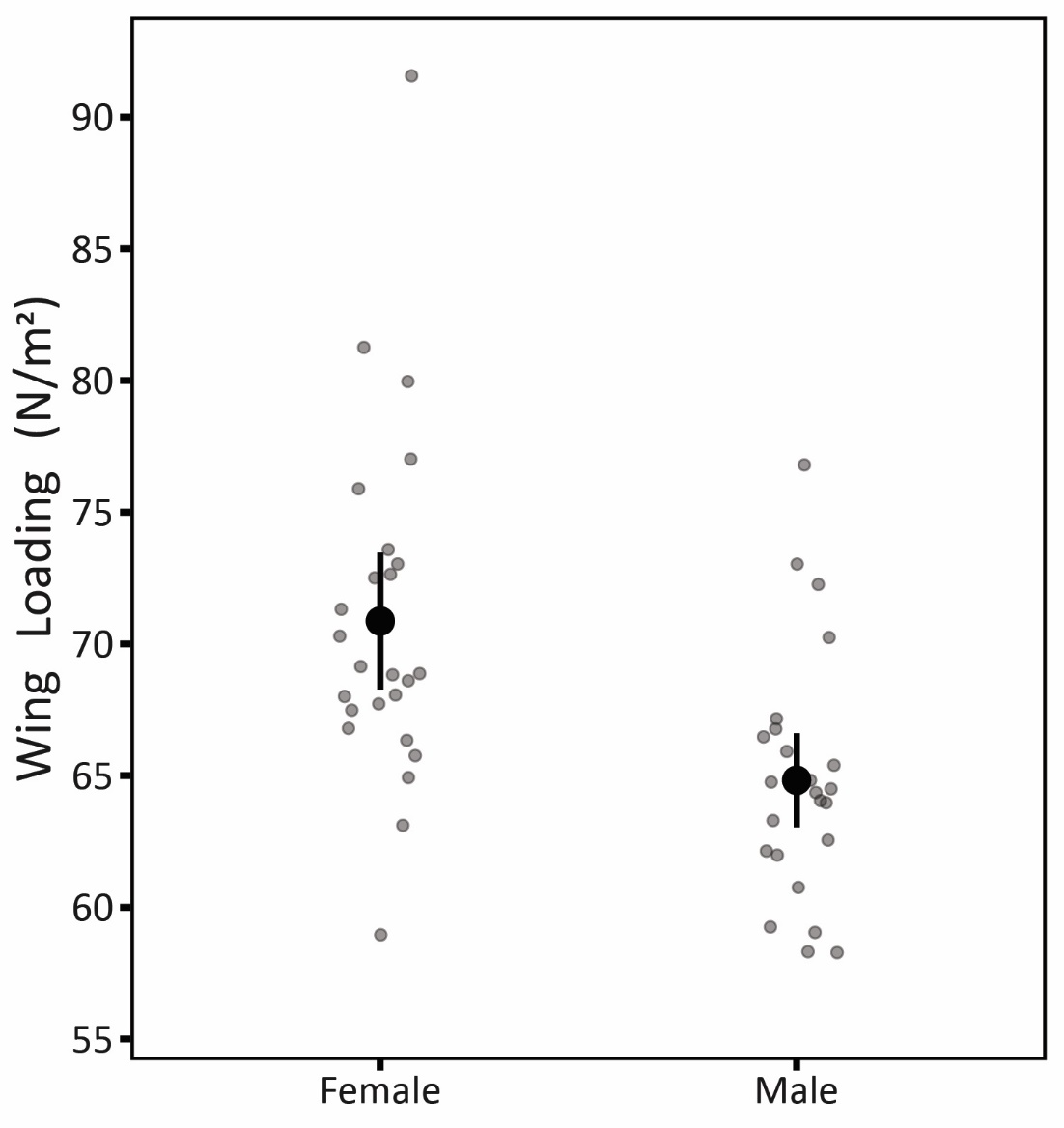
**

Figure S10. Sex differences in wing loading (N/m^2^). Small points are the raw data and larger points are the mean values ± 95% CI. Raw data (n = 50) are jittered horizontally for visual clarity.

## 8. Patterns of Covariation among Predictor Variables

Behavioral and morphological changes with age have been observed in seabirds (e.g., choice of foraging location; Lecomte et al., 2010), suggesting that age could covary with other predictors included in models of foraging outcomes and flight components. If not accounted for, covariation between age and other fixed-effect predictors (TWC, Wing Loading, etc.) could make it difficult to disentangle age effects from those of Wing Loading (for example, when predictors are included jointly in models). Pairwise comparisons between predictor variables are reported in Table S4. When both variables were continuous, Pearson’s correlation tests were used to measure their association with each other. When one variable was the factor AgeGroup, we used ANOVA or Kruskal-Wallis models to assess the degree of association. Finally, when one variable was the factor Sex, we used a Wilcoxon rank sum test to test for differences in the distribution of each other predictor variable by Sex.

Table S4. Pairwise comparisons of predictors included in linear regression models, separated by flapping period. Statistically significant results are in bold. Data from males (“m”) and females (“f”) were analyzed separately when appropriate.

| **Predictor 1** | **Predictor 2** | **Outbound** | **Inbound** |
| --- | --- | --- | --- |
| AgeGroup | TWC | H = 3.50, df = 3, p = 0.32 | **H = 13.71, df = 3, p = 0.003** |
| AgeGroup | Wing Loading (f) | F_3, 102_ = 1.10, p = 0.35 | **F_3, 95_ = 3.29, p = 0.02** |
| AgeGroup | Wing Loading (m) | H = 1.51, df = 3, p = 0.68 | F_3, 91_ = 1.19, p = 0.32 |
| AgeGroup | Date | H = 50.61, df = 57, p = 0.71 | H = 56.04, df = 55, p = 0.44 |
| Sex | TWC | W = 4833.5, N_1_ = 103, N_2_ = 106, p = 0.15 | W = 5260, N_1_ = 95, N_2_ = 99, p = 0.15 |
| Sex | Date | W = 4965.5, N_1_ = 103, N_2_ = 106, p = 0.26 | W = 4491, N_1_ = 95, N_2_ = 99, p = 0.59 |

### 8.1 Age and Environment

Young adults had a more positive TWC (stronger tailwind) than did Middle Age and Old birds during the inbound period (Fig. S7; Dunn’s test, Young-Middle Age: mean rank diff. =

-3.04, p = 0.01; Young-Old: mean rank diff. = -2.94, p = 0.01). TWC was still incorporated into inbound models as a conservative test of age influences on flight performance.

### 8.2 Age and Wing Loading

On the outbound, Wing Loading was not related to AgeGroup in females or males. On the inbound, Wing Loading was related to AgeGroup in females (Table S4). To remove the age effects on Wing Loading before analysis, raw Wing Loading was standardized separately by Sex and AgeGroup before inclusion in inbound AgeGroup Models.

### 8.3 Body Size and Sex

Females are 16% larger than males (body mass; Anderson 1993) and, as expected, mass at departure, wing chord, and Wing Loading were significantly larger in females (Fig. S11). Females in this study were 17.0% larger than males in mass at departure, 3.5% larger in wing chord, and 9.7% larger in Wing Loading. Therefore, Wing Loading was standardized within Sex (or by AgeGroup and Sex, see above).


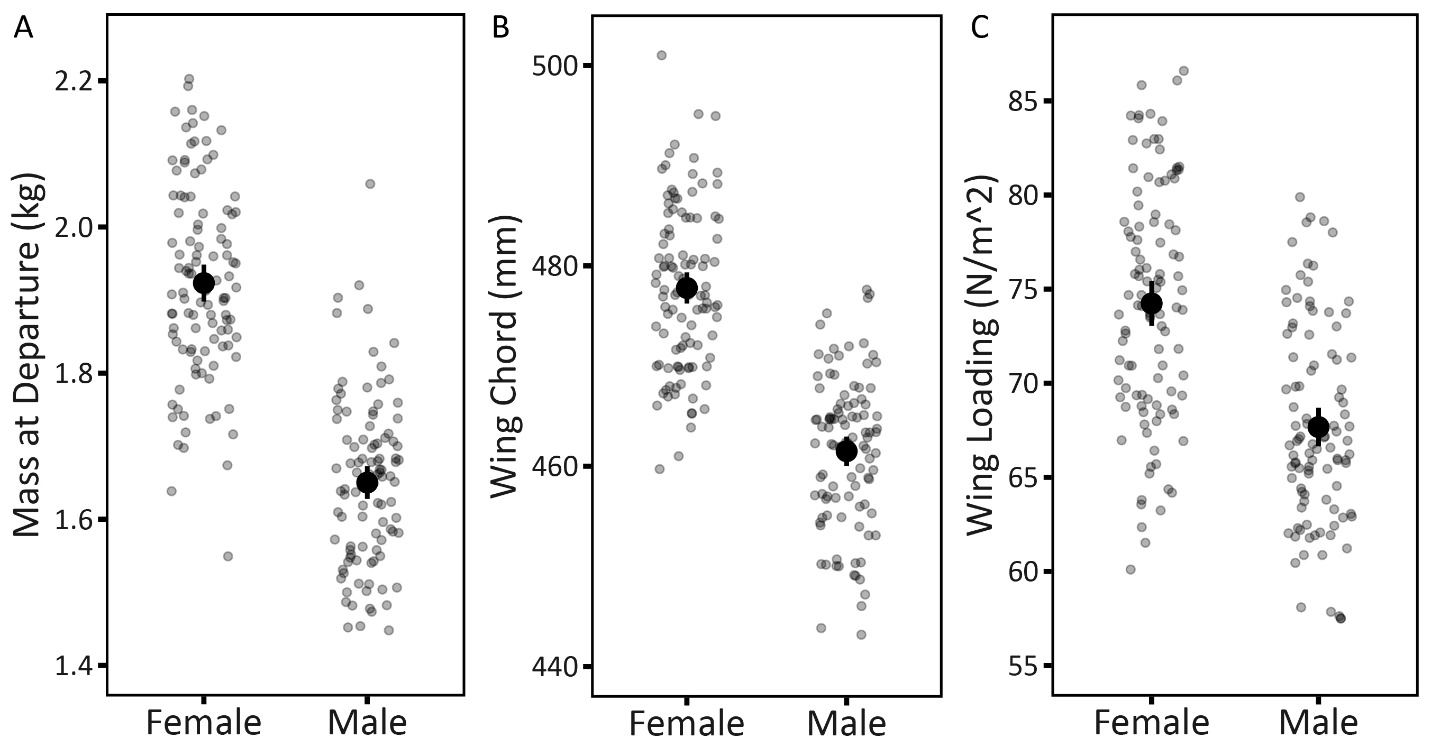


Figure S11. Sex differences in A) Mass at Departure, B) Wing Chord, and C) Wing Loading in Nazca boobies. Small points are the raw data (jittered horizontally) and larger points are the mean values with 95% CI.

# SUPPLEMENTARY RESULTS

We focus on candidate models falling within 4 AICc units of the top model (see main text) because the categorical AgeGroup variable adds three parameters to the model. Each parameter (K) is penalized 2 AICc, as AIC = -2log(L) + 2K (Burnham & Anderson, 2002); therefore the three-level AgeGroup variable gets penalized six AICc units for the additional complexity it adds to the model. If only one of the age classes (Young, Old, Oldest) shows distinct performance differences from the Middle Age group (the baseline factor level), then a model with AgeGroup vs. one without AgeGroup could fall 4 AICc units away from the top model, despite showing limited (but relevant) age effects (e.g., early-life improvement only, no late-life decline). Furthermore, Burnham, Anderson, & Huyvaert (2011) suggested that models within 4 AICc units of the top model represent plausible hypotheses.

## 9. Effects of Incubation Days on Response Variables

In a *post hoc* analysis, we evaluated incubation length as a predictor of flight components and foraging outcomes, responding to a reviewer’s comment. All birds in this study were incubating breeders, so incubation length was represented by the continuous variable of number of days since clutch initiation (“Incubation Days”). Incubation Days was correlated with Date (r = 0.29, df = 207, p < 0.01) so was not included in the original models. Like the other continuous variables in our analyses, Incubation Days was standardized (zero mean, unit standard deviation) before analysis. We evaluated the performance of the global (most complex) model, and the global model including Incubation Days, using AICc and examined the coefficient estimates (β) and 95% CI of each focal fixed effect (AgeGroup, Sex, Breeding Season, and Wing Loading). If inclusion of Incubation Days in the full model caused the standard errors of β to move away from zero, we would consider Incubation Days to improve model performance. In this scenario, the full model set should be analyzed again with additional models testing for Incubation Days’ power as an explanatory predictor. We found no meaningful change in the coefficients of any foraging and flight parameter when models included Incubation Days (Tables S5-S9). Therefore, models with Incubation Days were not considered further in analysis.

Table S5**.** Coefficient estimates and 95% CI for fixed effects comparing two models (global model versus global model + Incubation Days) explaining variation in foraging outcomes. Global model: response variable ~ Date + AgeGroup + Sex + Wing Loading + Breeding Season + AgeGroup*Sex + AgeGroup*Breeding Season.

|  | **Mass Gain/hr** | | **Mass Gain** | | **Absence Duration** | |
| --- | --- | --- | --- | --- | --- | --- |
| **Fixed Effect** | **Global Model** | **Global Model**  **+ Incubation Days** | **Global Model** | **Global Model**  **+ Incubation Days** | **Global Model** | **Global Model**  **+ Incubation Days** |
| **Intercept** | 1.64 [1.55, 1.73] | 1.64 [1.55, 1.73] | 406.28 [336.05, 476.51] | 405.93 [336.22, 475.64] | 1.39 [1.23, 1.55] | 1.40 [1.24, 1.55] |
| **AgeG**  **(Young)** | -0.12 [-0.24, 0.00] | -0.12 [-0.24, 0.00] | -56.24 [-149.85, 37.37] | -58.52 [-151.46, 34.42] | 0.02 [-0.21, 0.24] | 0.00 [-0.22, 0.22] |
| **AgeG**  **(Old)** | -0.07 [-0.19, 0.06] | -0.07 [-0.20, 0.06] | -68.58 [-166.77, 29.61] | -63.09 [-160.72, 34.54] | 0.00 [-0.23, 0.22] | 0.01 [-0.23, 0.22] |
| **AgeG**  **(Oldest)** | -0.07 [-0.21, 0.06] | -0.08 [-0.21, 0.06] | -78.83 [-184.54, 26.87] | -73.95 [-178.99, 31.10] | -0.01 [-0.26, 0.23] | 0.00 [-0.25, 0.24] |
| **Sex**  **(Male)** | -0.03 [-0.13, 0.07] | -0.03 [-0.13, 0.08] | -86.57 [-167.71, -5.43] | -86.59 [-167.12, -6.05] | 0.01 [-0.18, 0.20] | 0.00 [-0.18, 0.19] |
| **Wing Loading** | 0.00 [0.00, 0.01] | 0.00 [0.00, 0.01] | -13.04 [-17.34, -8.73] | -12.51 [-16.82, -8.20] | -0.03 [-0.04, -0.02] | -0.03 [-0.04, -0.02] |
| **Season**  **(2016)** | -0.10 [-0.20, 0.01] | -0.10 [-0.20, 0.01] | 2.19 [-80.04, 84.43] | 9.41 [-72.57, 91.39] | 0.24 [0.05, 0.44] | 0.26 [0.07, 0.46] |
| **Date** | 0.04 [0.01, 0.07] | 0.04 [0.01, 0.07] | 34.42 [11.56, 57.27] | 40.34 [16.80, 63.88] | -0.05 [-0.11, 0.00] | -0.04 [-0.09, 0.02] |
| **AgeG*Sex**  **(Young, Male)** | 0.08 [-0.07, 0.23] | 0.09 [-0.06, 0.24] | 22.23 [-94.12, 138.58] | 11.28 [-104.78, 127.35] | 0.00 [-0.28, 0.28] | -0.01 [-0.29, 0.27] |
| **AgeG*Sex**  **(Old, Male)** | 0.05 [-0.09, 0.20] | 0.06 [-0.09, 0.20] | 29.61 [-84.29, 143.51] | 21.62 [-91.75, 134.98] | -0.09 [-0.36, 0.17] | -0.11 [-0.37, 0.15] |
| **AgeG*Sex**  **(Oldest, Male)** | 0.05 [-0.11, 0.21] | 0.06 [-0.10, 0.22] | 74.00 [-50.82, 198.81] | 64.31 [-60.01, 188.62] | -0.02 [-0.32, 0.28] | -0.04 [-0.34, 0.25] |
| **AgeG*Season**  **(Young, 2016)** | 0.08 [-0.08, 0.25] | 0.09 [-0.08, 0.25] | 106.62 [-20.21, 233.44] | 102.11 [-23.86, 228.07] | 0.14 [-0.17, 0.45] | 0.14 [-0.17, 0.44] |
| **AgeG*Season**  **(Old, 2016)** | -0.01 [-0.15, 0.14] | 0.00 [-0.15, 0.14] | 32.99 [-81.18, 147.15] | 25.62 [-87.96, 139.20] | 0.32 [0.05, 0.59] | 0.31 [0.042, 0.58] |
| **AgeG*Season**  **(Oldest, 2016)** | 0.03 [-0.13, 0.19] | 0.03 [-0.13, 0.19] | -15.62 [-140.31, 109.08] | -15.84 [-139.60, 107.93] | 0.12 [-0.19, 0.42] | 0.11 [-0.19, 0.41] |
| **Incubation Days** | - | 0.01 [-0.02, 0.04] | - | -21.62 [-44.66, 1.42] | - | -0.06 [-0.11, 0.00] |

Table S6**.** Coefficient estimates and 95% CI for fixed effects comparing two models (global model versus global model + Incubation Days) explaining variation in outbound Airspeed and Wingbeat Frequency. Global model: response variable ~ TWC + Date + AgeGroup + Sex + Wing Loading + Breeding Season + AgeGroup*Sex + AgeGroup*Breeding Season.

|  | **Airspeed** | | **Wingbeat Frequency** | | |  |
| --- | --- | --- | --- | --- | --- | --- |
| **Fixed Effect** | **Global Model** | **Global Model**  **+ Incubation Days** | | **Global Model** | **Global Model**  **+ Incubation Days** | |
| **Intercept** | 12.79 [12.39, 13.20] | 12.79 [12.38, 13.20] | | 3.76 [3.71, 3.81] | 3.76 [3.71, 3.81] | |
| **AgeG**  **(Young)** | -0.50 [-0.56, -0.45] | -0.50 [-0.55, -0.45] | | 0.00 [0.00, 0.01] | 0.00 [0.00, 0.01] | |
| **AgeG**  **(Old)** | -0.25 [-0.82, 0.33] | -0.22 [-0.79, 0.35] | | -0.02 [-0.09, 0.05] | -0.02 [-0.09, 0.05] | |
| **AgeG**  **(Oldest)** | 0.15 [-0.41, 0.70] | 0.14 [-0.41, 0.69] | | -0.05 [-0.12, 0.02] | -0.05 [-0.12, 0.02] | |
| **Sex**  **(Male)** | 0.23 [-0.42, 0.87] | 0.21 [-0.44, 0.85] | | -0.04 [-0.11, 0.04] | -0.04 [-0.11, 0.04] | |
| **Wing Loading** | 0.13 [-0.34, 0.60] | 0.13 [-0.34, 0.60] | | 0.10 [0.04, 0.15] | 0.09 [0.04, 0.15] | |
| **Season**  **(2016)** | 0.02 [-0.01, 0.04] | 0.01 [-0.01, 0.04] | | 0.00 [0.00, 0.00] | 0.00 [0.00, 0.00] | |
| **Date** | -0.43 [-0.91, 0.06] | -0.47 [-0.96, 0.01] | | -0.06 [-0.12, -0.01] | -0.06 [-0.12, 0.00] | |
| **AgeG*Sex**  **(Young, Male)** | 0.02 [-0.12, 0.16] | -0.01 [-0.15, 0.14] | | 0.00 [-0.01, 0.02] | 0.00 [-0.01, 0.02] | |
| **AgeG*Sex**  **(Old, Male)** | -0.39 [-1.11, 0.32] | -0.36 [-1.07, 0.35] | | -0.01 [-0.10, 0.07] | -0.01 [-0.10, 0.07] | |
| **AgeG*Sex**  **(Oldest, Male)** | -0.10 [-0.76, 0.55] | -0.06 [-0.72, 0.60] | | 0.07 [-0.01, 0.15] | 0.07 [-0.01, 0.15] | |
| **AgeG*Season**  **(Young, 2016)** | -0.11 [-0.87, 0.66] | -0.06 [-0.83, 0.70] | | 0.06 [-0.03, 0.15] | 0.06 [-0.03, 0.15] | |
| **AgeG*Season**  **(Old, 2016)** | 1.03 [0.23, 1.83] | 1.06 [0.26, 1.86] | | -0.01 [-0.10, 0.09] | -0.01 [-0.10, 0.09] | |
| **AgeG*Season**  **(Oldest, 2016)** | -0.36 [-1.04, 0.31] | -0.34 [-1.01, 0.33] | | -0.02 [-0.11, 0.06] | -0.03 [-0.11, 0.06] | |
| **Incubation Days** | - | -0.26 [-1.04, 0.53] | | - | 0.01 [-0.09, 0.10] | |

Table S7**.** Coefficient estimates and 95% CI for fixed effects comparing two models (global model versus global model + Incubation Days) explaining variation in outbound Flap-Glide Ratio, Body Displacement, and Flapping ODBA. Global model: response variable ~ TWC + Date + AgeGroup + Sex + Wing Loading + Breeding Season + AgeGroup*Sex + AgeGroup*Breeding Season.

|  | **Flap-Glide Ratio** | | **Body Displacement** | | **Flapping ODBA** | |
| --- | --- | --- | --- | --- | --- | --- |
| **Fixed Effect** | **Global Model** | **Global Model**  **+ Incubation Days** | **Global Model** | **Global Model**  **+ Incubation Days** | **Global Model** | **Global Model**  **+ Incubation Days** |
| **Intercept** | 0.13 [0.06, 0.19] | 0.12 [0.06, 0.19] | 1.53 [1.48, 1.59] | 1.53 [1.48, 1.59] | 18.16 [17.71, 18.60] | 18.17 [17.73, 18.614] |
| **AgeG**  **(Young)** | -0.01 [-0.02, 0.00] | -0.01 [-0.02, 0.00] | 0.02 [0.01, 0.02] | 0.02 [0.01, 0.02] | 0.13 [0.07, 0.19] | 0.13 [0.07, 0.18] |
| **AgeG**  **(Old)** | 0.06 [-0.03, 0.14] | 0.06 [-0.03, 0.14] | -0.01 [-0.09, 0.07] | -0.01 [-0.09, 0.07] | 0.20 [-0.44, 0.83] | 0.16 [-0.47, 0.80] |
| **AgeG**  **(Oldest)** | 0.01 [-0.08, 0.09] | 0.01 [-0.08, 0.09] | -0.02 [-0.09, 0.06] | -0.02 [-0.09, 0.06] | -0.01 [-0.63, 0.61] | -0.01 [-0.62, 0.61] |
| **Sex**  **(Male)** | 0.06 [-0.04, 0.15] | 0.05 [-0.04, 0.15] | 0.08 [-0.01, 0.16] | 0.07 [-0.02, 0.16] | 0.06 [-0.65, 0.77] | 0.09 [-0.62, 0.79] |
| **Wing Loading** | 0.00 [-0.07, 0.07] | 0.00 [-0.07, 0.07] | 0.02 [-0.04, 0.09] | 0.03 [-0.04, 0.09] | 0.39 [-0.14, 0.91] | 0.37 [-0.15, 0.89] |
| **Season**  **(2016)** | 0.01 [0.00, 0.01] | 0.01 [0.00, 0.01] | 0.00 [0.00, 0.01] | 0.00 [0.00, 0.01] | 0.00 [-0.02, 0.03] | 0.01 [-0.02, 0.04] |
| **Date** | 0.16 [0.09, 0.24] | 0.16 [0.08, 0.23] | -0.05 [-0.12, 0.02] | -0.06 [-0.13, 0.01] | -0.40 [-0.94, 0.14] | -0.34 [-0.89, 0.20] |
| **AgeG*Sex**  **(Young, Male)** | 0.00 [-0.02, 0.02] | 0.00 [-0.02, 0.02] | 0.00 [-0.02, 0.02] | -0.01 [-0.03, 0.02] | 0.10 [-0.06, 0.25] | 0.14 [-0.02, 0.29] |
| **AgeG*Sex**  **(Old, Male)** | -0.08 [-0.19, 0.03] | -0.08 [-0.19, 0.03] | 0.03 [-0.07, 0.13] | 0.03 [-0.07, 0.14] | 0.21 [-0.59, 1.01] | 0.17 [-0.63, 0.96] |
| **AgeG*Sex**  **(Oldest, Male)** | -0.03 [-0.13, 0.07] | -0.03 [-0.13, 0.08] | 0.01 [-0.09, 0.10] | 0.01 [-0.08, 0.10] | 0.31 [-0.43, 1.05] | 0.26 [-0.48, 1.00] |
| **AgeG*Season**  **(Young, 2016)** | -0.07 [-0.19, 0.05] | -0.07 [-0.19, 0.05] | 0.02 [-0.08, 0.13] | 0.03 [-0.08, 0.13] | 0.02 [-0.83, 0.871] | -0.03 [-0.88, 0.82] |
| **AgeG*Season**  **(Old, 2016)** | -0.08 [-0.21, 0.04] | -0.08 [-0.20, 0.04] | 0.03 [-0.09, 0.14] | 0.03 [-0.08, 0.14] | 0.29 [-0.60, 1.17] | 0.25 [-0.63, 1.14] |
| **AgeG*Season**  **(Oldest, 2016)** | -0.02 [-0.13, 0.08] | -0.02 [-0.13, 0.08] | 0.02 [-0.08, 0.11] | 0.02 [-0.08, 0.12] | 0.09 [-0.67, 0.85] | 0.06 [-0.69, 0.82] |
| **Incubation Days** | - | -0.06 [-0.18, 0.07] | - | -0.08 [-0.19, 0.03] | - | -0.14 [-1.00, 0.73] |

Table S8**.** Coefficient estimates and 95% CI for fixed effects comparing two models (global model versus global model + Incubation Days) explaining variation in inbound Airspeed and Wingbeat Frequency. Global model: response variable ~ TWC + Date + AgeGroup + Sex + Wing Loading + Breeding Season + AgeGroup*Sex + AgeGroup*Breeding Season.

|  | **Airspeed** | | **Wingbeat Frequency** | |
| --- | --- | --- | --- | --- |
| **Fixed Effect** | **Global Model** | **Global Model**  **+ Incubation Days** | **Global Model** | **Global Model**  **+ Incubation Days** |
| **Intercept** | 13.88 [13.38, 14.38] | 13.87 [13.38, 14.37] | 3.78 [3.72, 3.84] | 3.78 [3.72, 3.843] |
| **AgeG**  **(Young)** | -0.34 [-0.40, -0.28] | -0.34 [-0.40, -0.28] | -0.01 [-0.01, 0.00] | -0.01 [-0.02, 0.00] |
| **AgeG**  **(Old)** | -0.28 [-0.98, 0.43] | -0.26 [-0.97, 0.44] | -0.02 [-0.11, 0.07] | -0.02 [-0.11, 0.07] |
| **AgeG**  **(Oldest)** | -0.19 [-0.89, 0.51] | -0.20 [-0.90, 0.50] | -0.05 [-0.14, 0.03] | -0.05 [-0.14, 0.03] |
| **Sex**  **(Male)** | -0.32 [-1.14, 0.50] | -0.31 [-1.13, 0.51] | -0.04 [-0.14, 0.06] | -0.04 [-0.14, 0.06] |
| **Wing Loading** | -0.10 [-0.66, 0.47] | -0.09 [-0.66, 0.47] | 0.05 [-0.02, 0.12] | 0.05 [-0.02, 0.12] |
| **Season**  **(2016)** | 0.04 [0.02, 0.07] | 0.04 [0.02, 0.07] | 0.00 [0.00, 0.01] | 0.00 [0.00, 0.01] |
| **Date** | 0.66 [0.08, 1.23] | 0.64 [0.06, 1.21] | -0.06 [-0.13, 0.02] | -0.06 [-0.14, 0.01] |
| **AgeG*Sex**  **(Young, Male)** | -0.01 [-0.02, 0.00] | -0.01 [-0.02, 0.00] | 0.03 [0.00, 0.05] | 0.02 [0.00, 0.05] |
| **AgeG*Sex**  **(Old, Male)** | -0.12 [-0.99, 0.75] | -0.10 [-0.97, 0.78] | 0.08 [-0.03, 0.20] | 0.08 [-0.03, 0.20] |
| **AgeG*Sex**  **(Oldest, Male)** | -0.15 [-0.99, 0.68] | -0.11 [-0.95, 0.73] | 0.09 [-0.02, 0.20] | 0.09 [-0.02, 0.20] |
| **AgeG*Season**  **(Young, 2016)** | -0.55 [-1.49, 0.41] | -0.52 [-1.47, 0.43] | 0.09 [-0.03, 0.22] | 0.09 [-0.03, 0.22] |
| **AgeG*Season**  **(Old, 2016)** | 0.59 [-0.48, 1.65] | 0.61 [-0.46, 1.68] | 0.01 [-0.13, 0.14] | 0.01 [-0.13, 0.14] |
| **AgeG*Season**  **(Oldest, 2016)** | -0.74 [-1.59, 0.12] | -0.73 [-1.59, 0.12] | -0.03 [-0.14, 0.08] | -0.03 [-0.14, 0.08] |
| **Incubation Days** | - | -0.66 [-1.62, 0.30] | - | -0.02 [-0.15, 0.10] |

Table S9**.** Coefficient estimates and 95% CI for fixed effects comparing two models (global model versus global model + Incubation Days) explaining variation in inbound Flap-Glide Ratio, Body Displacement, and Flapping ODBA. Global model: response variable ~ TWC + Date + AgeGroup + Sex + Wing Loading + Breeding Season + AgeGroup*Sex + AgeGroup*Breeding Season.

|  | **Flap-Glide Ratio** | | **Body Displacement** | | **Flapping ODBA** | |
| --- | --- | --- | --- | --- | --- | --- |
| **Fixed Effect** | **Global Model** | **Global Model**  **+ Incubation** | **Global Model** | **Global Model**  **+ Incubation** | **Global Model** | **Global Model**  **+ Incubation** |
| **Intercept** | 0.20 [0.14, 0.26] | 0.20 [0.14, 0.26] | 1.43 [1.37, 1.49] | 1.42 [1.37, 1.48] | 1.43 [1.37, 1.49] | 17.49 [17.05, 17.92] |
| **AgeG**  **(Young)** | -0.01 [-0.02, -0.01] | -0.01 [-0.02, -0.01] | 0.01 [0.00, 0.02] | 0.01 [0.00, 0.02] | 0.01 [0.00, 0.02] | 0.08 [0.02, 0.13] |
| **AgeG**  **(Old)** | 0.03 [-0.06, 0.12] | 0.03 [-0.07, 0.11] | -0.01 [-0.10, 0.08] | -0.01 [-0.09, 0.08] | -0.01 [-0.10, 0.08] | 0.25 [-0.40, 0.89] |
| **AgeG**  **(Oldest)** | -0.01 [-0.10, 0.07] | -0.02 [-0.10, 0.07] | 0.02 [-0.06, 0.11] | 0.024 [-0.06, 0.11] | 0.02 [-0.06, 0.11] | 0.38 [-0.24, 0.99] |
| **Sex**  **(Male)** | 0.02 [-0.08, 0.12] | 0.02 [-0.08, 0.12] | 0.03 [-0.07, 0.13] | 0.03 [-0.07, 0.13] | 0.03 [-0.07, 0.13] | -0.08 [-0.80, 0.64] |
| **Wing Loading** | 0.05 [-0.02, 0.12] | 0.05 [-0.02, 0.12] | -0.01 [-0.08, 0.07] | 0.00 [-0.07, 0.07] | -0.01 [-0.08, 0.07] | 0.14 [-0.37, 0.65] |
| **Season**  **(2016)** | 0.00 [0.00, 0.01] | 0.00 [0.00, 0.01] | 0.00 [0.00, 0.00] | 0.00 [-0.01, 0.00] | 0.00 [-0.01, 0.00] | -0.01 [-0.03, 0.01] |
| **Date** | 0.08 [0.01, 0.16] | 0.08 [0.011, 0.16] | -0.04 [-0.11, 0.04] | -0.04 [-0.12, 0.03] | -0.04 [-0.11, 0.04] | -0.20 [-0.73, 0.32] |
| **AgeG*Sex**  **(Young, Male)** | -0.02 [-0.04, 0.00] | -0.02 [-0.04, 0.01] | -0.01 [-0.03, 0.01] | -0.02 [-0.04, 0.00] | -0.01 [-0.03, 0.01] | 0.035 [-0.13, 0.20] |
| **AgeG*Sex**  **(Old, Male)** | -0.06 [-0.17, 0.05] | -0.07 [-0.18, 0.05] | 0.00 [-0.11, 0.11] | 0.01 [-0.10, 0.12] | 0.00 [-0.11, 0.11] | -0.27 [-1.07, 0.54] |
| **AgeG*Sex**  **(Oldest, Male)** | 0.02 [-0.09, 0.13] | 0.02 [-0.09, 0.12] | -0.02 [-0.13, 0.08] | -0.01 [-0.12, 0.09] | -0.02 [-0.13, 0.08] | -0.17 [-0.93, 0.58] |
| **AgeG*Season**  **(Young, 2016)** | -0.13 [-0.25, -0.01] | -0.13 [-0.26, -0.01] | 0.05 [-0.07, 0.17] | 0.06 [-0.06, 0.18] | 0.05 [-0.07, 0.17] | 0.44 [-0.43, 1.32] |
| **AgeG*Season**  **(Old, 2016)** | -0.03 [-0.16, 0.10] | -0.03 [-0.16, 0.10] | -0.04 [-0.17, 0.09] | -0.04 [-0.17, 0.09] | -0.04 [-0.17, 0.09] | -0.04 [-0.99, 0.92] |
| **AgeG*Season**  **(Oldest, 2016)** | -0.07 [-0.18, 0.04] | -0.07 [-0.18, 0.04] | 0.02 [-0.09, 0.13] | 0.02 [-0.09, 0.12] | 0.02 [-0.09, 0.13] | 0.16 [-0.62, 0.94] |
| **Incubation Days** | - | 0.11 [-0.01, 0.24] | - | -0.02 [-0.14, 0.10] | - | 0.30 [-0.58, 1.18] |

## **10. Complete Model Results:** Mass Gain Rate

Table S10. Candidate models explaining variation in mass gained per hour while on a foraging absence (log(MassGain/hr)), ranked by AICc. Models within ΔAICc of 4 of the top model are considered to be highly supported (in bold). The number of parameters (*k*), AICc difference from the top model (ΔAICc), and Akaike weights (*ω_i_*) are reported. In contrast with the main text, main effects with interactions are written out in full (e.g., AgeGroup + Season + AgeGroup*Season is written as “AgeGroup + Season + AgeGroup*Season”). Season = Breeding Season.

| *Log(MassGain/hr): all ages* |  |  |  |
| --- | --- | --- | --- |
| **Model** | ***k*** | **ΔAICc** | **ωi** |
| **Date + Season** | **4** | **0.00** | **0.35** |
| **Date + Wing Loading + Season** | **5** | **1.17** | **0.19** |
| **Date + Sex + Season** | **5** | **1.68** | **0.15** |
| **Date + Sex + Wing Loading + Season** | **6** | **2.84** | **0.08** |
| **Date + AgeGroup + Season** | **7** | **3.35** | **0.06** |
| Date + Wing Loading | 4 | 4.63 | 0.03 |
| Date + AgeGroup + Wing Loading + Season | 8 | 4.86 | 0.03 |
| Date + AgeGroup + Sex + Season | 8 | 5.20 | 0.03 |
| Date | 3 | 5.82 | 0.02 |
| Date + Sex + Wing Loading | 5 | 6.25 | 0.02 |
| Date + AgeGroup + Sex + Wing Loading + Season | 9 | 6.69 | 0.01 |
| Date + Sex | 4 | 7.48 | 0.01 |
| Date + AgeGroup + Season + AgeGroup*Season | 10 | 8.61 | 0.00 |
| Date + AgeGroup + Wing Loading | 7 | 9.23 | 0.00 |
| Date + AgeGroup + Wing Loading + Season + AgeGroup*Season | 11 | 10.12 | 0.00 |
| Date + AgeGroup | 6 | 10.14 | 0.00 |
| Date + AgeGroup + Sex + Season + AgeGroup*Season | 11 | 10.54 | 0.00 |
| Date + AgeGroup + Sex + AgeGroup*Sex + Season | 11 | 10.71 | 0.00 |
| Date + AgeGroup + Sex + Wing Loading | 8 | 10.98 | 0.00 |
| Date + AgeGroup + Sex | 7 | 11.96 | 0.00 |
| Date + AgeGroup + Sex + Wing Loading + Season + AgeGroup*Season | 12 | 12.03 | 0.00 |
| Date + AgeGroup + Sex + Wing Loading + AgeGroup*Sex + Season | 12 | 12.31 | 0.00 |
| Date + AgeGroup + Sex + AgeGroup*Sex + Season + AgeGroup*Season | 14 | 16.21 | 0.00 |
| Date + AgeGroup + Sex + Wing Loading + AgeGroup*Sex | 11 | 16.51 | 0.00 |
| Date + AgeGroup + Sex + AgeGroup*Sex | 10 | 17.34 | 0.00 |
| Date + AgeGroup + Sex + Wing Loading + AgeGroup*Sex + Season + AgeGroup*Season | 15 | 17.81 | 0.00 |

## **11. Complete Model Results:** Mass Gain

Table S11. Candidate models explaining variation in mass gained while on a foraging absence (Mass Gain) ranked by AICc. Model selection results are presented as in Table S10.

| *Mass Gain* |  |  |  |
| --- | --- | --- | --- |
| **Model** | ***k*** | **ΔAICc** | **ωi** |
| **Date + Sex + Wing Loading** | **5** | **0.00** | **0.45** |
| **Date + Sex + Wing Loading + Season** | **6** | **1.02** | **0.27** |
| **Date + AgeGroup + Sex + Wing Loading** | **8** | **2.93** | **0.10** |
| **Date + AgeGroup + Sex + Wing Loading + Season** | **9** | **3.87** | **0.06** |
| Date + Wing Loading | 4 | 4.71 | 0.04 |
| Date + Wing Loading + Season | 5 | 5.70 | 0.03 |
| Date + AgeGroup + Sex + Wing Loading + Season + AgeGroup*Season | 12 | 6.34 | 0.02 |
| Date + AgeGroup + Sex + Wing Loading + AgeGroup*Sex | 11 | 8.02 | 0.01 |
| Date + AgeGroup + Wing Loading | 7 | 8.14 | 0.01 |
| Date + AgeGroup + Wing Loading + Season | 8 | 8.98 | 0.01 |
| Date + AgeGroup + Sex + Wing Loading + AgeGroup*Sex + Season | 12 | 8.99 | 0.01 |
| Date + AgeGroup + Wing Loading + Season + AgeGroup*Season | 11 | 11.76 | 0.00 |
| Date + AgeGroup + Sex + Wing Loading + AgeGroup*Sex + Season + AgeGroup*Season | 15 | 11.93 | 0.00 |
| Date + Sex + Season | 5 | 34.32 | 0.00 |
| Date + Season | 4 | 37.13 | 0.00 |
| Date + AgeGroup + Sex + Season | 8 | 38.24 | 0.00 |
| Date + AgeGroup + Sex + Season + AgeGroup*Season | 11 | 38.26 | 0.00 |
| Date + Sex | 4 | 40.79 | 0.00 |
| Date + AgeGroup + Season | 7 | 40.88 | 0.00 |
| Date + AgeGroup + Season + AgeGroup*Season | 10 | 41.32 | 0.00 |
| Date | 3 | 43.40 | 0.00 |
| Date + AgeGroup + Sex + AgeGroup*Sex + Season | 11 | 44.21 | 0.00 |
| Date + AgeGroup + Sex + AgeGroup*Sex + Season + AgeGroup*Season | 14 | 44.62 | 0.00 |
| Date + AgeGroup + Sex | 7 | 45.55 | 0.00 |
| Date + AgeGroup | 6 | 48.06 | 0.00 |
| Date + AgeGroup + Sex + AgeGroup*Sex | 10 | 51.64 | 0.00 |

## **12. Complete Model Results:** Absence Duration

Table S12. Candidate models explaining variation in duration of foraging absence (Absence Duration) ranked by AICc. Model selection results are presented as in Table S10.

| *Log(Absence Duration)* |  |  |  |
| --- | --- | --- | --- |
| **Model** | ***k*** | **ΔAICc** | **ωi** |
| **Date + Wing Loading + Season** | **5** | **0.00** | **0.63** |
| **Date + Sex + Wing Loading + Season** | **6** | **2.02** | **0.23** |
| Date + AgeGroup + Wing Loading + Season | 8 | 4.65 | 0.06 |
| Date + AgeGroup + Wing Loading + Season + AgeGroup*Season | 11 | 5.28 | 0.04 |
| Date + AgeGroup + Sex + Wing Loading + Season | 9 | 6.73 | 0.02 |
| Date + AgeGroup + Sex + Wing Loading + Season + AgeGroup*Season | 12 | 7.42 | 0.02 |
| Date + AgeGroup + Sex + Wing Loading + AgeGroup*Sex + Season | 12 | 12.57 | 0.00 |
| Date + AgeGroup + Sex + Wing Loading + AgeGroup*Sex + Season + AgeGroup*Season | 15 | 13.63 | 0.00 |
| Date + Season | 4 | 38.15 | 0.00 |
| Date + Wing Loading | 4 | 39.83 | 0.00 |
| Date + Sex + Season | 5 | 40.24 | 0.00 |
| Date + Sex + Wing Loading | 5 | 41.63 | 0.00 |
| Date + AgeGroup + Season | 7 | 41.71 | 0.00 |
| Date + AgeGroup + Season + AgeGroup*Season | 10 | 42.08 | 0.00 |
| Date + AgeGroup + Sex + Season | 8 | 43.88 | 0.00 |
| Date + AgeGroup + Sex + Season + AgeGroup*Season | 11 | 44.32 | 0.00 |
| Date + AgeGroup + Wing Loading | 7 | 45.21 | 0.00 |
| Date + AgeGroup + Sex + Wing Loading | 8 | 47.00 | 0.00 |
| Date + AgeGroup + Sex + AgeGroup*Sex + Season | 11 | 49.62 | 0.00 |
| Date + AgeGroup + Sex + AgeGroup*Sex + Season + AgeGroup*Season | 14 | 50.37 | 0.00 |
| Date + AgeGroup + Sex + Wing Loading + AgeGroup*Sex | 11 | 52.93 | 0.00 |
| Date | 3 | 101.62 | 0.00 |
| Date + Sex | 4 | 103.61 | 0.00 |
| Date + AgeGroup | 6 | 106.41 | 0.00 |
| Date + AgeGroup + Sex | 7 | 108.47 | 0.00 |
| Date + AgeGroup + Sex + AgeGroup*Sex | 10 | 114.18 | 0.00 |

## **13. Complete Model Results: Airspeed**

Table S13. Model selection results for Airspeed. Candidate models are ranked by AICc for outbound (top) and inbound (bottom) data. Models within ΔAICc of 4 of the top model are considered to be highly supported (in bold). The number of parameters (*k*), AICc difference from the top model (ΔAICc), and Akaike weights (*ω_i_*) are reported. All candidate models included the predictors Tailwind Component (TWC) and Date. In contrast with the main text, main effects with interactions are written out in full (e.g., AgeGroup + Season + AgeGroup*Season is written as “AgeGroup + Season + AgeGroup*Season”). Season = Breeding Season.

| *Airspeed: Outbound* | ***k*** | **ΔAICc** | | ***ω_i_*** | |
| --- | --- | --- | --- | --- | --- |
| **TWC + Date + AgeGroup + Season + AgeGroup*Season** | **11** | **0.00** | | **0.34** | |
| **TWC + Date + AgeGroup + Wing Loading + Season + AgeGroup*Season** | **12** | **0.60** | | **0.25** | |
| **TWC + Date + AgeGroup + Sex + Season + AgeGroup*Season** | **12** | **2.26** | | **0.11** | |
| **TWC + Date + Season** | **5** | **2.57** | | **0.09** | |
| **TWC + Date + AgeGroup + Sex + Wing Loading + Season + AgeGroup*Season** | **13** | **2.89** | | **0.08** | |
| **TWC + Date + Wing Loading + Season** | **6** | **3.89** | | **0.05** | |
| TWC + Date + Sex + Season | 6 | 4.67 | | 0.03 | |
| TWC + Date + Sex + Wing Loading + Season | 7 | 6.01 | | 0.02 | |
| TWC + Date + AgeGroup + Season | 8 | 7.45 | | 0.01 | |
| TWC + Date + AgeGroup + Sex + AgeGroup*Sex + Season + AgeGroup*Season | 15 | 7.72 | | 0.01 | |
| TWC + Date + AgeGroup + Sex + Wing Loading + AgeGroup*Sex + Season + AgeGroup*Season | 16 | 8.57 | | 0.00 | |
| TWC + Date + AgeGroup + Wing Loading + Season | 9 | 8.70 | | 0.00 | |
| TWC + Date + Wing Loading | 5 | 9.20 | | 0.00 | |
| TWC + Date + AgeGroup + Sex + Season | 9 | 9.60 | | 0.00 | |
| TWC + Date | 4 | 10.86 | | 0.00 | |
| TWC + Date + AgeGroup + Sex + Wing Loading + Season | 10 | 10.88 | | 0.00 | |
| TWC + Date + Sex + Wing Loading | 6 | 11.32 | | 0.00 | |
| TWC + Date + Sex | 5 | 12.97 | | 0.00 | |
| TWC + Date + AgeGroup + Sex + AgeGroup*Sex + Season | 12 | 14.16 | | 0.00 | |
| TWC + Date + AgeGroup + Wing Loading | 8 | 14.64 | | 0.00 | |
| TWC + Date + AgeGroup + Sex + Wing Loading + AgeGroup*Sex + Season | 13 | 15.65 | | 0.00 | |
| TWC + Date + AgeGroup | 7 | 16.73 | | 0.00 | |
| TWC + Date + AgeGroup + Sex + Wing Loading | 9 | 16.83 | | 0.00 | |
| TWC + Date + AgeGroup + Sex | 8 | 18.89 | | 0.00 | |
| TWC + Date + AgeGroup + Sex + Wing Loading + AgeGroup*Sex | 12 | 21.92 | | 0.00 | |
| TWC + Date + AgeGroup + Sex + AgeGroup*Sex | 11 | 23.73 | | 0.00 | |
| *Airspeed: Inbound* | ***k*** | **ΔAICc** | | ***ω_i_*** | |
| **TWC + Date + AgeGroup + Sex + Wing Loading + Season + AgeGroup*Season** | **13** | **0.00** | | **0.33** | |
| **TWC + Date + AgeGroup + Wing Loading + Season + AgeGroup*Season** | **12** | **0.48** | | **0.26** | |
| **TWC + Date + AgeGroup + Sex + Wing Loading + Season** | **10** | **1.31** | | **0.17** | |
| **TWC + Date + AgeGroup + Wing Loading + Season** | **9** | **2.01** | | **0.12** | |
| TWC + Date + AgeGroup + Sex + Wing Loading | 9 | 4.53 | | 0.03 | |
| TWC + Date + AgeGroup + Wing Loading | 8 | 5.14 | | 0.03 | |
| TWC + Date + AgeGroup + Sex + Wing Loading + AgeGroup*Sex + Season + AgeGroup*Season | 16 | 5.77 | | 0.02 | |
| TWC + Date + AgeGroup + Sex + Wing Loading + AgeGroup*Sex + Season | 13 | 7.07 | | 0.01 | |
| TWC + Date + AgeGroup + Sex + Season + AgeGroup*Season | 12 | 9.37 | | 0.00 | |
| TWC + Date + AgeGroup + Season + AgeGroup*Season | 11 | 9.46 | | 0.00 | |
| TWC + Date + AgeGroup + Sex + Wing Loading + AgeGroup*Sex | 12 | 10.15 | | 0.00 | |
| TWC + Date + Sex + Wing Loading + Season | 7 | 10.24 | | 0.00 | |
| TWC + Date + Wing Loading + Season | 6 | 10.44 | | 0.00 | |
| TWC + Date + AgeGroup + Sex + Season | 9 | 10.63 | | 0.00 | |
| TWC + Date + AgeGroup + Season | 8 | 10.92 | | 0.00 | |
| TWC + Date + AgeGroup + Sex | 8 | 12.10 | | 0.00 | |
| TWC + Date + AgeGroup | 7 | 12.36 | | 0.00 | |
| TWC + Date + Sex + Wing Loading | 6 | 13.51 | | 0.00 | |
| TWC + Date + Wing Loading | 5 | 13.64 | | 0.00 | |
| TWC + Date + AgeGroup + Sex + AgeGroup*Sex + Season + AgeGroup*Season | 15 | 15.51 | | 0.00 | |
| TWC + Date + AgeGroup + Sex + AgeGroup*Sex + Season | 12 | 16.70 | | 0.00 | |
| TWC + Date + AgeGroup + Sex + AgeGroup*Sex | 11 | 18.00 | | 0.00 | |
| TWC + Date + Season | 5 | 18.29 | | 0.00 | |
| TWC + Date + Sex + Season | 6 | 18.42 | | 0.00 | |
| TWC + Date | 4 | 19.90 | | 0.00 | |
| TWC + Date + Sex | 5 | 20.05 | | 0.00 | |
|  |  | |  | |  |
|  |  | |  | |  |

## **14. Complete Model Results: Wingbeat Frequency**

Table S14. Model selection results for Wingbeat Frequency. Model selection results are presented as in Table S13.

| *Wingbeat Frequency: Outbound* | ***k*** | **ΔAICc** | | ***ω_i_*** |
| --- | --- | --- | --- | --- |
| **TWC + Date + Sex + Season** | **6** | **0.00** | | **0.60** |
| **TWC + Date + Sex + Wing Loading + Season** | **7** | **1.92** | | **0.23** |
| TWC + Date + AgeGroup + Sex + Season | 9 | 4.15 | | 0.07 |
| TWC + Date + AgeGroup + Sex + AgeGroup*Sex + Season | 12 | 5.13 | | 0.05 |
| TWC + Date + AgeGroup + Sex + Wing Loading + Season | 10 | 6.14 | | 0.03 |
| TWC + Date + AgeGroup + Sex + Wing Loading + AgeGroup*Sex + Season | 13 | 7.27 | | 0.02 |
| TWC + Date + AgeGroup + Sex + Season + AgeGroup*Season | 12 | 10.19 | | 0.00 |
| TWC + Date + AgeGroup + Sex + AgeGroup*Sex + Season + AgeGroup*Season | 15 | 11.43 | | 0.00 |
| TWC + Date + AgeGroup + Sex + Wing Loading + Season + AgeGroup*Season | 13 | 12.27 | | 0.00 |
| TWC + Date + Sex + Wing Loading | 6 | 13.36 | | 0.00 |
| TWC + Date + AgeGroup + Sex + Wing Loading + AgeGroup*Sex + Season + AgeGroup*Season | 16 | 13.67 | | 0.00 |
| TWC + Date + Sex | 5 | 14.66 | | 0.00 |
| TWC + Date + AgeGroup + Sex + Wing Loading | 9 | 18.57 | | 0.00 |
| TWC + Date + AgeGroup + Sex | 8 | 20.04 | | 0.00 |
| TWC + Date + AgeGroup + Sex + Wing Loading + AgeGroup*Sex | 12 | 20.66 | | 0.00 |
| TWC + Date + AgeGroup + Sex + AgeGroup*Sex | 11 | 21.87 | | 0.00 |
| TWC + Date + Season | 5 | 55.03 | | 0.00 |
| TWC + Date + Wing Loading + Season | 6 | 57.07 | | 0.00 |
| TWC + Date + AgeGroup + Season | 8 | 59.13 | | 0.00 |
| TWC + Date + AgeGroup + Wing Loading + Season | 9 | 61.26 | | 0.00 |
| TWC + Date + AgeGroup + Season + AgeGroup*Season | 11 | 65.34 | | 0.00 |
| TWC + Date + Wing Loading | 5 | 66.97 | | 0.00 |
| TWC + Date | 4 | 67.31 | | 0.00 |
| TWC + Date + AgeGroup + Wing Loading + Season + AgeGroup*Season | 12 | 67.55 | | 0.00 |
| TWC + Date + AgeGroup + Wing Loading | 8 | 72.26 | | 0.00 |
| TWC + Date + AgeGroup | 7 | 72.64 | | 0.00 |
| *Wingbeat Frequency: Inbound* | ***k*** | **ΔAICc** | | ***ω_i_*** |
| **TWC + Date + Sex + Wing Loading + Season** | **7** | **0.00** | | **0.54** |
| **TWC + Date + Sex + Season** | **6** | **0.86** | | **0.35** |
| TWC + Date + AgeGroup + Sex + Wing Loading + Season | 10 | 5.05 | | 0.04 |
| TWC + Date + AgeGroup + Sex + Season | 9 | 5.97 | | 0.03 |
| TWC + Date + AgeGroup + Sex + Wing Loading + AgeGroup*Sex + Season | 13 | 7.86 | | 0.01 |
| TWC + Date + AgeGroup + Sex + AgeGroup*Sex + Season | 12 | 8.69 | | 0.01 |
| TWC + Date + Sex + Wing Loading | 6 | 9.37 | | 0.01 |
| TWC + Date + Sex | 5 | 11.31 | | 0.00 |
| TWC + Date + AgeGroup + Sex + Wing Loading + Season + AgeGroup*Season | 13 | 11.42 | | 0.00 |
| TWC + Date + AgeGroup + Sex + Season + AgeGroup*Season | 12 | 12.18 | | 0.00 |
| TWC + Date + AgeGroup + Sex + Wing Loading | 9 | 12.98 | | 0.00 |
| TWC + Date + AgeGroup + Sex + Wing Loading + AgeGroup*Sex + Season + AgeGroup*Season | 16 | 14.43 | | 0.00 |
| TWC + Date + AgeGroup + Sex | 8 | 14.95 | | 0.00 |
| TWC + Date + AgeGroup + Sex + AgeGroup*Sex + Season + AgeGroup*Season | 15 | 15.05 | | 0.00 |
| TWC + Date + AgeGroup + Sex + Wing Loading + AgeGroup*Sex | 12 | 16.06 | | 0.00 |
| TWC + Date + AgeGroup + Sex + AgeGroup*Sex | 11 | 17.96 | | 0.00 |
| TWC + Date + Wing Loading + Season | 6 | 23.87 | | 0.00 |
| TWC + Date + Season | 5 | 24.32 | | 0.00 |
| TWC + Date + AgeGroup + Wing Loading + Season | 9 | 29.12 | | 0.00 |
| TWC + Date + AgeGroup + Season | 8 | 29.60 | | 0.00 |
| TWC + Date + Wing Loading | 5 | 32.01 | | 0.00 |
| TWC + Date | 4 | 33.42 | | 0.00 |
| TWC + Date + AgeGroup + Wing Loading + Season + AgeGroup*Season | 12 | 35.70 | | 0.00 |
| TWC + Date + AgeGroup + Wing Loading | 8 | 35.95 | | 0.00 |
| TWC + Date + AgeGroup + Season + AgeGroup*Season | 11 | 36.09 | | 0.00 |
| TWC + Date + AgeGroup | 7 | 37.37 | | 0.00 |
|  |  | |  |  |

## **15. Complete Model Results:** Flap-Glide Ratio

Table S15. Model selection results for Flap-Glide Ratio. Model selection results are presented as in Table S13.

| *Flap-Glide Ratio: Outbound* | ***k*** | **ΔAICc** | **ωi** |
| --- | --- | --- | --- |
| **TWC + Date + Sex + Wing Loading + Season** | **7** | **0.00** | **0.67** |
| **TWC + Date + Wing Loading + Season** | **6** | **2.32** | **0.21** |
| TWC + Date + Sex + Season | 6 | 5.19 | 0.05 |
| TWC + Date + AgeGroup + Sex + Wing Loading + Season | 10 | 6.06 | 0.03 |
| TWC + Date + Season | 5 | 7.60 | 0.02 |
| TWC + Date + AgeGroup + Wing Loading + Season | 9 | 8.36 | 0.01 |
| TWC + Date + AgeGroup + Sex + Wing Loading + AgeGroup*Sex + Season | 13 | 10.21 | 0.00 |
| TWC + Date + AgeGroup + Sex + Wing Loading + Season + AgeGroup*Season | 13 | 10.88 | 0.00 |
| TWC + Date + AgeGroup + Sex + Season | 9 | 10.98 | 0.00 |
| TWC + Date + AgeGroup + Wing Loading + Season + AgeGroup*Season | 12 | 13.29 | 0.00 |
| TWC + Date + AgeGroup + Season | 8 | 13.44 | 0.00 |
| TWC + Date + AgeGroup + Sex + Wing Loading + AgeGroup*Sex + Season + AgeGroup*Season | 16 | 14.89 | 0.00 |
| TWC + Date + AgeGroup + Sex + AgeGroup*Sex + Season | 12 | 15.42 | 0.00 |
| TWC + Date + AgeGroup + Sex + Season + AgeGroup*Season | 12 | 15.74 | 0.00 |
| TWC + Date + AgeGroup + Season + AgeGroup*Season | 11 | 18.33 | 0.00 |
| TWC + Date + AgeGroup + Sex + AgeGroup*Sex + Season + AgeGroup*Season | 15 | 20.02 | 0.00 |
| TWC + Date + Sex | 5 | 29.07 | 0.00 |
| TWC + Date + Sex + Wing Loading | 6 | 30.80 | 0.00 |
| TWC + Date | 4 | 31.58 | 0.00 |
| TWC + Date + Wing Loading | 5 | 33.28 | 0.00 |
| TWC + Date + AgeGroup + Sex | 8 | 33.67 | 0.00 |
| TWC + Date + AgeGroup + Sex + Wing Loading | 9 | 35.49 | 0.00 |
| TWC + Date + AgeGroup | 7 | 36.33 | 0.00 |
| TWC + Date + AgeGroup + Sex + AgeGroup*Sex | 11 | 37.89 | 0.00 |
| TWC + Date + AgeGroup + Wing Loading | 8 | 38.10 | 0.00 |
| TWC + Date + AgeGroup + Sex + Wing Loading + AgeGroup*Sex | 12 | 39.70 | 0.00 |
| *Flap-Glide Ratio: Inbound* | ***k*** | **ΔAICc** | **ωi** |
| **TWC + Date + Wing Loading + Season** | **6** | **0.00** | **0.42** |
| **TWC + Date + Sex + Wing Loading + Season** | **7** | **1.45** | **0.20** |
| **TWC + Date + Season** | **5** | **3.36** | **0.08** |
| **TWC + Date + AgeGroup + Wing Loading + Season + AgeGroup*Season** | **12** | **3.50** | **0.07** |
| TWC + Date + AgeGroup + Wing Loading + Season | 9 | 4.59 | 0.04 |
| TWC + Date + AgeGroup + Sex + Wing Loading + AgeGroup*Sex + Season + AgeGroup*Season | 16 | 4.65 | 0.04 |
| TWC + Date + Sex + Season | 6 | 4.82 | 0.04 |
| TWC + Date + AgeGroup + Sex + Wing Loading + Season + AgeGroup*Season | 13 | 5.01 | 0.03 |
| TWC + Date + AgeGroup + Sex + Wing Loading + Season | 10 | 6.05 | 0.02 |
| TWC + Date + AgeGroup + Sex + Wing Loading + AgeGroup*Sex + Season | 13 | 6.22 | 0.02 |
| TWC + Date + AgeGroup + Season + AgeGroup*Season | 11 | 7.39 | 0.01 |
| TWC + Date + AgeGroup + Season | 8 | 8.02 | 0.01 |
| TWC + Date + AgeGroup + Sex + AgeGroup*Sex + Season + AgeGroup*Season | 15 | 8.63 | 0.01 |
| TWC + Date + AgeGroup + Sex + Season + AgeGroup*Season | 12 | 8.90 | 0.00 |
| TWC + Date + AgeGroup + Sex + Season | 9 | 9.50 | 0.00 |
| TWC + Date + AgeGroup + Sex + AgeGroup*Sex + Season | 12 | 9.65 | 0.00 |
| TWC + Date + Wing Loading | 5 | 12.01 | 0.00 |
| TWC + Date + Sex + Wing Loading | 6 | 13.52 | 0.00 |
| TWC + Date | 4 | 13.57 | 0.00 |
| TWC + Date + Sex | 5 | 15.08 | 0.00 |
| TWC + Date + AgeGroup + Wing Loading | 8 | 15.14 | 0.00 |
| TWC + Date + AgeGroup + Sex + Wing Loading | 9 | 16.65 | 0.00 |
| TWC + Date + AgeGroup | 7 | 16.83 | 0.00 |
| TWC + Date + AgeGroup + Sex + Wing Loading + AgeGroup*Sex | 12 | 16.93 | 0.00 |
| TWC + Date + AgeGroup + Sex | 8 | 18.34 | 0.00 |
| TWC + Date + AgeGroup + Sex + AgeGroup*Sex | 11 | 18.58 | 0.00 |

## **16. Complete Model Results:** Body Displacement

Table S16**.** Model selection results for Body Displacement. Model selection results are presented as in Table S13

| *Body Displacement: Outbound* | ***k*** | **ΔAICc** | | **ωi** | |  |
| --- | --- | --- | --- | --- | --- | --- |
| **TWC + Date + Sex + Wing Loading + Season** | **7** | **0.00** | | **0.25** | |  |
| **TWC + Date + Sex + Season** | **6** | **0.69** | | **0.18** | |  |
| **TWC + Date + AgeGroup + Sex + Wing Loading + Season** | **10** | **1.09** | | **0.14** | |  |
| **TWC + Date + Wing Loading + Season** | **6** | **1.56** | | **0.11** | |  |
| **TWC + Date + Season** | **5** | **2.1** | | **0.09** | |  |
| **TWC + Date + AgeGroup + Wing Loading + Season** | **9** | **2.92** | | **0.06** | |  |
| **TWC + Date + AgeGroup + Sex + Season** | **9** | **3.27** | | **0.05** | |  |
| **TWC + Date + AgeGroup + Sex + Wing Loading + Season + AgeGroup*Season** | **13** | **3.73** | | **0.04** | |  |
| TWC + Date + AgeGroup + Season | 8 | 4.84 | | 0.02 | |  |
| TWC + Date + AgeGroup + Wing Loading + Season + AgeGroup*Season | 12 | 5.69 | | 0.01 | |  |
| TWC + Date + AgeGroup + Sex + Season + AgeGroup*Season | 12 | 6.64 | | 0.01 | |  |
| TWC + Date + Sex + Wing Loading | 6 | 6.71 | | 0.01 | |  |
| TWC + Date + AgeGroup + Sex + Wing Loading | 9 | 6.97 | | 0.01 | |  |
| TWC + Date + AgeGroup + Sex + Wing Loading + AgeGroup*Sex + Season | 13 | 7.4 | | 0.01 | |  |
| TWC + Date + AgeGroup + Season + AgeGroup*Season | 11 | 8.29 | | 0.00 | |  |
| TWC + Date + Wing Loading | 5 | 8.54 | | 0.00 | |  |
| TWC + Date + AgeGroup + Wing Loading | 8 | 9.15 | | 0.00 | |  |
| TWC + Date + AgeGroup + Sex + AgeGroup*Sex + Season | 12 | 9.44 | | 0.00 | |  |
| TWC + Date + AgeGroup + Sex + Wing Loading + AgeGroup*Sex + Season + AgeGroup*Season | 16 | 10.2 | | 0.00 | |  |
| TWC + Date + Sex | 5 | 12.68 | | 0.00 | |  |
| TWC + Date + AgeGroup + Sex + AgeGroup*Sex + Season + AgeGroup*Season | 15 | 12.98 | | 0.00 | |  |
| TWC + Date + AgeGroup + Sex + Wing Loading + AgeGroup*Sex | 12 | 13.16 | | 0.00 | |  |
| TWC + Date | 4 | 14.3 | | 0.00 | |  |
| TWC + Date + AgeGroup + Sex | 8 | 15.32 | | 0.00 | |  |
| TWC + Date + AgeGroup | 7 | 17.16 | | 0.00 | |  |
| TWC + Date + AgeGroup + Sex + AgeGroup*Sex | 11 | 21.31 | | 0.00 | |  |
| *Body Displacement: Inbound* | ***k*** | **ΔAICc** | | **ωi** | |  |
| **TWC + Date + Season** | **5** | **0.00** | | **0.19** | |  |
| **TWC + Date** | **4** | **0.41** | | **0.16** | |  |
| **TWC + Date + Wing Loading + Season** | **6** | **0.70** | | **0.14** | |  |
| **TWC + Date + Wing Loading** | **5** | **1.47** | | **0.09** | |  |
| **TWC + Date + Sex + Season** | **6** | **2.10** | | **0.07** | |  |
| **TWC + Date + AgeGroup + Season** | **8** | **2.31** | | **0.06** | |  |
| **TWC + Date + Sex** | **5** | **2.49** | | **0.06** | |  |
| **TWC + Date + Sex + Wing Loading + Season** | **7** | **2.82** | | **0.05** | |  |
| **TWC + Date + AgeGroup + Wing Loading + Season** | **9** | **2.98** | | **0.04** | |  |
| **TWC + Date + Sex + Wing Loading** | **6** | **3.57** | | **0.03** | |  |
| **TWC + Date + AgeGroup** | **7** | **3.70** | | **0.03** | |  |
| TWC + Date + AgeGroup + Sex + Season | 9 | 4.48 | | 0.02 | |  |
| TWC + Date + AgeGroup + Wing Loading | 8 | 4.81 | | 0.02 | |  |
| TWC + Date + AgeGroup + Sex + Wing Loading + Season | 10 | 5.18 | | 0.01 | |  |
| TWC + Date + AgeGroup + Sex | 8 | 5.85 | | 0.01 | |  |
| TWC + Date + AgeGroup + Sex + Wing Loading | 9 | 6.99 | | 0.01 | |  |
| TWC + Date + AgeGroup + Season + AgeGroup*Season | 11 | 7.87 | | 0.00 | |  |
| TWC + Date + AgeGroup + Wing Loading + Season + AgeGroup*Season | 12 | 8.88 | | 0.00 | |  |
| TWC + Date + AgeGroup + Sex + AgeGroup*Sex + Season | 12 | 9.95 | | 0.00 | |  |
| TWC + Date + AgeGroup + Sex + Season + AgeGroup*Season | 12 | 10.11 | | 0.00 | |  |
| TWC + Date + AgeGroup + Sex + Wing Loading + AgeGroup*Sex + Season | 13 | 10.74 | | 0.00 | |  |
| TWC + Date + AgeGroup + Sex + Wing Loading + Season + AgeGroup*Season | 13 | 11.14 | | 0.00 | |  |
| TWC + Date + AgeGroup + Sex + AgeGroup*Sex | 11 | 11.24 | | 0.00 | |  |
| TWC + Date + AgeGroup + Sex + Wing Loading + AgeGroup*Sex | 12 | 12.46 | | 0.00 | |  |
| TWC + Date + AgeGroup + Sex + AgeGroup*Sex + Season + AgeGroup*Season | 15 | 15.75 | | 0.00 | |  |
| TWC + Date + AgeGroup + Sex + Wing Loading + AgeGroup*Sex + Season + AgeGroup*Season | 16 | 16.88 | | 0.00 | |  |
|  |  | |  | |  | |
|  |  | |  |  | |  |

## **17. Complete Model Results:** Flapping ODBA

Table S17. Model selection results for Flapping ODBA. Model selection results are presented as in Table S13.

| *Flapping ODBA: Outbound* | ***k*** | **ΔAICc** | **ωi** |
| --- | --- | --- | --- |
| **TWC + Date + Sex + Season** | **6** | **0.00** | **0.49** |
| **TWC + Date + Sex + Wing Loading + Season** | **7** | **2.12** | **0.17** |
| **TWC + Date + AgeGroup + Sex + Season** | **9** | **2.33** | **0.15** |
| TWC + Date + AgeGroup + Sex + Wing Loading + Season | 10 | 4.50 | 0.05 |
| TWC + Date + Sex | 5 | 5.04 | 0.04 |
| TWC + Date + AgeGroup + Sex | 8 | 5.72 | 0.03 |
| TWC + Date + Sex + Wing Loading | 6 | 6.00 | 0.02 |
| TWC + Date + AgeGroup + Sex + Wing Loading | 9 | 6.90 | 0.02 |
| TWC + Date + AgeGroup + Sex + AgeGroup*Sex + Season | 12 | 8.14 | 0.01 |
| TWC + Date + AgeGroup + Sex + Season + AgeGroup*Season | 12 | 8.35 | 0.01 |
| TWC + Date + AgeGroup + Sex + Wing Loading + AgeGroup*Sex + Season | 13 | 10.39 | 0.00 |
| TWC + Date + AgeGroup + Sex + Wing Loading + Season + AgeGroup*Season | 13 | 10.57 | 0.00 |
| TWC + Date + Season | 5 | 10.60 | 0.00 |
| TWC + Date + AgeGroup + Sex + AgeGroup*Sex | 11 | 11.46 | 0.00 |
| TWC + Date + Wing Loading + Season | 6 | 12.71 | 0.00 |
| TWC + Date + AgeGroup + Sex + Wing Loading + AgeGroup*Sex | 12 | 12.74 | 0.00 |
| TWC + Date + AgeGroup + Season | 8 | 13.49 | 0.00 |
| TWC + Date + AgeGroup + Sex + AgeGroup*Sex + Season + AgeGroup*Season | 15 | 14.32 | 0.00 |
| TWC + Date + AgeGroup + Wing Loading + Season | 9 | 15.66 | 0.00 |
| TWC + Date | 4 | 15.79 | 0.00 |
| TWC + Date + AgeGroup + Sex + Wing Loading + AgeGroup*Sex + Season + AgeGroup*Season | 16 | 16.61 | 0.00 |
| TWC + Date + Wing Loading | 5 | 16.85 | 0.00 |
| TWC + Date + AgeGroup | 7 | 17.13 | 0.00 |
| TWC + Date + AgeGroup + Wing Loading | 8 | 18.44 | 0.00 |
| TWC + Date + AgeGroup + Season + AgeGroup*Season | 11 | 19.63 | 0.00 |
| TWC + Date + AgeGroup + Wing Loading + Season + AgeGroup*Season | 12 | 21.87 | 0.00 |
| *Flapping ODBA: Inbound* | ***k*** | **ΔAICc** | **ωi** |
| **TWC + Date** | **4** | **0.00** | **0.23** |
| **TWC + Date + Sex** | **5** | **1.32** | **0.12** |
| **TWC + Date + Wing Loading** | **5** | **1.56** | **0.11** |
| **TWC + Date + Season** | **5** | **1.58** | **0.11** |
| **TWC + Date + AgeGroup** | **7** | **2.26** | **0.08** |
| **TWC + Date + Sex + Wing Loading** | **6** | **2.91** | **0.05** |
| **TWC + Date + Sex + Season** | **6** | **2.93** | **0.05** |
| **TWC + Date + Wing Loading + Season** | **6** | **3.05** | **0.05** |
| **TWC + Date + AgeGroup + Sex** | **8** | **3.60** | **0.04** |
| **TWC + Date + AgeGroup + Wing Loading** | **8** | **3.84** | **0.03** |
| TWC + Date + AgeGroup + Season | 8 | 4.02 | 0.03 |
| TWC + Date + Sex + Wing Loading + Season | 7 | 4.42 | 0.03 |
| TWC + Date + AgeGroup + Sex + Wing Loading | 9 | 5.22 | 0.02 |
| TWC + Date + AgeGroup + Sex + Season | 9 | 5.39 | 0.02 |
| TWC + Date + AgeGroup + Wing Loading + Season | 9 | 5.52 | 0.01 |
| TWC + Date + AgeGroup + Sex + Wing Loading + Season | 10 | 6.92 | 0.01 |
| TWC + Date + AgeGroup + Sex + AgeGroup*Sex | 11 | 7.56 | 0.01 |
| TWC + Date + AgeGroup + Sex + Wing Loading + AgeGroup*Sex | 12 | 9.24 | 0.00 |
| TWC + Date + AgeGroup + Sex + AgeGroup*Sex + Season | 12 | 9.39 | 0.00 |
| TWC + Date + AgeGroup + Season + AgeGroup*Season | 11 | 9.94 | 0.00 |
| TWC + Date + AgeGroup + Sex + Wing Loading + AgeGroup*Sex + Season | 13 | 10.98 | 0.00 |
| TWC + Date + AgeGroup + Sex + Season + AgeGroup*Season | 12 | 11.45 | 0.00 |
| TWC + Date + AgeGroup + Wing Loading + Season + AgeGroup*Season | 12 | 11.55 | 0.00 |
| TWC + Date + AgeGroup + Sex + Wing Loading + Season + AgeGroup*Season | 13 | 13.07 | 0.00 |
| TWC + Date + AgeGroup + Sex + AgeGroup*Sex + Season + AgeGroup*Season | 15 | 15.62 | 0.00 |
| TWC + Date + AgeGroup + Sex + Wing Loading + AgeGroup*Sex + Season + AgeGroup*Season | 16 | 17.34 | 0.00 |

## **18. Date and TWC Coefficient Estimates**

Table S18. Coefficient estimates for Date and Tailwind Component (TWC) from the top model set explaining variation in each flight component during the outbound and inbound periods; models appear in increasing ΔAICc. Coefficients in bold have 95% CI that exclude zero. Coefficient estimates for all other predictors are reported in the main text (Tables 4-5)

|  | **Outbound** | | | **Inbound** | | |  |
| --- | --- | --- | --- | --- | --- | --- | --- |
|  | **Fixed Effects (β [95% CI])** | | | | | |  |
| **Response Variable** | **Intercept** | **Date** | **TWC** | **Intercept** | **Date** | **TWC** | |
| **Airspeed** | **12.90 (0.16)** | 0.028 (0.069) | **-0.508 (0.026)** | **13.93 (0.21)** | -0.006 (0.005) | **-0.341 (0.029)** | |
|  | **12.86 (0.16)** | 0.016 (0.069) | **-0.505 (0.026)** | **13.83 (0.20)** | -0.006 (0.005) | **-0.336 (0.029)** | |
|  | **12.90 (0.17)** | 0.029 (0.069) | **-0.508 (0.026)** | **14.01 (0.19)** | -0.007 (0.005) | **-0.339 (0.029)** | |
|  | **12.82 (0.08)** | 0.011 (0.068) | **-0.514 (0.026)** | **12.82 (0.08)** | -0.007 (0.005) | **-0.334 (0.029)** | |
|  | **12.87 (0.17)** | 0.017 (0.069) | **-0.505 (0.026)** |  |  |  | |
|  | **12.81 (0.09)** | 0.003 (0.068) | **-0.512 (0.026)** |  |  |  | |
|  |  |  |  |  |  |  | |
| **Wingbeat Frequency** | **3.73 (0.01)** | 0.004 (0.008) | 0.002 (0.003) | **3.76 (0.02)** | **0.025 (0.011)** | -0.006 (0.004) | |
|  | **3.73 (0.01)** | 0.003 (0.008) | 0.002 (0.003) | **3.76 (0.02)** | **0.028 (0.010)** | **-0.005 (0.004)** | |
|  |  |  |  |  |  |  | |
| **Log(Flap-Glide Ratio)** | **0.15 (0.02)** | 0.007 (0.01) | **-0.010 (0.004)** | **0.22 (0.01)** | -0.001 (0.005) | **0.643 (0.029)** | |
|  | **0.13 (0.01)** | 0.005 (0.01) | **-0.011 (0.004)** | **0.21 (0.02)** | -0.002 (0.005) | **0.646 (0.029)** | |
|  |  |  |  | **0.22 (0.01)** | -0.001 (0.005) | **0.649 (0.029)** | |
|  |  |  |  | **0.23 (0.02)** | -0.002 (0.005) | **0.653 (0.029)** | |
|  |  |  |  |  |  |  | |
| **Body Displacement** | **1.54 (0.02)** | 0.000 (0.009) | **0.014 (0.004)** | **1.43 (0.01)** | -0.010 (0.010) | **0.009 (0.004)** | |
|  | **1.55 (0.02)** | 0.003 (0.009) | **0.013 (0.004)** | **1.42 (0.01)** | -0.008 (0.010) | **0.009 (0.004)** | |
|  | **1.53 (0.02)** | -0.002 (0.010) | **0.015 (0.004)** | **1.43 (0.01)** | -0.008 (0.010) | **0.010 (0.004)** | |
|  | **1.56 (0.01)** | 0.002 (0.009) | **0.015 (0.004)** | **1.42 (0.01)** | -0.006 (0.010) | **0.009 (0.004)** | |
|  | **1.56 (0.01)** | 0.004 (0.009) | **0.014 (0.004)** | **1.43 (0.02)** | -0.010 (0.010) | **0.009 (0.004)** | |
|  | **1.55 (0.02)** | -0.001 (0.010 | **0.016 (0.004)** | **1.43 (0.02)** | -0.012 (0.010) | **0.010 (0.004)** | |
|  | **1.54 (0.02)** | -0.001 (0.010) | **0.014 (0.004)** | **1.42 (0.01)** | -0.008 (0.010) | **0.009 (0.004)** | |
|  | **1.53 (0.02)** | 0.002 (0.010) | **0.015 (0.004)** | **1.43 (0.02)** | -0.008 (0.010) | **0.010 (0.004)** | |
|  |  |  |  | **1.43 (0.02)** | -0.010 (0.010) | **0.011 (0.004)** | |
|  |  |  |  | **1.42 (0.01)** | -0.006 (0.010) | **0.009 (0.004)** | |
|  |  |  |  | **1.41 (0.02)** | -0.009 (0.010) | **0.010 (0.004)** | |
|  |  |  |  |  |  |  | |
|  | **18.24 (0.12)** | 0.071 (0.073) | **0.126 (0.029)** | **17.65 (0.07)** | 0.006 (0.073) | **0.074 (0.026)** | |
| **Flapping**  **ODBA** | **18.23 (0.12)** | 0.069 (0.074) | **0.127 (0.029)** | **17.59 (0.10)** | 0.004 (0.073) | **0.076 (0.027)** | |
|  | **18.07 (0.17)** | 0.092 (0.074) | **0.125 (0.029)** | **17.65 (0.07)** | 0.017 (0.074) | **0.075 (0.026)** | |
|  |  |  |  | **17.69 (0.09)** | -0.001 (0.074) | **0.075 (0.026)** | |
|  |  |  |  | **17.47 (0.13)** | 0.015 (0.073) | **0.072 (0.028)** | |
|  |  |  |  | **17.59 (0.10)** | 0.014 (0.075) | **0.077 (0.027)** | |
|  |  |  |  | **17.63 (0.12)** | -0.003 (0.074) | **0.077 (0.027)** | |
|  |  |  |  | **17.70 (0.09)** | 0.010 (0.075) | **0.076 (0.027)** | |
|  |  |  |  | **17.40 (0.15)** | 0.012 (0.073) | **0.075 (0.028)** | |
|  |  |  |  | **17.47 (0.13)** | 0.027 (0.075) | **0.073 (0.028)** | |

## **19. Summarized Flight Performance by Sex**

Table S19. Summarized flight performance for males and females during outbound and inbound flight periods. Values are presented as model-predicted mean [95% CI]. (-) indicates that Sex did not appear as a predictor in the top model. Predicted values were calculated holding the values of all other predictors at their mean values (continuous variables) or baseline level (factors).

|  | |  | | Outbound | |  | | Inbound | |  |
| --- | --- | --- | --- | --- | --- | --- | --- | --- | --- | --- |
| Flight Component | **β** | | **Females**  **(n = 106)** | | **Males**  **(n = 103)** | **β** | **Females**  **(n = 99)** | | **Males**  **(n = 95)** | |
| Airspeed | - | | - | | - | -0.24 | - | | - | |
|  |  | |  | |  |  |  | |  | |
| Wingbeat Frequency | 0.12 | | 3.73 [3.71, 3.76] | | 3.85 [3.83, 3.87] | 0.10 | 3.73 [3.71, 3.76] | | 3.83 [3.80, 3.86] | |
|  |  | |  | |  |  |  | |  | |
| Log(Flap-Glide Ratio) | -0.04 | | 0.15 [0.12, 0.18] | | 0.11 [0.09, 0.14] | - | - | | - | |
|  |  | |  | |  |  |  | |  | |
| Body Displacement | 0.03 | | 1.54 [1.51, 1.57] | | 1.57 [1.55, 1.60] | - | - | | - | |
|  |  | |  | |  |  |  | |  | |
| Flapping ODBA | 0.51 | | 18.25 [18.02, 18.48] | | 18.76 [18.62, 18.90] | - | - | | - | |

##

## 20. Airspeed and Accelerometer-derived Flight Components

Wingbeat Frequency, Flap-Glide Ratio, and Body Displacement provide indirect measurements (from accelerometers placed on the midline of the body) of the forces imparted to the wings to generate thrust and lift. We evaluated their effect on Airspeed using a multiple linear regression model. We included Sex and TWC as predictors in the model explaining variation in Airspeed, alongside predictors Wingbeat Frequency, Flap-Glide Ratio, and Body Displacement. On the outbound, Airspeed was not influenced by Wingbeat Frequency, Flap-Glide Ratio, Body Displacement, or Sex (Table S20). On the inbound, Airspeed was also not influenced by Wingbeat Frequency, Flap-Glide Ratio, Body Displacement, or Sex (Table S20). Collins et al. (2020) also detected a complicated relationship between airspeed and wingbeat kinematics: wingbeat strength (Body Displacement here) underlies airspeed, but wingbeat frequency and airspeed are unrelated in black-legged kittiwakes (*Rissa tridactyla*).

Table S20. Output of the multiple linear regression models explaining variation in Airspeed by accelerometer-derived flight components, Sex, and Tailwind Component (TWC). Statistically significant results are in bold. Flapping ODBA was omitted as a predictor because it is highly correlated with Body Displacement (Table S3).

| **Flapping Period** | **Predictor** | **β** | **SE** | **95% CI** |
| --- | --- | --- | --- | --- |
| Outbound  n = 199 | Intercept | 12.55 | 2.48 | 7.66, 17.45 |
|  | Wingbeat Frequency | 0.08 | 0.64 | -1.18, 1.34 |
|  | Flap-Glide Ratio | 0.07 | 0.12 | -0.17, 0.31 |
|  | Body Displacement | -0.11 | 0.52 | -1.15, 0.92 |
|  | Sex | 0.03 | 0.16 | -0.31, 0.31 |
|  | TWC | **-0.52** | **0.03** | **-0.57, -0.46** |
|  |  |  |  |  |
| Inbound  n = 172 | Intercept | 18.20 | 2.57 | 13.13, 23.27 |
|  | Wingbeat Frequency | -1.00 | 0.59 | -2.16, 0.16 |
|  | Flap-Glide Ratio | -0.07 | 0.12 | -0.31, 0.16 |
|  | Body Displacement | -0.50 | 0.68 | -1.85, 0.84 |
|  | Sex | -0.13 | 0.19 | -0.50, 0.24 |
|  | TWC | **-0.34** | **0.03** | **-0.40, -0.28** |

Table S21. Individual consistency in Wingbeat Frequency, Flap-Glide Ratio, Body Displacement, and Flapping ODBA for each respective 30-minute period. 95% CIs were estimated using parametric bootstrapping (n = 1000); p values were calculated from likelihood ratio tests (Nakagawa & Schielzeth, 2010).

| **Flapping Period** | **Flight Component** | **Repeatability** | **SE** | **95% CI** |
| --- | --- | --- | --- | --- |
| Outbound  n = 209 | Wingbeat Frequency | 0.23 | 0.02 | 0.19, 0.26 |
|  | Flap-Glide Ratio | 0.16 | 0.01 | 0.14, 0.19 |
|  | Body Displacement | 0.45 | 0.02 | 0.40, 0.49 |
|  | Flapping ODBA | 0.20 | 0.02 | 0.17, 0.23 |
|  |  |  |  |  |
| Inbound  n = 194 | Wingbeat Frequency | 0.30 | 0.02 | 0.25, 0.34 |
|  | Flap-Glide Ratio | 0.16 | 0.01 | 0.14, 0.19 |
|  | Body Displacement | 0.65 | 0.02 | 0.60, 0.69 |
|  | Flapping ODBA | 0.30 | 0.02 | 0.26, 0.34 |

## 21. Airspeed and Groundspeed

Absence Duration is the denominator in the foraging outcome Mass Gain/hr, and is influenced by Groundspeed. Thus, a bird’s Groundspeed connects its rate of mass gain to flight performance through the strength of its relationship with Airspeed. In this study, we focused on the flight performance variable Airspeed because it is the direct result of the bird’s physiological performance in self-powered movement, and so is directly relevant to questions regarding the effect of age on generation of thrust and lift. However, it is not obvious that Airspeed will influence Groundspeed strongly because wind support could, in principle, make Airspeed’s effect small (Kogure et al., 2016).

We evaluated the relationship between Groundspeed and Airspeed using multiple linear regression models. Sex and TWC were included as additional predictors because sex influences flight components (main text) and wind support contributes to overall Groundspeed (Shamoun-Baranes et al., 2007). On the outbound, Groundspeed was positively influenced by Airspeed (β = 0.95, SE = 0.04, p < 0.01). On the inbound, Groundspeed was also positively influenced by Airspeed (β = 0.98, SE = 0.02, p < 0.01). Having established that Airspeed influences Groundspeed, we performed analyses with Groundspeed as a response variable; these analyses parallel those in the main text using Airspeed as a response variable. Model selection tables from Groundspeed analyses are presented below (Tables S22-S23).

## **22. Complete Model Results: Groundspeed**

Table S22. Model selection results for Groundspeed. Candidate models are ranked by AICc for outbound (top) and inbound (bottom) data. Models within ΔAICc of 4 of the top model are considered to be highly supported (in bold). The number of parameters (*k*), AICc difference from the top model (ΔAICc), and Akaike weights (*ω_i_*) are reported. All candidate models included the predictors Tailwind Component (TWC) and Date. In contrast with the main text, main effects with interactions are written out in full (e.g., AgeGroup + Season + AgeGroup*Season is written as “AgeGroup + Season + AgeGroup*Season”). Season = Breeding Season.

| *Groundspeed: Outbound* | ***k*** | **ΔAICc** | | ***ω_i_*** | |  |
| --- | --- | --- | --- | --- | --- | --- |
| **TWC + Date + Wing Loading** | **5** | **0.00** | | **0.34** | |  |
| **TWC + Date + Wing Loading + Season** | **6** | **1.54** | | **0.16** | |  |
| **TWC + Date + Sex + Wing Loading** | **6** | **1.90** | | **0.13** | |  |
| **TWC + Date** | **4** | **2.01** | | **0.13** | |  |
| **TWC + Date + Sex + Wing Loading + Season** | **7** | **3.48** | | **0.06** | |  |
| **TWC + Date + Sex** | **5** | **3.89** | | **0.05** | |  |
| TWC + Date + Season | 5 | 4.11 | | 0.04 | |  |
| TWC + Date + AgeGroup + Wing Loading | 8 | 5.65 | | 0.02 | |  |
| TWC + Date + Sex + Season | 6 | 6.01 | | 0.02 | |  |
| TWC + Date + AgeGroup + Wing Loading + Season + AgeGroup*Season | 12 | 7.38 | | 0.01 | |  |
| TWC + Date + AgeGroup + Wing Loading + Season | 9 | 7.43 | | 0.01 | |  |
| TWC + Date + AgeGroup + Sex + Wing Loading | 9 | 7.59 | | 0.01 | |  |
| TWC + Date + AgeGroup | 7 | 7.67 | | 0.01 | |  |
| TWC + Date + AgeGroup + Sex + Wing Loading + Season | 10 | 9.41 | | 0.00 | |  |
| TWC + Date + AgeGroup + Sex + Wing Loading + Season + AgeGroup*Season | 13 | 9.51 | | 0.00 | |  |
| TWC + Date + AgeGroup + Sex | 8 | 9.59 | | 0.00 | |  |
| TWC + Date + AgeGroup + Season | 8 | 9.83 | | 0.00 | |  |
| TWC + Date + AgeGroup + Season + AgeGroup*Season | 11 | 10.52 | | 0.00 | |  |
| TWC + Date + AgeGroup + Sex + Season | 9 | 11.76 | | 0.00 | |  |
| TWC + Date + AgeGroup + Sex + Wing Loading + AgeGroup*Sex | 12 | 12.32 | | 0.00 | |  |
| TWC + Date + AgeGroup + Sex + Season + AgeGroup*Season | 12 | 12.59 | | 0.00 | |  |
| TWC + Date + AgeGroup + Sex + AgeGroup*Sex | 11 | 14.04 | | 0.00 | |  |
| TWC + Date + AgeGroup + Sex + Wing Loading + AgeGroup*Sex + Season | 13 | 14.30 | | 0.00 | |  |
| TWC + Date + AgeGroup + Sex + Wing Loading + AgeGroup*Sex + Season + AgeGroup*Season | 16 | 15.08 | | 0.00 | |  |
| TWC + Date + AgeGroup + Sex + AgeGroup*Sex + Season | 12 | 16.28 | | 0.00 | |  |
| TWC + Date + AgeGroup + Sex + AgeGroup*Sex + Season + AgeGroup*Season | 15 | 17.77 | | 0.00 | |  |
| *Groundspeed: Inbound* | ***k*** | **ΔAICc** | | ***ω_i_*** | |  |
| **TWC + Date + AgeGroup + Sex + Wing Loading + Season + AgeGroup*Season** | **13** | **0.00** | | **0.48** | |  |
| **TWC + Date + AgeGroup + Sex + Wing Loading + Season** | **10** | **1.45** | | **0.23** | |  |
| **TWC + Date + AgeGroup + Wing Loading + Season + AgeGroup*Season** | **12** | **2.01** | | **0.17** | |  |
| **TWC + Date + AgeGroup + Wing Loading + Season** | **9** | **3.61** | | **0.08** | |  |
| TWC + Date + AgeGroup + Sex + Wing Loading + AgeGroup*Sex + Season + AgeGroup*Season | 16 | 6.15 | | 0.02 | |  |
| TWC + Date + AgeGroup + Sex + Wing Loading + AgeGroup*Sex + Season | 13 | 7.53 | | 0.01 | |  |
| TWC + Date + AgeGroup + Sex + Season + AgeGroup*Season | 12 | 11.00 | | 0.00 | |  |
| TWC + Date + AgeGroup + Sex + Wing Loading | 9 | 11.90 | | 0.00 | |  |
| TWC + Date + AgeGroup + Season + AgeGroup*Season | 11 | 12.40 | | 0.00 | |  |
| TWC + Date + AgeGroup + Sex + Season | 9 | 12.52 | | 0.00 | |  |
| TWC + Date + Sex + Wing Loading + Season | 7 | 12.69 | | 0.00 | |  |
| TWC + Date + AgeGroup + Wing Loading | 8 | 13.73 | | 0.00 | |  |
| TWC + Date + AgeGroup + Season | 8 | 14.07 | | 0.00 | |  |
| TWC + Date + Wing Loading + Season | 6 | 14.07 | | 0.00 | |  |
| TWC + Date + AgeGroup + Sex + AgeGroup*Sex + Season + AgeGroup*Season | 15 | 17.48 | | 0.00 | |  |
| TWC + Date + AgeGroup + Sex + Wing Loading + AgeGroup*Sex | 12 | 17.89 | | 0.00 | |  |
| TWC + Date + AgeGroup + Sex + AgeGroup*Sex + Season | 12 | 18.87 | | 0.00 | |  |
| TWC + Date + AgeGroup + Sex | 8 | 19.81 | | 0.00 | |  |
| TWC + Date + AgeGroup | 7 | 21.17 | | 0.00 | |  |
| TWC + Date + Sex + Wing Loading | 6 | 21.85 | | 0.00 | |  |
| TWC + Date + Sex + Season | 6 | 22.26 | | 0.00 | |  |
| TWC + Date + Wing Loading | 5 | 22.99 | | 0.00 | |  |
| TWC + Date + Season | 5 | 23.16 | | 0.00 | |  |
| TWC + Date + AgeGroup + Sex + AgeGroup*Sex | 11 | 26.01 | | 0.00 | |  |
| TWC + Date + Sex | 5 | 28.69 | | 0.00 | |  |
| TWC + Date | 4 | 29.45 | | 0.00 | |  |
|  |  | |  | |  | |
|  |  | |  | |  | |

## 23. Coefficient Estimates: Groundspeed

Table S23. Coefficient estimates, and SE, from the top model set explaining variation in Groundspeed during the outbound and inbound period. AgeGroup coefficients describe the mean difference in performance for Young, Old, and Oldest age classes relative to Middle Age. Sex coefficient describes the mean difference in performance for males relative to females. Breeding Season coefficient describes the mean difference in performance for birds in 2016 relative to 2015. Models are ordered from smallest to largest ΔAICc. Coefficients in bold have 95% CI that exclude zero. AgeG = AgeGroup; Season = Breeding Season.

|  |  | | | **Fixed Effects (β (SE)** | | | | | | | | |
| --- | --- | --- | --- | --- | --- | --- | --- | --- | --- | --- | --- | --- |
| **Response Variable** | | **Intercept** | **AgeG (Young)** | | **AgeG**  **(Old)** | **AgeG (Oldest)** | **AgeG*Season**  **(Young, 2016)** | **AgeG*Season**  **(Old, 2016)** | **AgeG*Season**  **(Oldest, 2016)** | **Sex**  **(Males)** | **Season**  **(2016)** | **Wing Loading** |
| **Groundspeed (outbound)** | | 11.99 (0.07) | - | | - | - | - | - | - | - | **-** | **0.03 (0.01)** |
|  |  | 11.94 (0.10) | - | | - | - | - | - | - | - | 0.12 (0.16) | **0.03 (0.01)** |
|  |  | 12.02 (0.10) | - | | - | - | - | - | - | -0.07 (0.15) | - | **0.03 (0.01)** |
|  |  | 11.99 (0.07) | - | | - | - | - | - | - | - | - | - |
|  |  | 11.98 (0.12) | - | | - | - | - | - | - | -0.07 (0.15) | 0.12 (0.16) | **0.03 (0.01)** |
|  |  | 12.03 (0.11) | - | | - | - | - | - | - | -0.07 (0.15) | **-** | - |
|  | |  |  | |  |  |  |  |  |  |  |  |
| **Groundspeed (inbound)** | | 12.79 (0.21) | -0.27 (0.27) | | -0.29 (0.27) | **-0.67 (0.33)** | 0.73 (0.54) | -0.62 (0.43) | -0.61 (0.48) | **-0.33 (0.16)** | **0.81 (0.29)** | **0.04 (0.01)** |
|  |  | 12.88 (0.19) | -0.21 (0.24) | | **-0.53 (0.21)** | **-0.96 (0.24)** | - | - | - | **-0.33 (0.16)** | **0.62 (0.18)** | **0.04 (0.01)** |
|  |  | 12.61 (0.20) | -0.26 (0.27) | | -0.27 (0.27) | **-0.66 (0.33)** | 0.73 (0.54) | -0.66 (0.43) | -0.60 (0.49) | - | **0.82 (0.29)** | **0.04 (0.01)** |
|  |  | 12.71 (0.17) | -0.20 (0.24) | | **-0.53 (0.21)** | **-0.94 (0.24)** | - | - | - | - | **0.62 (0.18)** | **0.04 (0.01)** |


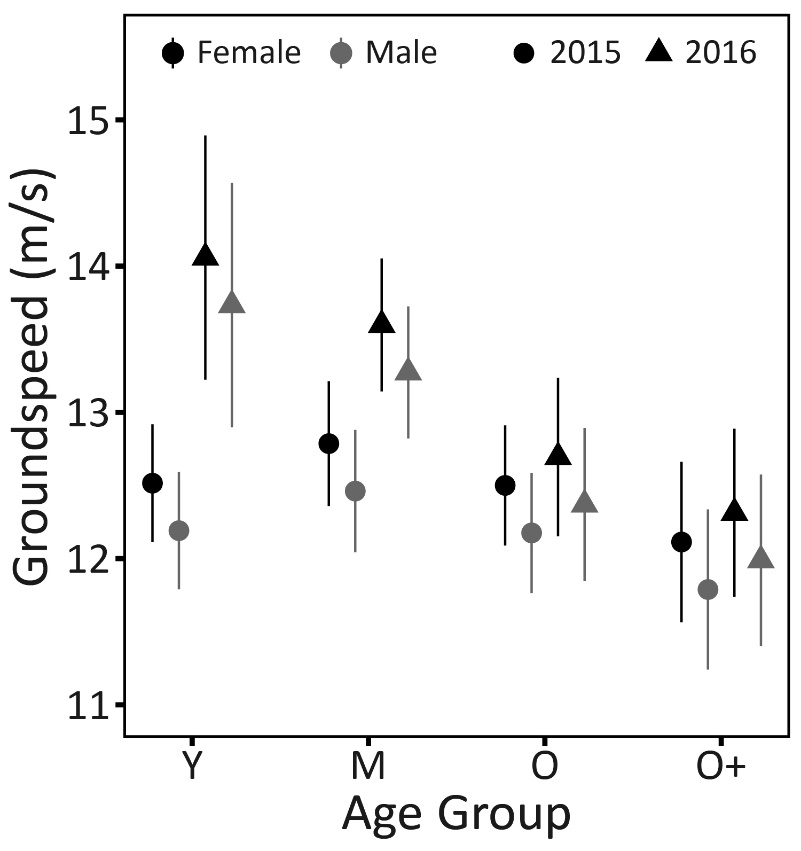


Figure S12. Age effects on Groundspeed (m/s) during the inbound period in Young (Y), Middle Age (M), Old (O), and Oldest (O+) Nazca boobies. Large points are the mean values ± 95% CI, calculated holding the values of all other predictors at their mean value (continuous covariates) or baseline level (factors).

## **24. Effects of AgeGroup and Sex on Body Mass and Fledging Success**

We expected lower Mass Gains with age during a foraging absence to yield corresponding lower body mass in Old and Oldest birds (relative to Middle Age birds) at their return to incubation. We tested this idea in a *post hoc* analysis using mass measurements at deployment and return from the 209 tagged birds. We used a general linear model (Gaussian errors) to examine the relationship between two response variables: mass at departure (MassPre, see “Correction Made to Initial Body Mass”) and mass at return (MassPost), and predictors AgeGroup and Sex. Because Breeding Season did not appear within the top model set for Mass Gain, we did not include it in these *post hoc* analyses. As expected for MassPre and MassPost, Sex was a significant predictor of body mass (MassPre: p < 0.01; MassPost: p < 0.01; Table S24). Only the Oldest birds had a significantly lower mass at departure compared to Middle Age birds (p < 0.05), whereas Old and Oldest birds differed significantly from Middle Age birds (both p < 0.01). We also examined the additive effects of AgeGroup, Sex, and Breeding Season on Fledging Success, defined as the probability of raising an offspring to independence, given a breeding attempt. Fledging Success is binomial: either parents fledged an offspring (“1”) or did not (“0”); therefore, we used a generalized linear model with binomial errors, a logit link function, and predictors AgeGroup, Sex, and Breeding Season. AgeGroup affected Fledging Success, with Old and Oldest birds having negligible reproductive success compared to Middle Age birds (Table S24; maintext Fig. 4A), concurring with the results of Tompkins & Anderson (2019).

Table S24**.** Coefficient estimates, and SE, describing the effect of age on Fledging Success, mass at departure (MassPre) and mass at return (MassPost) from a foraging absence. AgeGroup coefficients describe the change in each response variable in Young, Old, and Oldest birds relative to Middle Age. Sex coefficient describes the mean difference in performance for males relative to females. Coefficients in bold have 95% CI that exclude zero.

|  | **Fixed Effects (β (SE))** | | | | | |
| --- | --- | --- | --- | --- | --- | --- |
| **Response Variables** | **Intercept** | **AgeGroup**  **(Young)** | **AgeGroup**  **(Old)** | **AgeGroup**  **(Oldest)** | **Sex**  **(Males)** | **Season (2016)** |
| **Fledging Success** | **0.06 (0.31)** | **-0.94 (0.42)** | **-2.06 (0.49)** | **-2.56 (0.67)** | -0.42 (0.35) | **-1.17 (0.40)** |
| **MassPre** | **1.88 (0.02)** | -0.03 (0.03) | -0.03 (0.02) | **-0.06 (0.03)** | -**0.27 (0.02)** | - |
| **MassPost** | **2.26 (0.02)** | -0.04 (0.03) | **-0.09 (0.03)** | **-0.11 (0.03)** | **-0.31 (0.02)** | **-** |

## 25. Environmental Variation in the Nazca Booby Foraging Area

In our Discussion, we summarize changes in the physical oceanic environment used by foraging Nazca boobies for the two years of our study (2015 versus 2016 Breeding Seasons). We accessed 8-day composite values of SST and chlorophyll *a* (Chl *a*, mg m^−3^) from the NOAA ERDDAP servers using the *rerddap* R package (Chamberlain, 2016). Oceanographic data were extracted for Nov. 29, 2015 to Jan. 22, 2016 and Nov. 14, 2016 to Jan. 23, 2017 during the two breeding seasons, for the Nazca booby foraging area from the Aqua MODIS satellite, NPP, 0.025 degrees, Pacific Ocean Lon ± 180 ('erdMBsstd8day_LonPM180'). The Nazca booby foraging area was defined as a polygon extending to the 95% quantile of longitudes and latitudes reached by Nazca boobies during this study (coordinates: 89.62°W to 86.28°W; 2.82°S to 0.32°N), which overlapped considerably with foraging areas observed by Zavalaga et al. (2012) during chick rearing. We averaged SST and Chl *a* during the November-January study period. The average November-January sea surface temperature was 2.7°C warmer (2015: 26.3°C, 2016: 23.6°C), and chlorophyl1 *a* concentration was 0.23 mg m^-3^ lower (2015: 0.19 mg m^−3^ in, 2016: 0.42 mg m^−3^) during the 2015 breeding season than in the following year.

The Nov.-Jan. Sea Surface Temperature Anomaly (SSTA) statistic referenced in the Discussion is for the NINO3 region (5°N to 5°S, 150°W to 90°W) representing the change in sea surface temperature in winter 2015-16 relative to the 1981-2010 mean conditions. Data were downloaded from https://psl.noaa.gov/gcos_wgsp/Timeseries/Nino3/ on January 12, 2021.

# REFERENCES

Anderson, D. J. (1993). Masked Booby (*Sula dactylatra*). *The Birds of North America Online*. doi:10.2173/bna.73

Anderson, D. J., & Ricklefs, R. E. (1987). Radio-tracking masked and blue-footed boobies (*Sula* spp.) in the Galápagos Islands. *National Geographic Research*, *3*(2), 152–163.

Ballance, L. T. (1995). Flight energetics of free-ranging red-footed boobies (*Sula sula*). *Physiological Zoology*, *68*(5), 887–914. doi:10.1086/physzool.68.5.30163937

Burnham, K. P., & Anderson, D. R. (2002). *Model selection and Multimodel Inference: A Practical Information-Theoretic Approach* (2nd ed.). New York: Springer.

Burnham, K. P., Anderson, D. R., & Huyvaert, K. P. (2011). AIC model selection and multimodel inference in behavioral ecology: some background, observations, and comparisons. *Behavioral Ecology and Sociobiology*, *65*(1), 23–35. doi:10.1007/s00265-010-1029-6

Chamberlain, S. (2016). rerddap: General purpose client for ‘ERDDAP’servers. R package version 0.3. 4. Retrieved from https://github.com/ropensci/rerddap

Cherel, Y., Robin, J.-P., & Maho, Y. Le. (1988). Physiology and biochemistry of long-term fasting in birds. *Canadian Journal of Zoology*, *66*(1), 159–166.

Chimienti, M., Cornulier, T., Owen, E., Bolton, M., Davies, I. M., Travis, J. M. J., & Scott, B. E. (2016). The use of an unsupervised learning approach ^for^ characterizing latent behaviors in accelerometer data. *Ecology and Evolution*, *6*(3), 727–741. doi:10.1002/ece3.1914

Collins, P. M., Green, J. A., Elliott, K. H., Shaw, P. J. A., Chivers, L., Hatch, S. A., & Halsey, L. G. (2020). Coping with the commute: behavioural responses to wind conditions in a foraging seabird. *Journal of Avian Biology*, *51*(4), 1–11. doi:10.1111/jav.02057

Collins, P. M., Green, J. A., Warwick-Evans, V., Dodd, S., Shaw, P. J. A., Arnould, J. P. Y., & Halsey, L. G. (2015). Interpreting behaviors from accelerometry: a method combining simplicity and objectivity. *Ecology and Evolution*, *5*(20), 4642–4654. doi:10.1002/ece3.1660

Dodge, S., Bohrer, G., Weinzierl, R., Davidson, S. C., Kays, R., Douglas, D., … Wikelski, M. (2013). The environmental-data automated track annotation (*Env-DATA*) system: linking animal tracks with environmental data. *Movement Ecology*, *1*(3), 1–14. doi:10.1186/2051-3933-1-3

Elliott, K. H., Le Vaillant, M., Kato, A., Gaston, A. J., Ropert-Coudert, Y., Hare, J. F., … Croll, D. (2014). Age-related variation in energy expenditure in a long-lived bird within the envelope of an energy ceiling. *The Journal of Animal Ecology*, *83*, 136–46. doi:10.1111/1365-2656.12126

Heerenbrink, M. K., Johansson, L. C., & Hedenstrom, A. (2015). Power of the wingbeat: modelling the effects of flapping wings in vertebrate flight. *Proceedings of the Royal Society A: Mathematical, Physical and Engineering Sciences*, *471*(2177), 20140952. doi:10.1098/rspa.2014.0952

Kogure, Y., Sato, K., Watanuki, Y., Wanless, S., & Daunt, F. (2016). European shags optimize their flight behavior according to wind conditions. *Journal of Experimental Biology*, *219*(3), 311–318. doi:10.1242/jeb.131441

Lecomte, V. J., Sorci, G., Cornet, S., Jaeger, A., Faivre, B., Arnoux, E., … Orians, G. H. (2010). Patterns of aging in the long-lived wandering albatross. *Proceedings of the National Academy of Sciences of the United States of America*, *107*(14), 6370–6375. doi:10.1073/pnas.0911181107

Nakagawa, S., & Schielzeth, H. (2010). Repeatability for Gaussian and non-Gaussian data: A practical guide for biologists. *Biological Reviews*, *85*(4), 935–956. doi:10.1111/j.1469-185X.2010.00141.x

Patterson, A., Gilchrist, H. G., Chivers, L., Hatch, S., & Elliott, K. (2018). A comparison of techniques for classifying behavior from accelerometers for two species of seabird. *Ecology and Evolution*, *9*(6), 3030–3045. doi:10.1002/ece3.4740

Pennycuick, C. J. (1989). *Bird Flight Performance*. New York: Oxford University Press.

Prince, P. A., Ricketts, C., & Thomas, G. (1981). Weight loss in incubating albatrosses and its implications for their energy and food requirements. *The Condor*, *83*(3), 238–242.

Ropert-Coudert, Y., Daunt, F., Kato, A., Ryan, P. G., Lewis, S., Kobayashi, K., … Wanless, S. (2009). Underwater wingbeats extend depth and duration of plunge dives in northern gannets *Morus bassanus*. *Journal of Avian Biology*, *40*(4), 380–387. doi:10.1111/j.1600-048X.2008.04592.x

Safi, K., Kranstauber, B., Weinzierl, R., Griffin, L., Rees, E. C., Cabot, D., … Bohrer, G. (2013). Flying with the wind: scale dependency of speed and direction measurements in modelling wind support in avian flight. *Movement Ecology*, *1*(4), 1–13. doi:10.1186/2051-3933-1-4

Shamoun-Baranes, J., van Loon, E., Liechti, F., & Bouten, W. (2007). Analyzing the effect of wind on flight: pitfalls and solutions. *Journal of Experimental Biology*, *210*(1), 82–90. doi:10.1242/jeb.02612

Shepard, E. L. C., Wilson, R. P., Halsey, L. G., Quintana, F., Laich, A. G., Gleiss, A. C., … Norman, B. (2008). Derivation of body motion via appropriate smoothing of acceleration data. *Aquatic Biology*, *4*(3), 235–241. doi:10.3354/ab00104

Tompkins, E. M., & Anderson, D. J. (2019). Sex-specific patterns of senescence in Nazca boobies linked to mating system. *Journal of Animal Ecology*, *88*(7), 986–1000. doi:10.1111/1365-2656.12944

Van Oordt, F., Torres-Mura, J. C., & Hertel, F. (2018). Ecomorphology and foraging behaviour of Pacific boobies. *Ibis*, *160*(2), 313–326. doi:10.1111/ibi.12545
